# The draft genome sequence of forest musk deer (*Moschus berezovskii*)

**DOI:** 10.1093/gigascience/giy038

**Published:** 2018-04-09

**Authors:** Zhenxin Fan, Wujiao Li, Jiazheng Jin, Kai Cui, Chaochao Yan, Changjun Peng, Zuoyi Jian, Ping Bu, Megan Price, Xiuyue Zhang, Yongmei Shen, Jing Li, Wenhua Qi, Bisong Yue

**Affiliations:** 1 Key Laboratory of Bioresources and Ecoenvironment (Ministry of Education), College of Life Sciences, Sichuan University, Chengdu 610064, People's Republic of China; 2 Sichuan Engineering Research Center for Medicinal Animals, Xichang 615000, People's Republic of China; 3 College of Life Science and Engineering, Chongqing Three Gorges University, Chongqing 404100, People's Republic of China

**Keywords:** forest musk deer, whole genome sequencing, genome assembly, annotation, phylogeny

## Abstract

**Background:**

The forest musk deer, *Moschus berezovskii*, is one of seven musk deer (*Moschus* spp.) and is distributed in Southwest China. Akin to other musk deer, the forest musk deer has been traditionally and is currently hunted for its musk (i.e., global perfume industry). Considerable hunting pressure and habitat loss have caused significant population declines. Consequently, the Chinese government commenced captive breeding programs for musk harvesting in the 1950s. However, the prevalence of fatal diseases is considerably restricting population increases. Disease severity and extent are exacerbated by inbreeding and genetic diversity declines in captive musk deer populations. It is essential that knowledge of captive and wild forest musk deer populations' immune system and genome be gained in order to improve their physical and genetic health. We have thus sequenced the whole genome of the forest musk deer, completed the genomic assembly and annotation, and performed preliminary bioinformatic analyses.

**Findings:**

A total of 407 Gb raw reads from whole-genome sequencing were generated using the Illumina HiSeq 4000 platform. The final genome assembly is around 2.72 Gb, with a contig N50 length of 22.6 kb and a scaffold N50 length of 2.85 Mb. We identified 24,352 genes and found that 42.05% of the genome is composed of repetitive elements. We also detected 1,236 olfactory receptor genes. The genome-wide phylogenetic tree indicated that the forest musk deer was within the order Artiodactyla, and it appeared as the sister clade of four members of Bovidae. In total, 576 genes were under positive selection in the forest musk deer lineage.

**Conclusions:**

We provide the first genome sequence and gene annotation for the forest musk deer. The availability of these resources will be very useful for the conservation and captive breeding of this endangered and economically important species and for reconstructing the evolutionary history of the order Artiodactyla.

## Data Description

### Background

The seven musk deer species of the genus *Moschus* are endemic to Asia. They are currently listed in Appendix II in CITES and in Category I of the State Key Protected Wildlife List of China [1–3]. All musk deer species are considered to be globally threatened, with six being listed as endangered and one as vulnerable by the International Union for Conservation of Nature [4]. *Moschus* is the only extant genus of Moschidae, and musk deer are considered to be primitive deer. The genus of musk deer is characterized by the musk secreted by the scent glands of adult males [5]. The forest musk deer (*Moschus berezovskii*) is one of the five recognized musk deer species of China and have historically been distributed in Southwest China [6, 7]. The forest musk deer has been listed as globally endangered, as critically endangered on the 2015 China Red List, and is also on the State Key Protected Wildlife List of China [4].

Musk deer have been hunted for thousands of years, as the musk has been widely used in traditional Chinese medicines. In the last two centuries, hunting of all musk deer species significantly increased because of the commercial value of musk, which was an essential basis for perfume manufacture [5]. Since the 1950s, populations of forest musk deer have declined dramatically from poaching for the musk pods (i.e., entire gland) and significant habitat destruction [3, 6, 8]. As a consequence, since the early 1950s the Chinese government has encouraged musk-using enterprises to participate in artificial breeding programs [9]. The musk can be collected from male musk deer in these captive populations without harvesting individuals, further enhancing the commercial and conservation value of captive populations.

The captive population of the forest musk deer is the largest among all the musk deer species [2, 10]. The Miyaluo farming population in Sichuan Province (China) was one of the earliest established captive breeding populations. This population had grown rapidly to approximately 400 in 2010 [10]. However, the prevalence of fatal diseases is considerably restricting population increases [11]. Common diseases of forest musk deer in the Miyaluo population are dyspepsia, pneumonia, metritis, urinary stones, and abscesses, with abscesses being one of the most prevalent causes of death [7]. Disease severity and extent are exacerbated by inbreeding and genetic diversity declines in this and other captive musk deer populations [7, 10].

Although the transcriptomes of captive forest musk deer had been reported [12, 13], there is no complete genome sequence. This information is essential for the genetic management and disease prevention of captive and wild forest musk deer populations and for improving knowledge of its immune system. Thus, we sequenced the whole genome of the forest musk deer, subsequently completed the genomic assembly and annotation, and performed preliminary bioinformatic analyses, such as the phylogenetic tree.

### Sample information and sequencing

A thigh muscle sample was collected from a Miyaluo male forest musk deer that naturally died (Sichuan Province, China) in 2015. We extracted genomic DNA from the muscle sample using the Qiagen DNeasy blood and tissue kit (Qiagen, Valencia, USA) following the manufacturer's protocol. We constructed six insert size libraries: 230 bp, 500 bp, 2 kb, 5 kb, 10 kb, and 15 kb. These libraries were sequenced using the Illumina HiSeq 4000 platform at Novogene (Beijing, China). A total of 407 Gbof raw data were generated. After filtering out low quality reads, duplicates, and adaptors, about 360 Gbof high-quality reads were retained for genome assembly (Table 1).

**Table 1:** Genome sequencing information

		Raw data		Clean data	
Insert size (bp)	Read length (bp)	Total bases (Gb)	Sequencing depth (x)	Total bases (Gb)	Sequencing depth (x)
230	125	135.76	46.02	125.96	42.70
500	125	102.51	34.75	88.52	30.01
2,000	125	59.0	20.00	50.16	17.00
5,000	125	51.57	17.48	46.39	15.73
10,000	125	28.16	9.55	24.67	8.36
15,000	125	30.34	10.28	28.14	9.54
Total		407.34	138.08	363.84	123.34

Note: Genome size is 2.95 Gb.

### Genome assembly and evaluation

We used GCE (version 1.0) to performed k-mer (17-mer) analysis by short insert size library reads before assembly; the forest musk deer genome size was estimated to be 2.95 Gb(Supplementary Fig. S1). The assembly was first generated using SOAPdenovo2 (SOAPdenovo2, RRID:SCR_014986) [14] with the parameters set as “all -d 2 –M 2 –k 35.” Intrascaffoldgaps were filled using Gapcloser (version 1.12) with reads from 230 bpand 500 bplibraries, and then SSPACE version 3.0 (SSPACE, RRID:SCR_005056) [15] was used to build super scaffolds. After scaffolding with SSPACE, we used Gapcloser to fill gaps. Finally, we obtained the forest musk deer genome with a size of 2.72 Gb(all the sequences with length shorter than 300 bpwere removed) with 125.7 Mbgap sequences unsolved. The N50s of contigs and scaffolds of the forest musk deer genome were 22.6 kband 2.85 Mb, respectively (Table 2).

**Table 2:** Statistics of the final assembly of forest musk deer genome

Genome assembly	Numbers
Contig N50 (Kb)	22.6
Scaffold N50 (Mb)	2.85
Longest scaffold (Mb)	18.69
Scaffold number	79 206
GC content	40%
Total length (Gb)	2.72

We used Benchmarking Universal Single-Copy Orthologs (BUSCO) version 3.0 (BUSCO, RRID:SCR_015008) to evaluate the genome complement. BUSCO results showed that 84.5% of the eukaryotic single-copy genes were captured (Supplementary Table S1). Furthermore, we downloaded musk gland RNA sequencing RNA-seq data (SRA accession: SRR2098995 and SRR2098996) of forest musk deer from the National Center for Biotechnology Information (NCBI) to evaluate the assembly [13]. We found that 99.3% of the total paired-end (PE) reads could be aligned (92.73% aligned concordantly) to the assembled forest musk deer genome with Bowtie2 (version 2.2.5) [16].

### Annotation

We combined the *de novo*, homology-based, and transcriptome-based prediction to identify protein-coding genes in the forest musk deer genome. The software Augustus version 3.2.1 (Augustus: Gene Prediction, RRID:SCR_008417) [17] was used for *de novo* prediction based on the parameter trained for forest musk deer. For homology prediction, protein sequences from four mammals (human, pig, sheep, and cattle) were analyzed with TBLASTN (BLAST version 2.2.26) against the forest musk deer genome. Potential gene regions were joined using SOLAR (version 0.9.6) [18], and the coding sequence with 500 bpflanking sequence was cut down and re-aligned using GeneWise (GeneWise, RRID:SCR_015054) version 2.4.1 with parameters “- sum—genesf -gff” [19]. For transcriptome-based prediction, musk gland RNA-seq data were assembled using Trinity (Trinity, RRID:SCR_013048) with genome guide and *de novo* mode, respectively. The gene structures were obtained using PASA pipeline (version 2.0.2) [20]. We used EVM (version 1.1.1) to integrate the above evidence and obtained a consensus gene set [21]. Apollo (version 1.11.6) was performed to manually inspect gene structure in scaffolds of sizes larger than 1 Mb to gain a more accurate gene structure. We consequently found 24,352 genes predicted to be present in the forest musk deer genome. We also provide the length of genes in Supplementary Table S2.

**Figure 1:**
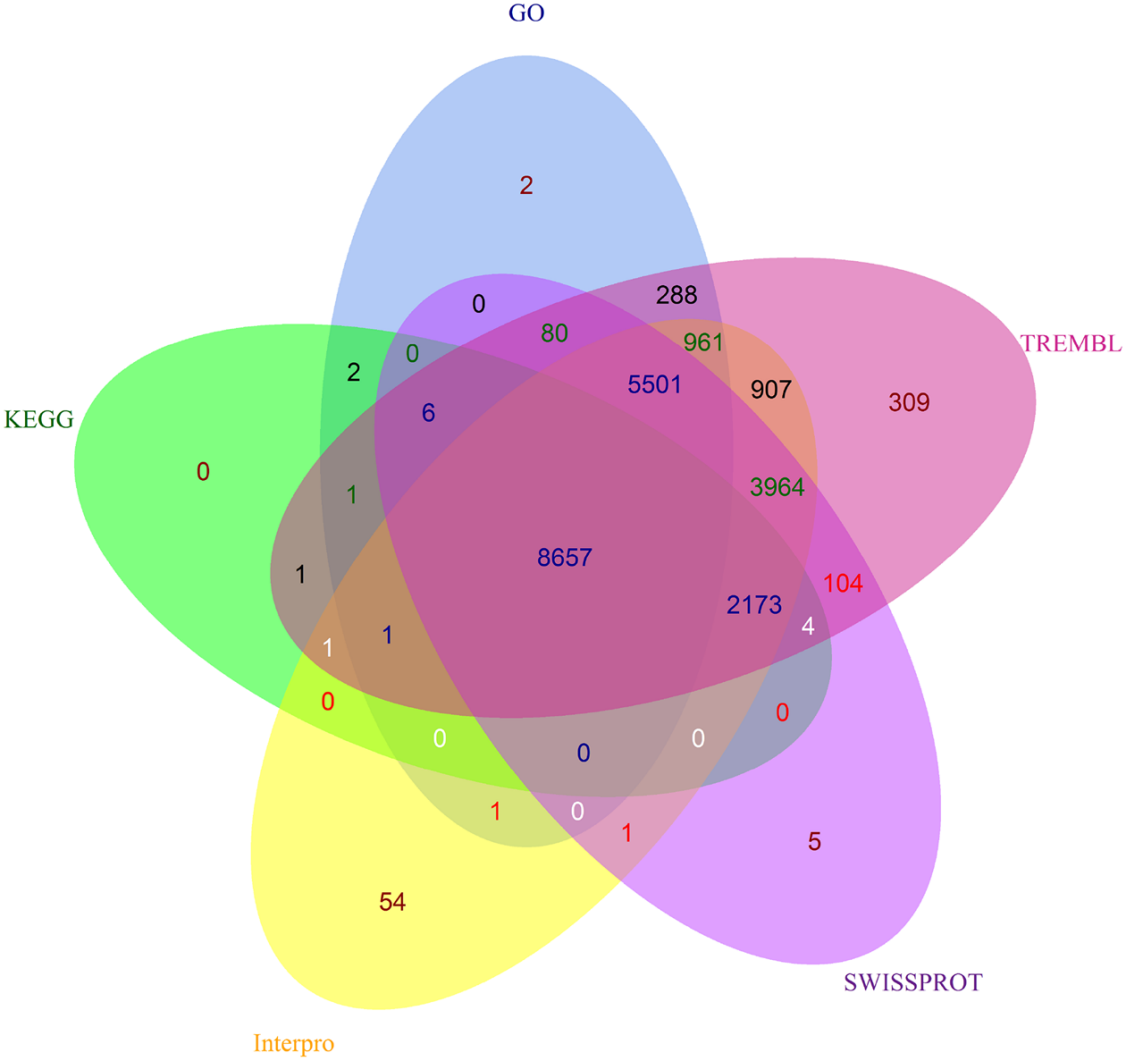
Functional annotation statistics. Venn diagram illustrating distribution of high-score matches of the functional annotation in forest musk deer genome from five public databases.

Functional annotation of forest musk deer genes was undertaken based on the best match derived from the alignments to proteins annotated in Swiss-Prot and TrEMBL databases [22]. Functional annotation used Basic Local Alignment Search Tool for Proteins tools with the same E-value cutoff of 1E-5. We also annotated proteins against the NCBI nonredundant (nr) protein database. The outputs of blast searching against the NCBI nr protein database were imported into BLAST2GO (B2G4PIPE v2.5) for Gene Ontology (GO) [23] term mapping. Term mapping used annotated motifs and domains using InterProScan (InterProScan, RRID:SCR_005829), interproscan-5.18–57.0 [24], by searching against publicly available databases. To find the best match for each gene, KEGG pathway maps were used by searching KEGG databases [25] through the KEGG Automatic Annotation Server (KAAS) using the bidirectional best hit (BBH) method. In total, 23,023of 24,352 (94.5%) protein-coding genes were searched within the publicly available functional databases of TrEMBL, Swiss-Prot, Interpro, GO, and KEGG. Of which, 22,696 (93.20% TrEMBL), 18,771 (77.08% Swiss-Prot), 22,221 (91.12% Interpro), 15,736 (64.62% GO), and 10,846 (44.54% KEGG) genes showed significant similarity matches (Fig. 1; Table 3). The functional comparisons with two closely related species (cattle and sheep) for GO classification were submitted to the Web Gene Ontology Annotation Plot (WEGO) [26] (Fig. S2).

**Table 3:** Functional annotation statistics of the forest musk deer genome by various methods

	Database	Number	Percent (%)
Total		24 352	100.00
	Swiss-Prot	18 771	77.08
	TrEMBL	22 696	93.20
Annotated	KEGG	10 846	44.54
	Interpro	22 221	91.12
	GO (BLAST2GO)	15 736	64.62
	GO (Interproscan)	14 815	60.84
Unannotated		1329	5.77

### Repetitive sequences and transposable elements

Transposable elements (TEs) and other repeats make up a substantial fraction of mammalian genomes and contribute to gene or genome evolution [27]. The TE content, type, copy number, subfamily, and divergence rate were investigated in the forest musk deer genome based on two strategies: the library-based strategy of RepeatMasker (RepeatMasker, RRID:SCR_012954) [28] and the *de novo*-based strategy of RepeatScout (RepeatScout, RRID:SCR_014653) [29]. The forest musk deer genome has large numbers of TEs, comprising 42.05% of the genome (Supplementary Table S3), which is similar to those of cattle (46.5%) [27] and goats (42.2%) [30]. The 23 types of TEs have been grouped for the 4 types of TEs, including DNA transposons, LTR, LINE, and SINE retrotransposons (Supplementary Fig. S3). The LINEs were the most common repeats in the forest musk deer genome, followed by SINEs > LTR > DNA. We also analyzed the degree of divergence for each type of TE in the forest musk deer genome. We found there was a recent burst of activity involving LINE transposons and a second, older burst of activity of LTR and DNA transposons (Supplementary Fig. S3).

A total of 542,135 microsatellites (simple sequence repeats [SSRs]) were identified using software MSDB [31] in the forest musk deer genome assembly (Supplementary Table S4), which accounted for 0.45% of its whole genome length. Mononucleotide SSRs were the most abundant category, accounting for 41.75% of all SSRs, followed by di- > tri- > tetra- > penta- > hexa nucleotide SSRs (Supplementary Table S4).

### Gene families

To estimate species-specific and shared genes in the forest musk deer in comparison to 10 mammal species, we used orthoMCL [32] to define the orthologous genes. We downloaded the genomes and gene annotations of the 10 additional species (human, horse, dog, cattle, mouse, yak, sheep, Tibetan antelope, alpaca, and pig) from Ensembl [33] or NCBI (Supplementary Table S5). We identified 18,855 homologous gene families shared by forest musk deer and the 10 additional species, 221 gene families that were specific to forest musk deer, and 2003 gene families found in the 10 additional species but not in the forest musk deer (Supplementary Fig. S4). In addition, we found 5,372 one-to-one orthologous genes within forest musk deer and the other 10 species, which was used in phylogenetic analyses. In addition, we detected olfactory receptor (OR) genes in the forest musk deer genome by orfam [34] since they formed the largest gene family in mammalian genomes [35]. We identified 1,236 OR genes, which included 866 intact, 266 pseudogenes, and 104 truncated genes.

### Phylogenetic analysis

**Figure 2:**
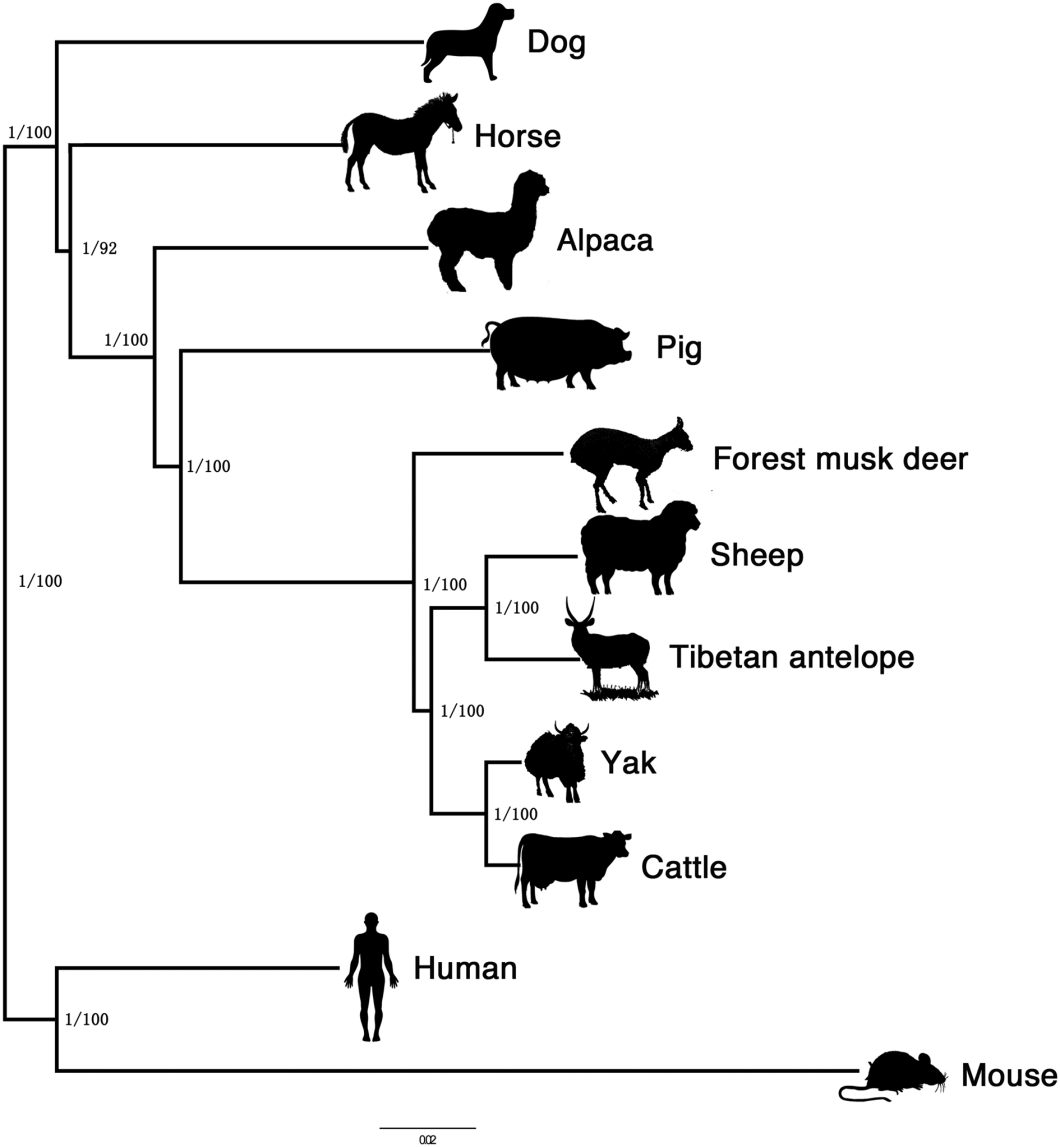
Genome-wide phylogenetic trees. We constructed the phylogenetic trees based on Bayesian inference and maximum likelihood analyses with 5,372 one-to-one orthologous genes between the forest musk deer and 10 other species.

We constructed the phylogenetic trees based on Bayesian inference [36] and maximum likelihood [37, 38] analyses with the discovered 5,372 one-to-one orthologous genes (Supplementary Notes). All the different methods generated the same topology and obtained the well-supported phylogenetic tree (Fig. 2). The forest musk deer was within the suborder Ruminantia and order Artiodactyla, and it appeared as the sister clade of four members of family Bovidae (sheep, yak, cattle, and Tibetan antelope). Since we do not have high-quality genome sequences for species within family Cervidae, the relationships between Moschidae, Cervidae, and Bovidae at the genomic level are tentative and need further investigation.

## Conclusions

Here, we report the first draft assembly of the forest musk deer genome, a species that is of particular importance to Chinese ecology, biodiversity conservation, economy, and medicine. The availability of the genome and these results will be very useful for the conservation and captive breeding of this endangered and economically important species and for reconstructing the evolutionary history of the order Artiodactyla.

## Availability of supporting data

The DNA sequencing data have been deposited into the NCBI Sequence Read Archive under the ID PRJNA317652. Other supporting data, including the assembled genome, gene annotations, and BUSCO results, are available via the *GigaScience* repository, GigaDB [39].

## Additional files

Figure S1: K-mer (k = 17) distributions in forest musk deer genome.

Figure S2: GO comparative analysis and functional classification between forest musk deer, sheep and cattle.

Figure S3: Distribution of divergence of each type of TEs in forest musk deer genome. The divergence rate was calculated between the identified TE elements in the genome and the consensus sequence in the TE library used. SINEs: Short interspersed elements. LINEs: Long interspersed elements. LTR: Long terminal repeat retrotransposon.

Figure S4: Protein orthology comparison between different genomes. There were forest musk deer (*Moschus bweezovskii*), cattle (*Bos taurus*), yak (*Bos grunniens*), sheep (*Ovis aries*), Tibetan antelope (*Pantholops hodgsonii*), alpaca (*Vicugna pacos*), and pig (*Sus scrofa*), which representing Artiodactyla; human (*Homo sapiens*, Primates), horse (*Equus caballus*, Perissodactyla), and dog (*Canis lupus familiaris*, Carnivora), mouse (*Mus musculus*, Rodentia). For each animal, proteins were represented by bars and were classified based on orthoMCL analysis. Single_copy (green) included the common orthologs with the same number of copies in different species; Multi_copy (red) included the common orthologs with different copy numbers in different species; Unique (magenta) included the orthologs that were only in one species; Unclustered genes (yellow) included the genes that could not be clustered into known gene families; Other (blue) included the genes that could be clustered into known gene families, but were not belonged to Single_copy, Multi_copy or Unique.

Table S1: Statistics of the completeness of the genome based on BUSCO benchmark

Table S2: Statistics of gene structure annotations.

Table S3: Statistics of repeat elements in forest musk deer genome annotated by RepeatMasker

Table S4: Statistics of SSRs in the forest musk deer genome.

Table S5: Summary of Orthologous genes in forest musk deer and other ten animals. “Single_copy” included the common orthologs with the same number of copies in different species; “Multi_copy” included the common orthologs with different copy numbers in different species; “Unique” included the orthologs that were only in one species; “Unclustered gene” included the genes that could not be clustered into known gene families; “Other” included the genes that could be clustered into known gene families, but were not belonged to the above categories.

## Abbreviations

BBH: bidirectional best hit; BI: BUSCO: Benchmarking Universal Single-Copy Orthologs; GO: Gene Ontology; KASS: KEGG Automatic Annotation Server; NCBI: National Center for Biotechnology Information; nr, nonredundant; OR: olfactory receptor; PE: RNA-seq: RNA sequencing; SSR: simple sequence repeat; TE: transposable elements; WEGO: Web Gene Ontology Annotation Plot

## Funding

This work was supported by National Key Program of Research and Development, Ministry of Science and Technology (2016YFC0503200), and National Natural Science Foundation of China (NSFC31702032)

## Competing interests

The authors declare that they have no competing interests.

## Author contributions

Z.F., X.Z., J.L., W.Q. and B.Y. designed and supervised the project. Z.F., W.L., C.Y., J.J., C.P., J.Y., P.B., Y.S., and K.C. performed the bioinformatics analyses. M.P. revised the manuscript. Z.F. and B.Y. wrote the manuscript.

## Supplementary Material

GIGA-D-17-00185_Original_Submission.pdf

GIGA-D-17-00185_Revision_1.pdf

GIGA-D-17-00185_Revision_2.pdf

Response_to_Reviewer_Comments_Original_Submission.pdf

Response_to_Reviewer_Comments_Revision_1.pdf

Reviewer_1_Report_(Original_Submission) -- Aaron Shafer 9/13/2017 Reviewed

Reviewer_1_Report_(Revision_1) -- Aaron Shafer 1/27/2018 Reviewed

Reviewer_2_Report_(Original_Submission) -- Sylvain Foissac 9/19/2017 Reviewed

Reviewer_2_Report_(Revision_1) -- Sylvain Foissac 1/22/2018 Reviewed

Supplemental material
