# Supplementary material for: The draft genome sequence of forest musk deer (Moschus berezovskii)
Source: Gigascience. 2018 Apr 9;7(4):giy038. doi: 10.1093/gigascience/giy038 (PMC5906906; doi:10.1093/gigascience/giy038)
Supplement: GIGA-D-17-00185_Original_Submission.pdf [file giy038_giga-d-17-00185_original_submission.pdf]

GigaScience

The draft genome sequence of forest musk deer (Moschus berezovskii)

--Manuscript Draft--

**Manuscript Number:**	GIGA-D-17-00185
**Full Title:**	The draft genome sequence of forest musk deer (Moschus berezovskii)
**Article Type:**	Data Note
**Funding Information:**	National Key Program of Research and Development, Ministry of Science and Technology (2016YFC0503200) — Dr. Bisong Yue
**Abstract:**	Background: The forest musk deer, Moschus berezovskii, is one of seven musk deer (Moschus spp.) and is distributed in Southwest China. Akin to other musk deer, the forest musk deer has been traditionally, and is currently, hunted for its musk (i.e. global perfume industry). Considerable hunting pressure and habitat loss has caused significant population declines and therefore the Chinese government commenced captive breeding programs for musk harvesting in the 1950s. However, the prevalence of fatal diseases is considerably restricting population increases. Disease severity and extent is exacerbated by inbreeding and genetic diversity declines in captive musk deer populations. It is essential for the physical and genetic health of captive and wild forest musk deer populations to improve knowledge of its immune system and genome. We have thus sequenced the whole genome of the forest musk deer, completed the genomic assembly and annotation, and performed preliminary bioinformatic analyses.<br>Findings: A total of 407 Gb raw reads from whole-genome sequencing was generated by the Illumina Hiseq4000 platform. The final assembly genome is around 2.72 Gb, with a contig N50 length of 22.6 kb and a scaffold N50 length 2.85 Mb. We identified 24,352 genes, and found 42.05% of the genome is composed of repetitive elements. We also detected 1,236 olfactory receptor genes. The genome-wide phylogenetic tree indicated that the forest musk deer was within the order Artiodactyla, and it appeared as the sister clade of four members of family Bovidae. In total, 576 genes were under positive selection in the forest musk deer lineage.<br>Conclusions: We provide the first genome sequence and gene annotation for the forest musk deer. The availability of these resources will be very useful for the conservation and captive breeding for this Endangered and economically important species, and for reconstructing the evolutionary history of the order Artiodactyla.
**Corresponding Author:**	Zhenxin Fan, Ph.D.<br>Sichuan University<br>Chengdu, Sichuan CHINA
**Corresponding Author Secondary Information:**	
**Corresponding Author's Institution:**	Sichuan University
**Corresponding Author's Secondary Institution:**	
**First Author:**	Zhenxin Fan, Ph.D.
**First Author Secondary Information:**	
**Order of Authors:**	Zhenxin Fan, Ph.D.
	Bisong Yue
	Wujiao Li
	Chaochao Yan
	Jing Li
	Yongmei Shen
**Order of Authors Secondary Information:**	

Powered by Editorial Manager® and ProduXion Manager® from Aries Systems Corporation

**Opposed Reviewers:**	
**Additional Information:**	
**Question**	**Response**
Are you submitting this manuscript to a special series or article collection?	No
**Experimental design and statistics**<br><br>Full details of the experimental design and statistical methods used should be given in the Methods section, as detailed in our Minimum Standards Reporting Checklist. Information essential to interpreting the data presented should be made available in the figure legends.<br><br>Have you included all the information requested in your manuscript?	Yes
**Resources**<br><br>A description of all resources used, including antibodies, cell lines, animals and software tools, with enough information to allow them to be uniquely identified, should be included in the Methods section. Authors are strongly encouraged to cite Research Resource Identifiers (RRIDs) for antibodies, model organisms and tools, where possible.<br><br>Have you included the information requested as detailed in our Minimum Standards Reporting Checklist?	Yes
**Availability of data and materials**<br><br>All datasets and code on which the conclusions of the paper rely must be either included in your submission or deposited in publicly available repositories (where available and ethically appropriate), referencing such data using a unique identifier in the references and in the "Availability of Data and Materials" section of your manuscript.<br><br>Have you have met the above requirement as detailed in our Minimum Standards Reporting Checklist?	Yes

Powered by Editorial Manager® and ProduXion Manager® from Aries Systems Corporation

Manuscript

Click here to download Manuscript MuskDeer_dataNote_GSv1_new.docx

The draft genome sequence of forest musk deer (*Moschus berezovskii*)

Zhenxin Fan[1,†], Wujiao Li[1,†], Wenhua Qi[2,†], Jiazheng Jin[1], Kai Cui[1], Chaochao Yan[1], Changjun Peng[1], Zuoyi Jian[1], Megan Price[1], Xiuyue Zhang[1], Yongmei Shen[3], Jing Li[1,*], Bisong Yue[1,*]

[1] Key Laboratory of Bioresources and Ecoenvironment (Ministry of Education), College of Life Sciences, Sichuan University, Chengdu 610064, People's Republic of China

[2] College of Life Science and Engineering, Chongqing Three Gorges University, Chongqing 404100, People's Republic of China

[3] Sichuan Engineering Research Center for Medicinal Animals, Xichang 615000, People's Republic of China

† Contributed equally to this work

* **Corresponding author:** Bisong Yue (bsyue@scu.edu.cn), and Jing Li (ljtjf@126.com)

Emails: zxfan@scu.edu.cn (Zhenxin Fan); hnnd059@gmail.com (Wujiao Li); wenhuaqi357@163.com (Wenhua Qi); jinjiazhengxiao@163.com (Jiazheng Jin); 2015222040118@stu.scu.edu.cn (Kai Cui); yccvican@gmail.com (Chaochao Yan); jj-5380682@163.com (Changjun Peng); jzuoyi@126.com (Zuoyi Jian); meganprice@scu.edu.cn (Megan Price); zhangxy317@126.com (Xiuyue Zhang); Yongmei Shen (810316122@qq.com); Jing Li (ljtjf@126.com); bsyue@scu.edu.cn (Bisong Yue)

Abstract

Background: The forest musk deer, *Moschus berezovskii*, is one of seven musk deer (*Moschus* spp.) and is distributed in Southwest China. Akin to other musk deer, the forest musk deer has been traditionally, and is currently, hunted for its musk (i.e. global perfume industry). Considerable hunting pressure and habitat loss has caused significant population declines and therefore the Chinese government commenced captive breeding programs for musk harvesting in the 1950s. However, the prevalence of fatal diseases is considerably restricting population increases. Disease severity and extent is exacerbated by inbreeding and genetic diversity declines in captive musk deer populations. It is essential for the physical and genetic health of captive and wild forest musk deer populations to improve knowledge of its immune system and genome. We have thus sequenced the whole genome of the forest musk deer, completed the genomic assembly and annotation, and performed preliminary bioinformatic analyses.

Findings: A total of 407 Gb raw reads from whole-genome sequencing was generated by the Illumina Hiseq4000 platform. The final assembly genome is around 2.72 Gb, with a contig N50 length of 22.6 kb and a scaffold N50 length 2.85 Mb. We identified 24,352 genes, and found 42.05% of the genome is composed of repetitive elements. We also detected 1,236 olfactory receptor genes. The genome-wide phylogenetic tree indicated that the forest musk deer was within the order Artiodactyla, and it appeared as the sister clade of four members of family Bovidae. In total, 576 genes were under positive selection in the forest musk deer lineage.

Conclusions: We provide the first genome sequence and gene annotation for the forest musk deer. The availability of these resources will be very useful for the conservation and captive breeding for this Endangered and economically important species, and for reconstructing the evolutionary history of the order Artiodactyla.

Keywords: Forest musk deer; whole genome sequencing; genome assembly; annotation; phylogeny

Data Description

1) Background

The seven musk deer species of genus *Moschus* are endemic to Asia, are currently listed under Appendix II in CITES and are listed under Category I of the State Key Protected Wildlife List of China [1-3]. All musk deer species are considered as globally threatened, with six being listed as Endangered and one as Vulnerable by the IUCN [4]. *Moschus* is the only extant genus of Moschidae and musk deer are considered as primitive deer. The genus of musk deer is characterized by the musk secreted by the scent glands of adult males [5]. The forest musk deer (*Moschus berezovskii*) is one of the five recognized musk deer species of China and have historically been distributed in Southwest China [6,7]. The forest musk deer has been listed as globally endangered, as Critically Endangered on the 2015 China Red List, and is also on the State Key Protected Wildlife List of China [4].

Musk deer have been hunted for thousands of years, as the musk has been widely used in traditional Chinese medicines. In the last two centuries, hunting of all musk deer species significantly increased for the global trade of the commercially valuable musk secretion as an essential basis for perfume manufacture [5]. Since the 1950s, populations of forest musk deer have declined dramatically from poaching of deer for the musk pods (i.e. entire gland) and significant habitat destruction [3,6,8]. As a consequence, the Chinese government has encouraged musk-using enterprises to participate in artificial breeding programs since the early 1950s [9]. The musk can be collected from

male musk deer in these captive populations without harvesting individuals, further enhancing the commercial and conservation value of captive populations.

The captive population of the forest musk deer is the largest among all the musk deer species [2,10]. The Miyaluo farming population in Sichuan Province (China) was one of the earliest established captive breeding populations. This population had grown rapidly to approximately 400 in 2010 [10]. However, the prevalence of fatal diseases is considerably restricting population increases [11]. Common diseases of forest musk deer in the Miyaluo population are dyspepsia, pneumonia, metritis, urinary stones and abscesses, with abscesses being one of the most prevalent causes of death [7]. Disease severity and extent is exacerbated by inbreeding and genetic diversity declines in this and other captive musk deer populations.

It is essential for the physical and genetic health of captive and wild forest musk deer populations to improve knowledge of its immune system and genome. We have thus sequenced the whole genome of the forest musk deer, subsequently completed the genomic assembly and annotation, and performed preliminary bioinformatic analyses, such as phylogenetic tree, selection and gene enrichments.

2) Sample information and sequencing

The thigh muscle sample was collected from a Miyaluo male forest musk deer that naturally died (Sichuan Province, China) in 2015. We constructed six different insert size libraries: 230bp, 500bp, 2kb, 5kb, 10kb, and 15kb. These libraries were sequenced by Illumina Hiseq 4000 platform at Novogene (Beijing,

China). A total of 407Gb of raw data were generated, after filtering out low quality, duplicate and adaptor polluted reads. Approximately 360Gb of high-quality reads were retained for genome assembly (Table 1).

3) Genome assembly and evaluation

We performed k-mer (17-mer) analysis by short insert size library reads before assembly, and the forest musk deer genome size was estimated to be 2.95Gb (Figure S1). The assembly was first analyzed by SOAPdenovo2 [12] with the parameters set as "all -d 2 –M 2 –k 35". Intra-scaffold gaps were then filled using Gapcloser with reads from 230bp and 500bp libraries, while SSPACE [13] was used to build super-scaffolds. After scaffolding by SSPACE, we used Gapcloser again to fill any gaps. Subsequently, we obtained the forest musk deer genome with a size of 2.72Gb (all the sequences with length shorter than 300bp were removed). The N50s of contigs and scaffolds of forest musk deer genome is 22.6kb and 2.85Mb, respectively (Table 2). The forest musk deer genome is slightly longer than the genomes of closely related species, such as reindeer (*Rangifer tarandus*), yak (*Bos grunniens*) and wisent (*Bison bonasus*) genomes.

We used Cegma (v2.5) [14] and BUSCO (3.0) to evaluate the genome complement. Cegma results showed that 91.53% complete and 95.97% partial gene set could be found in this assembly genome (Table S1), and BUSCO results showed that 84.5% of the eukaryotic single-copy genes were captured (Table S2). Furthermore, we downloaded musk gland RNA-seq data (SRA accession: SRR2098995, SRR2098996) of forest musk deer from NCBI to evaluate the assembly. We found that almost all reads (99.3%) could be aligned to forest

musk deer genome by Bowtie2 [15]. These results showed our forest musk deer genome was of high quality, and was suitable as a reference genome for the family Moschidae.

4) Annotation

We combined the *de novo*, homology-based and transcriptome-based prediction to identify protein-coding genes in the forest musk deer genome. The software Augustus (version 3.2.1) [16] was used for *de novo* prediction based on the parameter trained for forest musk deer. For homology prediction, protein sequences from four mammals (human, pig, sheep and cattle) were analyzed with TBLASTN (BLAST version 2.2.26+) against forest musk deer genome. Potential gene regions were identified by software solar, the coding sequence region were further identified by GeneWise (version 2.4.1) [17]. For transcriptome-based prediction, musk gland RNA-seq data were assembled by Trinity with genome guide and *de novo* mode, respectively. The gene structures were obtained by PASA pipeline (version 2.0.2) [18]. We used EVM to integrate the above evidence and obtained a consensus gene set. We used Apollo to manually inspect gene structure in scaffolds of sizes above 1Mb to gain a more accurate gene structure. We consequently found a total of 24,352 genes predicted to be present in the forest musk deer genome.

Functional annotation of forest musk deer genes was undertaken based on the best match derived from the alignments to proteins annotated in Swiss-Prot and TrEMBL databases [19]. Functional annotation used BlastP tools with the same E-value cut-off of 1E-5. We also annotated proteins against the NCBI

non-redundant (nr) protein database. The outputs of blast searching against the NCBI nr protein database were imported into BLAST2GO (B2G4PIPE v2.5) for Gene Ontology (GO) [20] term mapping. Term mapping used annotated motifs and domains using InterProScan (interproscan-5.18-57.0) [21] by searching against publicly available databases. To find the best match for each gene, KEGG pathway maps were used by searching KEGG databases [22] through the KEGG Automatic Annotation Server (KAAS) using the bi-directional best hit (BBH) method. In total, 23,023 out of 24,352 (94.5%) protein-coding genes were searched within the publicly available functional databases of TrEMBL, Swiss-Prot, Interpro, GO and KEGG. Of which, 22,696 (93.20% TrEMBL), 18,771 (77.08% Swiss-Prot), 22,221 (91.12% Interpro), 15,736 (64.62% GO) and 10,846 (44.54% KEGG) genes showed significant similarity matches (Figure 1; Table 3). The functional comparisons with two closely related species (cattle and sheep) for GO classification were submitted to the WEGO [23] (Figure S2).

5) Repetitive sequences and transposable elements

Transposable elements (TEs) and other repeats make up a substantial fraction of mammalian genomes and contribute to gene and/or genome evolution [24]. The TE content, type, copy number, subfamily, and divergence rate were investigated in the forest musk deer genome based on two strategies: the library based strategy of RepeatMasker [25] and the *de novo* based strategy of RepeatScout [26]. The forest musk deer genome has large numbers of TEs, comprising 42.05% of the genome (Table S3), which is similar to those of cattle (46.5%) [24] and goats (42.2%) [27]. We grouped the four different types of TEs in the forest

musk deer genome, which were DNA transposons, LTR, LINE, and SINE retrotransposons. The LINEs were the most common repeats in forest musk deer genome; followed by SINEs > LTR > DNA. We also analyzed the degree of divergence for each type of TE in the forest musk deer genome. We found there was a recent burst activity involving LINE transposons and a second, older burst activity of LTR and DNA transposons (Figure S3).

A total of 542,135 microsatellites (simple sequence repeats, SSRs) were identified by software MSDB [28] in the forest musk deer genome assembly (Table S4), which accounted for 0.45% of its whole genome length. Mononucleotide SSRs were the most abundant category, accounting for 41.75% of all of the SSRs; followed by followed by: di- > tri- > tetra- > penta- > hexa nucleotide SSRs (Table S4).

6) Gene families

To estimate species-specific and shared genes in the forest musk deer in comparison to ten mammal species, we used orthoMCL [29] to define the orthologous genes. We downloaded the genomes and gene annotations of the ten additional species (human, horse, dog, cattle, mouse, yak, sheep, Tibetan antelope, alpaca, and pig) from Ensembl [30] or NCBI (Table S5). In total, we identified 18,855 homologous gene families shared by forest musk deer and the ten additional species, 221 gene families that were specific to forest musk deer, and 2,003 gene families found in the ten additional species but not in the forest musk deer (Table S6; Figure S4). In addition, we found 5,372 one-to-one

orthologous genes within forest musk deer and the ten species (Table S5), which was used in phylogenetic analyses.

7) Olfactory receptor genes

Olfactory receptor (OR) genes form the largest gene family in mammalian genomes [31]. We detected OR genes in the forest musk deer genome by orfam (https://github.com/jianzuoyi/orfam), which was developed by our laboratory. In total, we identified 1,236 OR genes, which included 866 intact, 266 pseduogenes, and 104 truncted genes. The number of OR genes in forest musk deer was larger than described in primates (i.e. human, macaque, chimpanzee, and marmoset) (Table S7), where previous research found that there was degeneration of OR genes in primates [32-36].

8) Phylogenetic analysis

We constructed the phylogenetic trees based on Bayesian inference (BI) [37] and maximum likelihood (ML) [38,39] analyses with the discovered 5,372 one-to-one orthologous genes (Supplementary methods). All the different methods generated the same topology and obtained the well-supported phylogenetic tree (Figure 2). The forest musk deer was within the suborder Ruminantia, order Artiodactyla, and it appeared as the sister clade of four members of family Bovidae (sheep, yak, cattle, and Tibetan antelope; Figure 2). Since we do not have high quality genome sequences for species within family Cervidae, the relationship between Moschidae, Cervidae, and Bovidae at the genomic level is tentative and needs further investigation.

9) Selection and gene enrichment

We applied the branch-site likelihood ratio test at PAML [40] to detect the positively selected genes in the forest musk deer compared with other ten species in Table S5. We identified 576 positively selected genes (Table S8). The gene enrichment analyses found that these genes were of particular interest, such as primary immunodeficiency and DNA binding (Table S9 and S10).

Conclusions

Here, we report the first draft genome assembly of the forest musk deer genome, a species that is of particular importance to China's ecology, biodiversity conservation, economy, and medicine. The availability of the genome and these results will be very useful for the conservation and captive breeding of this Endangered and economically important species, and for reconstructing the evolutionary history of the order Artiodactyla.

Funding

This work was supported by National Key Program of Research and Development, Ministry of Science and Technology (2016YFC0503200).

Availability of supporting data

The DNA sequencing data have been deposited into the NCBI Sequence Read Archive (SRA) under the ID PRJNA317652.

Conflicts of interest

The authors declare that they have no competing interests.

Author's contributions

Z.F., X.Z., J.L., and B.Y. designed and supervised the project. Z.F., W.L., C.Y., J.J., C.P., J.Y., Y.S., and K.C. performed the bioinformatics analyses. M.P. revised the manuscript. Z.F. and B.Y. wrote the manuscript.

Figure Legend

Figure 1 Functional annotation statistics. Venn diagram illustrating distribution of high-score matches of the functional annotation in forest musk deer genome from five public databases.

Figure 2 Genome wide phylogenetic trees. We constructed the phylogenetic trees based on Bayesian inference and maximum likelihood analyses with 5,372 one-to-one orthologous genes between the forest musk deer and ten other species.

Figure S1 K-mer (k=17) distributions in forest musk deer genome.

Figure S2 GO comparative analysis and functional classification between forest musk deer, sheep and cattle.

Figure S3 Distribution of divergence of each type of TEs in the forest musk deer genome. The divergence rate was calculated between the identified TE elements in the genome and the consensus sequence in the TE library used. SINEs: Short interspersed elements, LINEs: Long interspersed elements, LTR: Long terminal repeat retrotransposon.

Figure S4 Protein orthology comparison between different genomes. There were forest musk deer (*Moschus bweezovskii*), cattle (*Bos taurus*), yak (*Bos grunniens*), sheep (*Ovis aries*), Tibetan antelope (*Pantholops hodgsonii*), alpaca (*Vicugna pacos*), and pig (*Sus scrofa*), which representing Artiodactyla; human (*Homo sapiens*, Primates), horse (*Equus caballus*, Perissodactyla), and dog (*Canis lupus familiaris*, Carnivora), mouse (*Mus musculus*, Rodentia). For each animal,

proteins are represented by bars and classified according to orthoMCL analysis: Single_copy (Oliver) include the common orthologs with the same number of copies in different species; Multi_copy (Red) include the common orthologs with different copy numbers in the different species; Unique (Magenta) include the orthologs just in one species; Unclustered gene (Yelollow) include the genes that cannot be clustered into known gene families; Other (Blue) include the genes that can be clustered into known gene families, but it not belongs to Single, Multi or Unique.

References

1. Cobert GB, Hill JE. The mammals of the Indomalaysia region: a systematic review. London (UK): Natural History Museum Publications Oxford University Press. 1992.

2. Wu J, Wang W. The Musk Deer of China. The China Forestry Publishing House, Beijing. 2006.

3. Sheng H, Liu Z. The Musk Deer in China. The Shanghai Scientific & Technical Publishers, Shanghai. 2007.

4. IUCN. The IUCN Red List of Threatened Species. 2017 (http://www.iucnredlist.org/)

5. Green MJB. The distribution, status and conservation of the Himalayan musk deer (Moschus chrysogaster). Biol. Conserv. 1986;35, 347–75.

6. Sheng H. Genus Moschus in China. In: Wang, S. (Ed.), China Red Data Book of Endangered Animals. Science Press, Beijing. 1998.

7. Zhao K, Liu Y, Zhang X, et al. Detection and characterization of antibiotic-resistance genes in Arcanobacterium pyogenes strains from abscesses of forest musk deer. J Med Microbiol. 2011;60:1820-6.

8. Yang Q, Meng X, Xia L, Feng Z. Conservation status and causes of decline of musk deer (Moschus spp.) in China. Biol. Conserv. 2003;109, 333–342.

9. Peng H, Liu S, Zou F, Zeng B, Yue B. Genetic diversity of captive forest musk deer (Moschus berezovskii) inferred from the mitochondrial DNA control region. Anim Genet. 2009;40(1):65-72.

10. Huang J, Li Y, Li P, et al. Genetic quality of the Miyaluo captive forest musk deer (Moschus berezovskii) population as assessed by microsatellite loci. Biochemical Systematics & Ecology, 2013;47(8):25-30.

11. Lu X, Qiao J, Wu X, Su L. A review of mainly affected on musk-deer diseases: purulent, respiratory system and parasitic diseases. J Economic Anim 2009, 13, 104–107.

12. Luo R, Liu B, Xie Y, et al. SOAPdenovo2: an empirically improved memory-efficient short-read de novo assembler. Gigascience 2012;1(1):18.

13. Boetzer M, Henkel CV, Jansen HJ, Butler D, Pirovano W. Scaffolding pre-assembled contigs using SSPACE. Bioinformatics. 2011;27(4):578-9.

14. Parra G, Bradnam K, Korf I. CEGMA: a pipeline to accurately annotate core genes in eukaryotic genomes. Bioinformatics. 2007;23(9):1061-7.

15. Langmead B, Salzberg SL. Fast gapped-read alignment with Bowtie 2. Nat Methods. 2012;9(4):357-9.

16. Stanke M, Diekhans M, Baertsch R, Haussler D. Using native and syntenically mapped cDNA alignments to improve de novo gene finding. Bioinformatics. 2008;24(5):637-44.

17. Birney E, Clamp M, Durbin R. GeneWise and Genomewise. Genome Res. 2004;14(5):988-95.

18. Haas BJ, Delcher AL, Mount SM, et al. Improving the Arabidopsis genome annotation using maximal transcript alignment assemblies. Nucleic Acids Res. 2003;31(19):5654-66.

19. Boeckmann B, Bairoch A, Apweiler R, et al. The SWISS-PROT protein knowledgebase and its supplement TrEMBL in 2003. Nucleic Acids Res. 2003;31(1):365-70.

20. Gene Ontology Consortium. Gene Ontology annotations and resources. Nucleic Acids Res. 2013;41(Database issue):D530-5.

21. Hunter S, Apweiler R, Attwood TK, et al. InterPro: the integrative protein signature database. Nucleic Acids Res. 2009;37(Database issue):D211-5.

22. Kanehisa M, Goto S. KEGG: kyoto encyclopedia of genes and genomes. Nucleic Acids Res. 2000;28(1):27-30.

23. Ye J, Fang L, Zheng H, et al. WEGO: a web tool for plotting GO annotations. Nucleic Acids Res. 2006;34(Web Server issue):W293-7.

24. Adelson DL, Raison JM, Edgar RC. Characterization and distribution of retrotransposons and simple sequence repeats in the bovine genome. Proc Natl Acad Sci U S A. 2009;106(31):12855-60.

25. Smit AFA, Hubley R, Green P. 2016. RepeatMasker website and server[CP/OL]. (2016-9-12)[2016-10-15]. http://www.repeatmasker.org/.

26. Price AL, Jones NC, Pevzner PA. De novo identification of repeat families in large genomes. Bioinformatics. 2005;21 Suppl 1:i351-8.

27. Dong Y, Xie M, Jiang Y, et al. Sequencing and automated whole-genome optical mapping of the genome of a domestic goat (Capra hircus). Nat Biotechnol. 2013;31(2):135-41.

28. Du L, Li Y, Zhang X, Yue B. MSDB: a user-friendly program for reporting distribution and building databases of microsatellites from genome sequences. J Hered. 2013;104(1):154-7.

29. Li L, Stoeckert CJ Jr, Roos DS. OrthoMCL: identification of ortholog groups for eukaryotic genomes. Genome Res. 2003;13(9):2178-89.

30. Yates A, Akanni W, Amode MR et al. Ensembl 2016. Nucleic Acids Res. 2016;44(D1):D710–16.

31. Firestein S. How the olfactory system makes sense of scents. Nature. 2001;413(6852):211-8.

32. Niimura Y, Nei M. Extensive gains and losses of olfactory receptor genes in mammalian evolution. PLoS One. 2007;2(8):e708.

33. Go Y, Niimura Y. Similar numbers but different repertoires of olfactory receptor genes in humans and chimpanzees. Mol Biol Evol. 2008;25(9):1897-907.

34. Matsui A, Go Y, Niimura Y. Degeneration of olfactory receptor gene repertories in primates: no direct link to full trichromatic vision. Mol Biol Evol. 2010;27(5):1192-200.

35. Lee K, Nguyen DT, Choi M, et al. Analysis of cattle olfactory subgenome: the first detail study on the characteristics of the complete olfactory receptor repertoire of a ruminant. BMC Genomics. 2013;14:596.

36. Niimura Y, Matsui A, Touhara K. Extreme expansion of the olfactory receptor gene repertoire in African elephants and evolutionary dynamics of

orthologous gene groups in 13 placental mammals. Genome Res. 2014;24(9):1485-96.

37. Ronquist F, Huelsenbeck JP. MrBayes 3: Bayesian phylogenetic inference under mixed models. Bioinformatics. 2003;19(12):1572-4.

38. Guindon S, Dufayard JF, Lefort V, et al. New algorithms and methods to estimate maximum-likelihood phylogenies: assessing the performance of PhyML 3.0. Syst Biol. 2010;59(3):307-21.

39. Stamatakis A, Hoover P, Rougemont J. A rapid bootstrap algorithm for the RAxML Web servers. Syst Biol. 2008;57(5):758-71.

40. Yang Z. PAML 4: phylogenetic analysis by maximum likeli- hood. Mol Biol Evolution. 2007;24(8):1586–91.

 Click here to download Table Table 1_sequencing.docx

Table 1 Genome sequencing information.

Insert size (bp)	Read length (bp)	Raw data		Clean data	
		Total bases (Gb)	Sequencing depth (x)	Total bases (Gb)	Sequencing depth (x)
230	125	135.76	46.02	125.96	42.70
500	125	102.51	34.75	88.52	30.01
2,000	125	59.0	20.00	50.16	17.00
5,000	125	51.57	17.48	46.39	15.73
10,000	125	28.16	9.55	24.67	8.36
15,000	125	30.34	10.28	28.14	9.54
Total		407.34	138.08	363.84	123.34

Note: Genome size is 2.95Gb.

Click here to download Table Table 2_assembly.docx

Table 2 statistics of the final assembly of forest musk deer genome.

Genome assembly	Length
Contig N50 (kb)	22.6
Scaffold N50 (Mb)	2.85
Total length (Gb)	2.72

Table 3 Click here to download Table Table 3 _FunctionalAnnotation.docx

Table 3 Functional annotation statistics of forest musk deer genome by various methods

	Database	Number	Percent (%)
Total		24,352	100.00
	Swissprot	18,771	77.08
	TrEMBL	22,696	93.20
Annotated	KEGG	10,846	44.54
	Interpro	22,221	91.12
	GO (blast2go)	15,736	64.62
	GO (Interproscan)	14,815	60.84
Un-annotated		1,329	5.77

Figure 1

Click here to download Figure Figure 1_annotation.tif

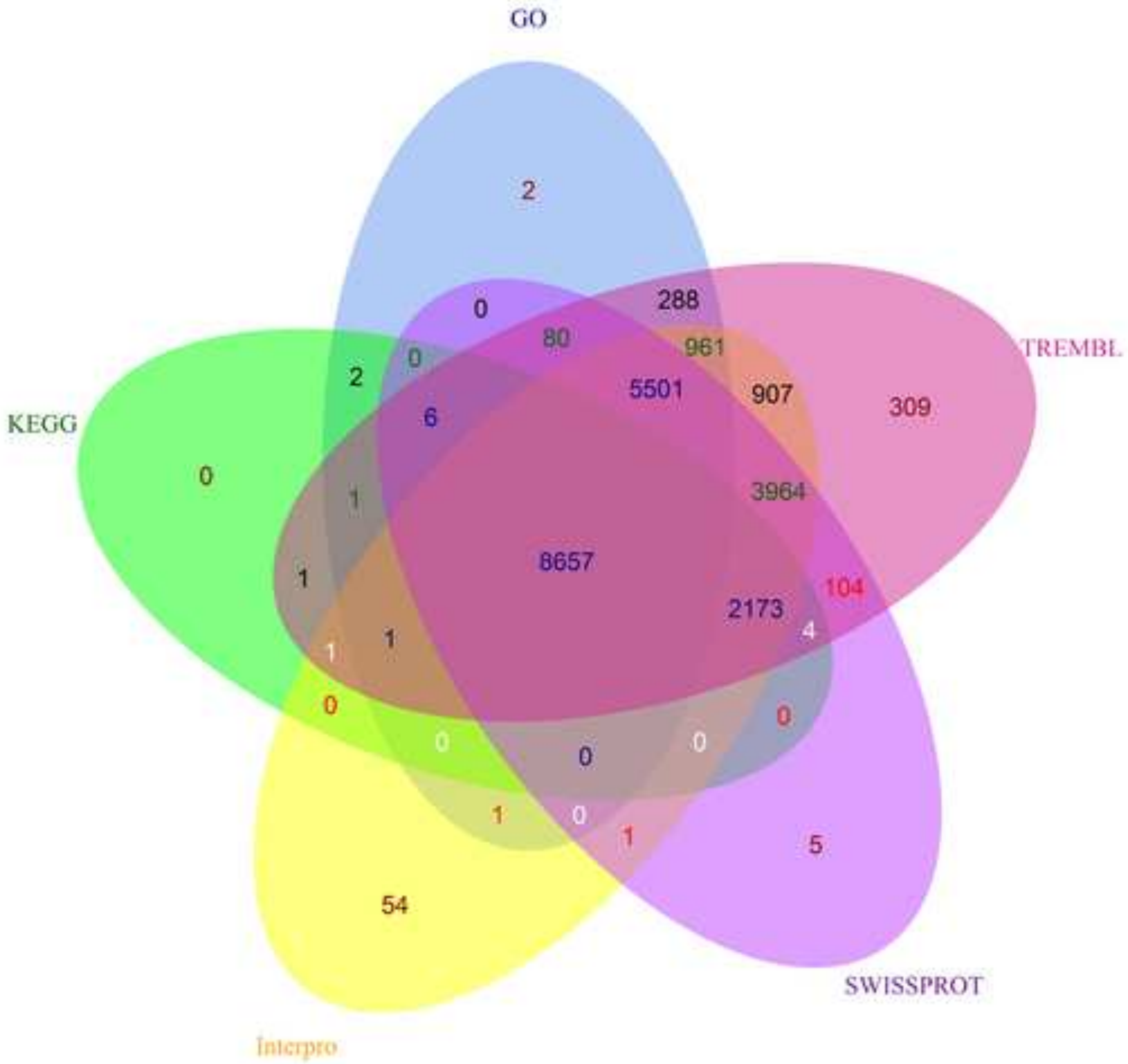

Figure 2

Click here to download Figure Figure 2_tree.tif

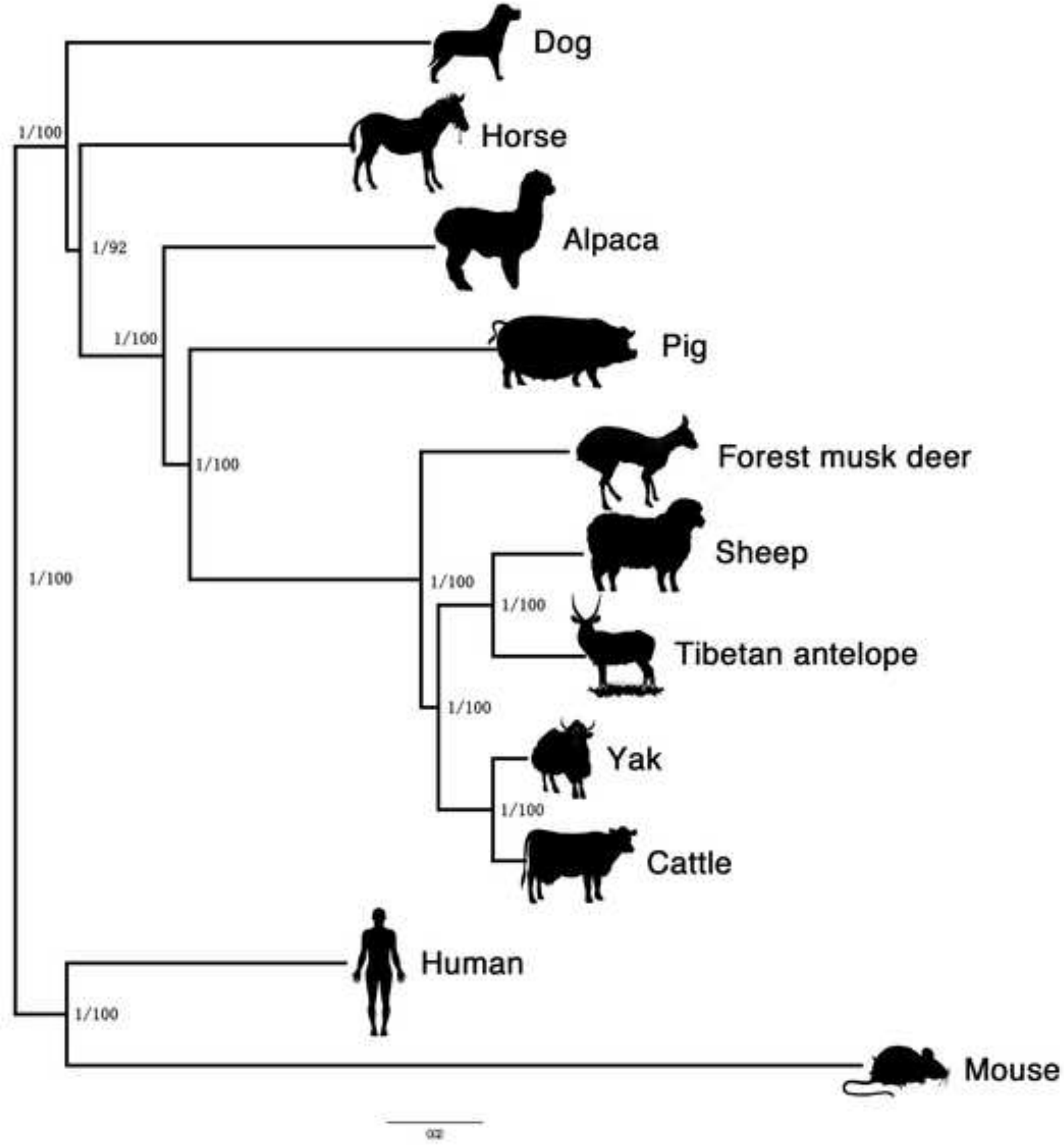

Supplementary Figure 1

Click here to access/download
Supplementary Material
Figure S1_kmer.tif

Supplementary Figure 2

Click here to access/download
Supplementary Material
Figure S2_wego.pdf

Supplementary Figure 3

Click here to access/download
Supplementary Material
Figure S3_TE_type.tif

Supplementary Figure 4

Click here to access/download
Supplementary Material
Figure S4_OrthologousGene.tif

Supplementary methods

Click here to access/download
Supplementary Material
SupplementaryMethods.docx

Supplementary Tables

Click here to access/download
Supplementary Material
SI_Tables_new.docx
