# Supplementary material for: The draft genome sequence of forest musk deer (Moschus berezovskii)
Source: Gigascience. 2018 Apr 9;7(4):giy038. doi: 10.1093/gigascience/giy038 (PMC5906906; doi:10.1093/gigascience/giy038)
Supplement: GIGA-D-17-00185_Revision_1.pdf [file giy038_giga-d-17-00185_revision_1.pdf]

GigaScience

The draft genome sequence of forest musk deer (Moschus berezovskii)

--Manuscript Draft--

**Manuscript Number:**	GIGA-D-17-00185R1
**Full Title:**	The draft genome sequence of forest musk deer (Moschus berezovskii)
**Article Type:**	Data Note
**Funding Information:**	National Key Program of Research and Development, Ministry of Science and Technology (2016YFC0503200) — Dr. Bisong Yue<br>National Natural Science Foundation of China (31702032) — Dr. Wenhua Qi
**Abstract:**	Background: The forest musk deer, Moschus berezovskii, is one of seven musk deer (Moschus spp.) and is distributed in Southwest China. Akin to other musk deer, the forest musk deer has been traditionally, and is currently, hunted for its musk (i.e. global perfume industry). Considerable hunting pressure and habitat loss has caused significant population declines and therefore the Chinese government commenced captive breeding programs for musk harvesting in the 1950s. However, the prevalence of fatal diseases is considerably restricting population increases. Disease severity and extent is exacerbated by inbreeding and genetic diversity declines in captive musk deer populations. It is essential for the physical and genetic health of captive and wild forest musk deer populations to improve knowledge of its immune system and genome. We have thus sequenced the whole genome of the forest musk deer, completed the genomic assembly and annotation, and performed preliminary bioinformatic analyses.<br>Findings: A total of 407 Gb raw reads from whole-genome sequencing was generated by the Illumina Hiseq4000 platform. The final assembly genome is around 2.72 Gb, with a contig N50 length of 22.6 kb and a scaffold N50 length 2.85 Mb. We identified 24,352 genes, and found 42.05% of the genome is composed of repetitive elements. We also detected 1,236 olfactory receptor genes. The genome-wide phylogenetic tree indicated that the forest musk deer was within the order Artiodactyla, and it appeared as the sister clade of four members of family Bovidae. In total, 576 genes were under positive selection in the forest musk deer lineage.<br>Conclusions: We provide the first genome sequence and gene annotation for the forest musk deer. The availability of these resources will be very useful for the conservation and captive breeding for this Endangered and economically important species, and for reconstructing the evolutionary history of the order Artiodactyla.
**Corresponding Author:**	Zhenxin Fan, Ph.D.<br>Sichuan University<br>Chengdu, Sichuan CHINA
**Corresponding Author Secondary Information:**	
**Corresponding Author's Institution:**	Sichuan University
**Corresponding Author's Secondary Institution:**	
**First Author:**	Zhenxin Fan, Ph.D.
**First Author Secondary Information:**	
**Order of Authors:**	Zhenxin Fan, Ph.D.
	Bisong Yue
	Wujiao Li
	Chaochao Yan
	Jing Li

Powered by Editorial Manager® and ProduXion Manager® from Aries Systems Corporation

	Yongmei Shen
	Wenhua Qi
**Order of Authors Secondary Information:**	
**Response to Reviewers:**	Response to the comments<br><br>Reviewer #1<br>Overall the genome reflects the industry standard for sequencing and assembly. The paper reads as a series of independent analyses that have not been integrated or justified. Some add virtually nothing to the paper, while others need to be flushed out a bit further (i.e. more detail and justification). If there is a word constraint I have highlighted two sections that could be dropped thereby providing room to expand on other sections. The english is generally good but a careful proof by a native speaker would be useful.<br><br>1. Please provide the genome statistics after the short-read assembly, as this is useful information to the reader.<br>Response: We added the information into the manuscript (Lines 125 - 132) and Table 2.<br><br>2. Statements about length relative to other ungulates should be avoided, as this is dependent on the assembly quality and strategy. I do not find it biologically meaningful.<br>Response: We deleted them.<br><br>3. The TE analysis needs to be rethought; simply identifying repeats is not equivalent to identifying TEs. There is a severe lack of information provided here and it needs to be flushed out or dropped. How were the four types defined for exampled?<br>Response: We explained within the manuscript (Lines 218 - 232).<br><br>4. The Olfactory gene detection is not justified and it's not clear why this warrants it's own section.<br>Response: We combined the olfactory receptor genes section into the “Gene families” section, and simplified it (Lines 259 - 263).<br><br>5. The phylogenetic analysis does not add anything given there were no cervid genomes included; what was the point of this?<br>Response: Because there are no Cervidae genomes, we could not fully address the phylogenetic position of forest musk deer at the complete genome sequences level in our manuscript. However, the phylogenetic tree still gave us the general picture of the phylogenetic relationship between forest musk deer and its related species, which their genomes are available now, and the results also indicated that we should add the genome sequences from family Cervidae in the further investigation. Therefore, we still keep the phylogenetic analysis in the manuscript.<br><br>6. Again, no closely related species are included in the selection analysis. What was the point of this? Would you expect 500 genes to deviate from a closely related genome? Is this just chance?<br>Response: We agreed with both the editor and reviewer, the selection analysis is not very useful here and also need more work for it. Therefore, we removed the selection analysis section, and more focused on the sequencing, assembly and annotation parts.

Powered by Editorial Manager® and ProduXion Manager® from Aries Systems Corporation

Reviewer #2

General comments
In this article the authors present the first draft genome of the forest musk deer Moschus berezovskii. They provide a brief description of the sequencing, assembly and annotation process. This is a typical draft genome paper with little biological insight, but considering the status of the species and the little amount of available data on it, I believe that this contribution will be of great value to the community - if data and results are made available. I have two main concerns with this paper:

(1) I would need more details about the methods. It should be made possible to redo all the experiments and all the analyses that are mentioned in the manuscript. In particular, parameters and versions of each software tool have to be precised. Experimental protocols need more details too. One way to do this is to provide some more information in the main text and to complete with all the details in the Supplementary Methods. So far this document only contains the description of the phylogenetic analysis. The same should be done for the others.
Response: We added the parameters, software versions, and details of the protocols throughout the whole manuscript.

(2) The genomic sequence and its annotation should be made available. I could not find them. Sequencing reads have been deposited in the SRA but I would appreciate if the authors provide the results from the assembly (fasta format) and from the gene annotation (gtf or gff3 format for instance). This is actually the main value of the study.
Response: We have uploaded the information to GigaScience's database, and we have communicated with the editor to make sure all the data is available.

More specific comments to the authors
1. Sampling/sequencing
L112-114: please provide more details about library construction (DNA extraction, protocol, kit, etc). I expect some to be PE and others mate-pairs. Could you precise? Also, I am not sure the read length is specified.
Response: We extracted genomic DNA from tissue samples using the Qiagen DNeasy Blood and Tissue Kit (Qiagen, Valencia, USA) following the manufacturer's protocol. We added the information into the "Sample information and sequencing" section (Lines 112 - 119). We also added the read length information into the Table 1. The details were given in the new supplementary note.

L115-116: what kind of filtering/cleaning has been made and how? Please provide details about quality and adapter trimming, including the name of the software.
Response: We provided the details within the supplementary note (sequencing and filtering raw data section).

2. Assembly
L120-121: how did you estimate the genome size? Method, software, version? Why 17-mers?
Response: We used GCE (v1.0) to perform kmer analysis, and the 17-mer was suggested by the GCE results. The details could be found within the supplementary note.

L122: "the assembly was first analyzed by SOAPdenovo2" => don't you mean "generated"? The assembly needs to be produced before being analyzed.
Response: Yes, it should be "generated" and we corrected.

L125: what was the proportion of gaps before and after gapcloser?
Response: We explained it within the supplementary note.

L124: how was SSPACE used? How many scaffolds before and after? Also, please precise the version.
Response: We explained it within the supplementary note.

L129: Table 2 is way too short -only three numbers!- to give a decent description of the

Powered by Editorial Manager® and ProduXion Manager® from Aries Systems Corporation

assembly. Please give the number of contigs, of scaffolds, the size of the longest ones, the GC%, etc…
See the same tables in similar publications from the same journal, for instance:
https://academic.oup.com/gigascience/article/4077042/The-draft-genome-sequence-of-a-desert-tree-Populus
https://academic.oup.com/gigascience/article/6/8/1/4004833/Draft-genome-of-the-Antarctic-dragonfish
https://academic.oup.com/gigascience/article/6/6/1/3748232/Draft-genome-of-the-lined-seahorse-Hippocampus
More generally, please also consider these previous publications to get an idea about the amount of details that are expected from this kind of report.
Response: We added more information within Table 2.

L132: CEGMA + BUSCO: Cegma is no longer maintained and should not be used anymore.
Response: Yes, we deleted the Cegma result and former Table S1 (the results for Cegma).

L136: please cite the study that generated the RNA-seq data you used.
Response: We added the SRA ID for the RNA-seq data (SRR2098995 and SRR2098996; Line 138) since there was no publication.

L138: the proportion of mapped reads is high and this is a good point, but it would be more informative to also show the proportion of concordant pairs, assuming that the RNA-seq data is PE. This assembly section is rather descriptive and technical but it is difficult to estimate up to which point all these steps were useful. A nice way to show the value of this work could be to compare the number of mapped reads and concordant read pairs from the RNA-seq and DNA-seq libraries (of the different sizes) before and after the gap filling and scaffolding part.
Response: In total of 92.73% PE reads were concordantly aligned to forest musk deer genome. We added the information into the manuscript (Lines 137 - 138).

3. Annotation
L147: how was Augustus trained? Was a training set of known genes provided to estimate the parameters?
Response: Yes, we used some protein sequences of Bos taurus to train the model for Augustus (version 3.0.3). We provided the information within supplementary note.

L148: "analyzed" => aligned, I guess
Response: Yes, we corrected the word.

L150-151: what is "software solar"? Please explain how GeneWise was used and provide details about potential filtering and other steps after the blast.
Response: We explained within the main text (Lines 178 – 180)

L153: Trinity has been used in both genome guided and de novo mode: why? What were the differences? Please provide the parameters. How did you merge the results?
Response: This method was followed by Trinity manual in the section of ' Build a Comprehensive Transcriptome Database Using Genome-guided and De novo RNA-Seq Assembly ', website is https://pasapipeline.github.io/.

L154: Please cite EVM properly. How did you use it? What did you choose for the confidence weights? Could you provide the input files from the distinct sources before merging?
Response: We cited the reference "Automated eukaryotic gene structure annotation using EVidenceModeler and the Program to Assemble Spliced Alignments, Genome Biology 2008". Other details could be found in the supplementary note.

L156: manual annotation can be a huge amount of work. Were there many modifications made? If so, it would be interesting and probably impressive to illustrate the contribution of this work by comparing annotation stats (see below) or other metrics before and after this polishing step, and/or to describe the most common corrections (gene splitting/merging? Splice site fixing? etc). That is only a suggestion.

	Response: Yes, the manual annotation will require lots of work. Therefore, we only manually checked the scaffolds that size longer than 1Mb. In addition, we focused on gene splitting or merging.<br><br>Before the GO functional annotation, something that is missing is the description of the annotation with more statistics than just the number of genes, especially given the draft status of the genome assembly. In particular a simple table could present some stats about the gene length distribution (min, max, median, average), distribution of predicted ORFs/CDS (idem), number of exons per gene (idem: min, max, mean and median).<br>Response: We added a new supplementary table (current Table S2) to provide the length of the gene, CDS and exon.<br><br>Also, it would be useful to illustrate the quality of the provided annotation by comparing it with other available datasets. For instance, what is the percentage of RNA-seq mapped reads that fall within annotated exons? That are consistent with the predicted gene models? How do the annotated transcripts compare with those from the already published transcriptome study (ies)?<br>Response: Furthermore, we downloaded musk gland RNA-seq data (SRA accession: SRR2098995 and SRR2098996) of forest musk deer from NCBI to evaluate the assembly. We found that 99.3% of the total PE reads could be aligned (92.73% aligned concordantly) to the assembled forest musk deer genome by Bowtie2 (version 2.2.5). We added this within the main text (Lines 133 - 167).<br><br>4. Repeats<br>L190: "We also analyzed the degree of divergence for each type of TE" => How? Again: method, software, version, and parameters. Same for MSDB.<br>Response: We updated the method, software, version and parameters (Lines 219 – 239).<br><br>The number of TEs is compared across species: could the authors make sure that the same method was used to detect them in each species? Otherwise it could just be due to the method. Please keep in mind that all these annotated "genes", "TEs" etc, are only computational predictions.<br>Response: Yes, they were detected with different methods although the methods were very similar. We still should be careful when we compared the numbers.<br><br>5. Gene families<br>See general concern (1). Please also indicate the version of the ENSEMBL and NCBI annotation.<br>Response: Yes, we added the information into the table (current Table S5).<br><br>6. Olfactory Receptor genes<br>L218: typos in "pseudogenes" and “truncated".<br>Response: Thanks, we corrected the words.<br><br>L219: The number of OR genes is compared across species and a "degeneration of OR genes in primates" is mentioned. Couldn't the difference be due to the fact that these OR genes were not annotated the same way between species (Sup Tables)?<br>Response: Yes, therefore, we removed the comparison, and reviewer 1 also asked us to simplify this part (Lines 260 - 264).<br><br>Table S2: 19 / 303 = 20.51% ? ("missing busco" part)<br>Response: It was a mistake, we corrected the numbers and now, it is now within Table S1.<br><br>Table S5: It is ensembl, not ensemble. Please precise the version.<br>Response: We corrected the word, and also provided the version and other related information.
**Additional Information:**	
**Question**	**Response**
Are you submitting this manuscript to a	No

Powered by Editorial Manager® and ProduXion Manager® from Aries Systems Corporation

special series or article collection?	
**Experimental design and statistics**<br><br>Full details of the experimental design and statistical methods used should be given in the Methods section, as detailed in our Minimum Standards Reporting Checklist. Information essential to interpreting the data presented should be made available in the figure legends.<br><br>Have you included all the information requested in your manuscript?	Yes
**Resources**<br><br>A description of all resources used, including antibodies, cell lines, animals and software tools, with enough information to allow them to be uniquely identified, should be included in the Methods section. Authors are strongly encouraged to cite Research Resource Identifiers (RRIDs) for antibodies, model organisms and tools, where possible.<br><br>Have you included the information requested as detailed in our Minimum Standards Reporting Checklist?	Yes
**Availability of data and materials**<br><br>All datasets and code on which the conclusions of the paper rely must be either included in your submission or deposited in publicly available repositories (where available and ethically appropriate), referencing such data using a unique identifier in the references and in the "Availability of Data and Materials" section of your manuscript.<br><br>Have you have met the above requirement as detailed in our Minimum Standards Reporting Checklist?	Yes

Manuscript Click here to download Manuscript MD_GS_R1_1221.docx

The draft genome sequence of forest musk deer (*Moschus berezovskii*)

Zhenxin Fan[1,†], Wujiao Li[1,†], Jiazheng Jin[1], Kai Cui[1], Chaochao Yan[1], Changjun Peng[1], Zuoyi Jian[1], Megan Price[1], Xiuyue Zhang[1], Yongmei Shen[3], Jing Li[1], Wenhua Qi[2,*], Bisong Yue[1,*]

[1] Key Laboratory of Bioresources and Ecoenvironment (Ministry of Education), College of Life Sciences, Sichuan University, Chengdu 610064, People's Republic of China

[2] College of Life Science and Engineering, Chongqing Three Gorges University, Chongqing 404100, People's Republic of China

[3] Sichuan Engineering Research Center for Medicinal Animals, Xichang 615000, People's Republic of China

† Contributed equally to this work

*** Corresponding author:** Bisong Yue (bsyue@scu.edu.cn), and Wenhua Qi (wenhuaqi357@163.com)

Emails: zxfan@scu.edu.cn (Zhenxin Fan); hnnd059@gmail.com (Wujiao Li); wenhuaqi357@163.com (Wenhua Qi); jinjiazhengxiao@163.com (Jiazheng Jin); 2015222040118@stu.scu.edu.cn (Kai Cui); yccvican@gmail.com (Chaochao Yan); jj-5380682@163.com (Changjun Peng); jzuoyi@126.com (Zuoyi Jian); meganprice@scu.edu.cn (Megan Price); zhangxy317@126.com (Xiuyue Zhang); Yongmei Shen (810316122@qq.com); Jing Li (ljtjf@126.com); bsyue@scu.edu.cn (Bisong Yue)

Abstract

Background: The forest musk deer, *Moschus berezovskii*, is one of seven musk deer (*Moschus* spp.) and is distributed in Southwest China. Akin to other musk deer, the forest musk deer has been traditionally, and is currently, hunted for its musk (i.e. global perfume industry). Considerable hunting pressure and habitat loss has caused significant population declines and therefore the Chinese government commenced captive breeding programs for musk harvesting in the 1950s. However, the prevalence of fatal diseases is considerably restricting population increases. Disease severity and extent is exacerbated by inbreeding and genetic diversity declines in captive musk deer populations. It is essential for the physical and genetic health of captive and wild forest musk deer populations to improve knowledge of its immune system and genome. We have thus sequenced the whole genome of the forest musk deer, completed the genomic assembly and annotation, and performed preliminary bioinformatic analyses.

Findings: A total of 407 Gb raw reads from whole-genome sequencing was generated by the Illumina Hiseq4000 platform. The final assembly genome is around 2.72 Gb, with a contig N50 length of 22.6 kb and a scaffold N50 length 2.85 Mb. We identified 24,352 genes, and found 42.05% of the genome is composed of repetitive elements. We also detected 1,236 olfactory receptor genes. The genome-wide phylogenetic tree indicated that the forest musk deer was within the order Artiodactyla, and it appeared as the sister clade of four members of family Bovidae. In total, 576 genes were under positive selection in the forest musk deer lineage.

Conclusions: We provide the first genome sequence and gene annotation for the forest musk deer. The availability of these resources will be very useful for the conservation and captive breeding for this Endangered and economically important species, and for reconstructing the evolutionary history of the order Artiodactyla.

Keywords: Forest musk deer; whole genome sequencing; genome assembly; annotation; phylogeny

Data Description

1) Background

The seven musk deer species of genus *Moschus* are endemic to Asia, are currently listed under Appendix II in CITES and are listed under Category I of the State Key Protected Wildlife List of China [1-3]. All musk deer species are considered as globally threatened, with six being listed as Endangered and one as Vulnerable by the IUCN [4]. *Moschus* is the only extant genus of Moschidae and musk deer are considered as primitive deer. The genus of musk deer is characterized by the musk secreted by the scent glands of adult males [5]. The forest musk deer (*Moschus berezovskii*) is one of the five recognized musk deer species of China and have historically been distributed in Southwest China [6,7]. The forest musk deer has been listed as globally endangered, as Critically Endangered on the 2015 China Red List, and is also on the State Key Protected Wildlife List of China [4].

Musk deer have been hunted for thousands of years, as the musk has been widely used in traditional Chinese medicines. In the last two centuries, hunting of all musk deer species significantly increased for the global trade of the commercially valuable musk secretion as an essential basis for perfume manufacture [5]. Since the 1950s, populations of forest musk deer have declined dramatically from poaching of deer for the musk pods (i.e. entire gland) and significant habitat destruction [3,6,8]. As a consequence, the Chinese government has encouraged musk-using enterprises to participate in artificial breeding programs since the early 1950s [9]. The musk can be collected from

male musk deer in these captive populations without harvesting individuals, further enhancing the commercial and conservation value of captive populations.

The captive population of the forest musk deer is the largest among all the musk deer species [2,10]. The Miyaluo farming population in Sichuan Province (China) was one of the earliest established captive breeding populations. This population had grown rapidly to approximately 400 in 2010 [10]. However, the prevalence of fatal diseases is considerably restricting population increases [11]. Common diseases of forest musk deer in the Miyaluo population are dyspepsia, pneumonia, metritis, urinary stones and abscesses, with abscesses being one of the most prevalent causes of death [7]. Disease severity and extent is exacerbated by inbreeding and genetic diversity declines in this and other captive musk deer populations.

It is essential for the physical and genetic health of captive and wild forest musk deer populations to improve knowledge of its immune system and genome. We have thus sequenced the whole genome of the forest musk deer, subsequently completed the genomic assembly and annotation, and performed preliminary bioinformatic analyses, such as phylogenetic tree, selection and gene enrichments.

2) Sample information and sequencing

The thigh muscle sample was collected from a Miyaluo male forest musk deer that naturally died (Sichuan Province, China) in 2015. We extracted genomic DNA from the muscle sample using the Qiagen DNeasy Blood and Tissue Kit (Qiagen, Valencia, USA) following the manufacturer’s protocol. We constructed

six different insert size libraries: 230bp, 500bp, 2kb, 5kb, 10kb, and 15kb. These libraries were sequenced by Illumina Hiseq 4000 platform at Novogene (Beijing, China). A total of 407Gb of raw data were generated, after filtering out low quality, duplicate and adaptor polluted reads. Approximately 360Gb of high-quality reads were retained for genome assembly (Table 1).

3) Genome assembly and evaluation

We use GCE (version 1.0) to performed k-mer (17-mer) analysis by short insert size library reads before assembly, and the forest musk deer genome size was estimated to be 2.95Gb (Figure S1). The assembly was first generated by SOAPdenovo2 [12] with the parameters set as "all -d 2 –M 2 –k 35". Intra-scaffold gaps were filled using Gapcloser (version 1.12) with reads from 230bp and 500bp libraries, and then SSPACE (version 3.0) [13] was used to build super-scaffolds. After scaffolding by SSPACE, we used Gapcloser again to fill gaps. Finally we obtained the forest musk deer genome with a size of 2.72Gb (all the sequences with length shorter than 300bp were removed) with 125.7Mb gap sequences unsolved. The N50s of contigs and scaffolds of forest musk deer genome were 22.6kb and 2.85Mb, respectively (Table 2).

We used BUSCO (version 3.0) to evaluate the genome complement. BUSCO results showed that 84.5% of the eukaryotic single-copy genes were captured (Table S1). Furthermore, we downloaded musk gland RNA-seq data (SRA accession: SRR2098995 and SRR2098996) of forest musk deer from NCBI to evaluate the assembly. We found that 99.3% of the total PE reads could be aligned (92.73% aligned concordantly) to the assembled forest musk deer

genome by Bowtie2 (version 2.2.5) [14]. These results showed our forest musk deer genome was of high quality, and was suitable as a reference genome for the family Moschidae.

4) Annotation

We combined the *de novo*, homology-based and transcriptome-based prediction to identify protein-coding genes in the forest musk deer genome. The software Augustus (version 3.2.1) [15] was used for *de novo* prediction based on the parameter trained for forest musk deer. For homology prediction, protein sequences from four mammals (human, pig, sheep and cattle) were analyzed with TBLASTN (BLAST version 2.2.26) against forest musk deer genome. Potential gene regions were joined by SOLAR (version 0.9.6) [16], and the coding sequence with 500bp flanking sequence were cut down and re-aligned by GeneWise (version 2.4.1 with parameters "- sum - genesf -gff") [17]. For transcriptome-based prediction, musk gland RNA-seq data were assembled by Trinity with genome guide and *de novo* mode, respectively. The gene structures were obtained by PASA pipeline (version 2.0.2) [18]. We used EVM (version 1.1.1) to integrate the above evidence and obtained a consensus gene set [19]. Apollo (version 1.11.6) was performed to manually inspect gene structure in scaffolds of sizes above 1Mb to gain a more accurate gene structure. We consequently found a total of 24,352 genes predicted to be present in the forest musk deer genome. We also provided the length of genes in Table S2.

Functional annotation of forest musk deer genes was undertaken based on the best match derived from the alignments to proteins annotated in Swiss-

Prot and TrEMBL databases [20]. Functional annotation used BlastP tools with the same E-value cut-off of 1E-5. We also annotated proteins against the NCBI non-redundant (nr) protein database. The outputs of blast searching against the NCBI nr protein database were imported into BLAST2GO (B2G4PIPE v2.5) for Gene Ontology (GO) [21] term mapping. Term mapping used annotated motifs and domains using InterProScan (interproscan-5.18-57.0) [22] by searching against publicly available databases. To find the best match for each gene, KEGG pathway maps were used by searching KEGG databases [23] through the KEGG Automatic Annotation Server (KAAS) using the bi-directional best hit (BBH) method. In total, 23,023 out of 24,352 (94.5%) protein-coding genes were searched within the publicly available functional databases of TrEMBL, Swiss-Prot, Interpro, GO and KEGG. Of which, 22,696 (93.20% TrEMBL), 18,771 (77.08% Swiss-Prot), 22,221 (91.12% Interpro), 15,736 (64.62% GO) and 10,846 (44.54% KEGG) genes showed significant similarity matches (Figure 1; Table 3). The functional comparisons with two closely related species (cattle and sheep) for GO classification were submitted to the WEGO [24] (Figure S2).

5) Repetitive sequences and transposable elements

Transposable elements (TEs) and other repeats make up a substantial fraction of mammalian genomes and contribute to gene and/or genome evolution [25]. The TE content, type, copy number, subfamily, and divergence rate were investigated in the forest musk deer genome based on two strategies: the library based strategy of RepeatMasker [26] and the *de novo* based strategy of RepeatScout [27]. The forest musk deer genome has large numbers of TEs, comprising 42.05%

of the genome (Table S3), which is similar to those of cattle (46.5%) [25] and goats (42.2%) [28]. The 23 different types of TEs have been grouped for the four different types of TEs, such as DNA transposons, LTR, LINE, and SINE retrotransposons (Figure S3). The LINEs were the most common repeats in forest musk deer genome; followed by SINEs > LTR > DNA. We also analyzed the degree of divergence for each type of TE in the forest musk deer genome. We found there was a recent burst activity involving LINE transposons and a second, older burst activity of LTR and DNA transposons (Figure S3).

A total of 542,135 microsatellites (simple sequence repeats, SSRs) were identified by software MSDB [29] in the forest musk deer genome assembly (Table S4), which accounted for 0.45% of its whole genome length. Mononucleotide SSRs were the most abundant category, accounting for 41.75% of all of the SSRs; followed by followed by: di- > tri- > tetra- > penta- > hexa nucleotide SSRs (Table S4).

6) Gene families

To estimate species-specific and shared genes in the forest musk deer in comparison to ten mammal species, we used orthoMCL [30] to define the orthologous genes. We downloaded the genomes and gene annotations of the ten additional species (human, horse, dog, cattle, mouse, yak, sheep, Tibetan antelope, alpaca, and pig) from Ensembl [31] or NCBI (Table S5). In total, we identified 18,855 homologous gene families shared by forest musk deer and the ten additional species, 221 gene families that were specific to forest musk deer, and 2,003 gene families found in the ten additional species but not in the forest

musk deer (Figure S4). In addition, we found 5,372 one-to-one orthologous genes within forest musk deer and the ten species, which was used in phylogenetic analyses. In addition, we detected olfactory receptor (OR) genes in the forest musk deer genome by orfam (https://github.com/jianzuoyi/orfam) since they formed the largest gene family in mammalian genomes [32]. In total, we identified 1,236 OR genes, which included 866 intact, 266 pseduogenes, and 104 truncated genes.

7) Phylogenetic analysis

We constructed the phylogenetic trees based on Bayesian inference (BI) [33] and maximum likelihood (ML) [34,35] analyses with the discovered 5,372 one-to-one orthologous genes (Supplementary notes). All the different methods generated the same topology and obtained the well-supported phylogenetic tree (Figure 2). The forest musk deer was within the suborder Ruminantia, order Artiodactyla, and it appeared as the sister clade of four members of family Bovidae (sheep, yak, cattle, and Tibetan antelope). Since we do not have high quality genome sequences for species within family Cervidae, the relationship between Moschidae, Cervidae, and Bovidae at the genomic level is tentative and needs further investigation.

Conclusions

Here, we report the first draft genome assembly of the forest musk deer genome, a species that is of particular importance to China's ecology, biodiversity conservation, economy, and medicine. The availability of the genome and these

results will be very useful for the conservation and captive breeding of this Endangered and economically important species, and for reconstructing the evolutionary history of the order Artiodactyla.

Funding

This work was supported by National Key Program of Research and Development, Ministry of Science and Technology (2016YFC0503200), and National Natural Science Foundation of China (31702032).

Availability of supporting data

The DNA sequencing data have been deposited into the NCBI Sequence Read Archive (SRA) under the ID PRJNA317652.

Conflicts of interest

The authors declare that they have no competing interests.

Author's contributions

Z.F., X.Z., J.L., and B.Y. designed and supervised the project. Z.F., W.L., C.Y., J.J., C.P., J.Y., Y.S., and K.C. performed the bioinformatics analyses. M.P. revised the manuscript. Z.F. and B.Y. wrote the manuscript.

Figure Legend

Figure 1 Functional annotation statistics. Venn diagram illustrating distribution of high-score matches of the functional annotation in forest musk deer genome from five public databases.

Figure 2 Genome wide phylogenetic trees. We constructed the phylogenetic trees based on Bayesian inference and maximum likelihood analyses with 5,372 one-to-one orthologous genes between the forest musk deer and ten other species.

Figure S1 K-mer (k=17) distributions in forest musk deer genome.

Figure S2 GO comparative analysis and functional classification between forest musk deer, sheep and cattle.

Figure S3 Distribution of divergence of each type of TEs in the forest musk deer genome. The divergence rate was calculated between the identified TE elements in the genome and the consensus sequence in the TE library used. SINEs: Short interspersed elements, LINEs: Long interspersed elements, LTR: Long terminal repeat retrotransposon.

Figure S4 Protein orthology comparison between different genomes. There were forest musk deer (*Moschus bweezovskii*), cattle (*Bos taurus*), yak (*Bos grunniens*), sheep (*Ovis aries*), Tibetan antelope (*Pantholops hodgsonii*), alpaca (*Vicugna pacos*), and pig (*Sus scrofa*), which representing Artiodactyla; human (*Homo sapiens*, Primates), horse (*Equus caballus*, Perissodactyla), and dog (*Canis lupus familiaris*, Carnivora), mouse (*Mus musculus*, Rodentia). For each animal, proteins are represented by bars and classified according to orthoMCL analysis: Single_copy (Oliver) include the common orthologs with the same number of

copies in different species; Multi_copy (Red) include the common orthologs with different copy numbers in the different species; Unique (Magenta) include the orthologs just in one species; Unclustered gene (Yelollow) include the genes that cannot be clustered into known gene families; Other (Blue) include the genes that can be clustered into known gene families, but it not belongs to Single, Multi or Unique.

References

1. Cobert GB, Hill JE. The mammals of the Indomalaysia region: a systematic review. London (UK): Natural History Museum Publications Oxford University Press. 1992.

2. Wu J, Wang W. The Musk Deer of China. The China Forestry Publishing House, Beijing. 2006.

3. Sheng H, Liu Z. The Musk Deer in China. The Shanghai Scientific & Technical Publishers, Shanghai. 2007.

4. IUCN. The IUCN Red List of Threatened Species. 2017 (http://www.iucnredlist.org/)

5. Green MJB. The distribution, status and conservation of the Himalayan musk deer (Moschus chrysogaster). Biol. Conserv. 1986;35, 347–75.

6. Sheng H. Genus Moschus in China. In: Wang, S. (Ed.), China Red Data Book of Endangered Animals. Science Press, Beijing. 1998.

7. Zhao K, Liu Y, Zhang X, et al. Detection and characterization of antibiotic-resistance genes in Arcanobacterium pyogenes strains from abscesses of forest musk deer. J Med Microbiol. 2011;60:1820-6.

8. Yang Q, Meng X, Xia L, Feng Z. Conservation status and causes of decline of musk deer (Moschus spp.) in China. Biol. Conserv. 2003;109, 333–342.

9. Peng H, Liu S, Zou F, Zeng B, Yue B. Genetic diversity of captive forest musk deer (Moschus berezovskii) inferred from the mitochondrial DNA control region. Anim Genet. 2009;40(1):65-72.

10. Huang J, Li Y, Li P, et al. Genetic quality of the Miyaluo captive forest musk deer (Moschus berezovskii) population as assessed by microsatellite loci. Biochemical Systematics & Ecology, 2013;47(8):25-30.

11. Lu X, Qiao J, Wu X, Su L. A review of mainly affected on musk-deer diseases: purulent, respiratory system and parasitic diseases. J Economic Anim 2009, 13, 104–107.

12. Luo R, Liu B, Xie Y, et al. SOAPdenovo2: an empirically improved memory-efficient short-read de novo assembler. Gigascience 2012;1(1):18.

13. Boetzer M, Henkel CV, Jansen HJ, Butler D, Pirovano W. Scaffolding pre-assembled contigs using SSPACE. Bioinformatics. 2011;27(4):578-9.

14. Langmead B, Salzberg SL. Fast gapped-read alignment with Bowtie 2. Nat Methods. 2012;9(4):357-9.

15. Stanke M, Diekhans M, Baertsch R, Haussler D. Using native and syntenically mapped cDNA alignments to improve de novo gene finding. Bioinformatics. 2008;24(5):637-44.

16. Yu XJ, Zheng HK, Wang J, Wang W, Su B. Detecting lineage-specific adaptive evolution of brain-expressed genes in human using rhesus macaque as outgroup. Genomics. 2006;88(6):745-751.

17. Birney E, Clamp M, Durbin R. GeneWise and Genomewise. Genome Res. 2004;14(5):988-95.

18. Haas BJ, Delcher AL, Mount SM, et al. Improving the Arabidopsis genome annotation using maximal transcript alignment assemblies. Nucleic Acids Res. 2003;31(19):5654-66.

19. Haas BJ, Salzberg SL, Zhu W, Pertea M, Allen JE, Orvis J, White O, Buell CR, Wortman JR. Automated eukaryotic gene structure annotation using EVidenceModeler and the Program to Assemble Spliced Alignments. Genome Biol. 2008;9(1):R7.

20. Boeckmann B, Bairoch A, Apweiler R, et al. The SWISS-PROT protein knowledgebase and its supplement TrEMBL in 2003. Nucleic Acids Res. 2003;31(1):365-70.

21. Gene Ontology Consortium. Gene Ontology annotations and resources. Nucleic Acids Res. 2013;41(Database issue):D530-5.

22. Hunter S, Apweiler R, Attwood TK, et al. InterPro: the integrative protein signature database. Nucleic Acids Res. 2009;37(Database issue):D211-5.

23. Kanehisa M, Goto S. KEGG: kyoto encyclopedia of genes and genomes. Nucleic Acids Res. 2000;28(1):27-30.

24. Ye J, Fang L, Zheng H, et al. WEGO: a web tool for plotting GO annotations. Nucleic Acids Res. 2006;34(Web Server issue):W293-7.

25. Adelson DL, Raison JM, Edgar RC. Characterization and distribution of retrotransposons and simple sequence repeats in the bovine genome. Proc Natl Acad Sci U S A. 2009;106(31):12855-60.

26. Smit AFA, Hubley R, Green P. 2016. RepeatMasker website and server[CP/OL]. (2016-9-12)[2016-10-15]. http://www.repeatmasker.org/.

27. Price AL, Jones NC, Pevzner PA. De novo identification of repeat families in large genomes. Bioinformatics. 2005;21 Suppl 1:i351-8.

28. Dong Y, Xie M, Jiang Y, et al. Sequencing and automated whole-genome optical mapping of the genome of a domestic goat (Capra hircus). Nat Biotechnol. 2013;31(2):135-41.

29. Du L, Li Y, Zhang X, Yue B. MSDB: a user-friendly program for reporting distribution and building databases of microsatellites from genome sequences. J Hered. 2013;104(1):154-7.

30. Li L, Stoeckert CJ Jr, Roos DS. OrthoMCL: identification of ortholog groups for eukaryotic genomes. Genome Res. 2003;13(9):2178-89.

31. Yates A, Akanni W, Amode MR et al. Ensembl 2016. Nucleic Acids Res. 2016;44(D1):D710–16.

32. Firestein S. How the olfactory system makes sense of scents. Nature. 2001;413(6852):211-8.

33. Ronquist F, Huelsenbeck JP. MrBayes 3: Bayesian phylogenetic inference under mixed models. Bioinformatics. 2003;19(12):1572-4.

34. Guindon S, Dufayard JF, Lefort V, et al. New algorithms and methods to estimate maximum-likelihood phylogenies: assessing the performance of PhyML 3.0. Syst Biol. 2010;59(3):307-21.

35. Stamatakis A, Hoover P, Rougemont J. A rapid bootstrap algorithm for the RAxML Web servers. Syst Biol. 2008;57(5):758-71.

Click here to download Table Table 1_sequencing.docx

Table 1 Genome sequencing information.

Insert size (bp)	Read length (bp)	Raw data		Clean data	
		Total bases (Gb)	Sequencing depth (x)	Total bases (Gb)	Sequencing depth (x)
230	125	135.76	46.02	125.96	42.70
500	125	102.51	34.75	88.52	30.01
2,000	125	59.0	20.00	50.16	17.00
5,000	125	51.57	17.48	46.39	15.73
10,000	125	28.16	9.55	24.67	8.36
15,000	125	30.34	10.28	28.14	9.54
Total		407.34	138.08	363.84	123.34

Note: Genome size is 2.95Gb.

 Click here to download Table Table 2_new.docx

Table 2 Statistics of the final assembly of forest musk deer genome.

Genome assembly	Numbers
Contig N50 (kb)	22.6
Scaffold N50 (Mb)	2.85
Longest Scaffold(Mb)	18.69
Scaffold Number	79,206
GC content	40%
Total length (Gb)	2.72

Click here to download Table Table 3 _FunctionalAnnotation.docx

Table 3 Functional annotation statistics of forest musk deer genome by various methods

	Database	Number	Percent (%)
Total		24,352	100.00
	Swissprot	18,771	77.08
	TrEMBL	22,696	93.20
Annotated	KEGG	10,846	44.54
	Interpro	22,221	91.12
	GO (blast2go)	15,736	64.62
	GO (Interproscan)	14,815	60.84
Un-annotated		1,329	5.77

Figure 1

Click here to download Figure Figure 1_annotation.tif

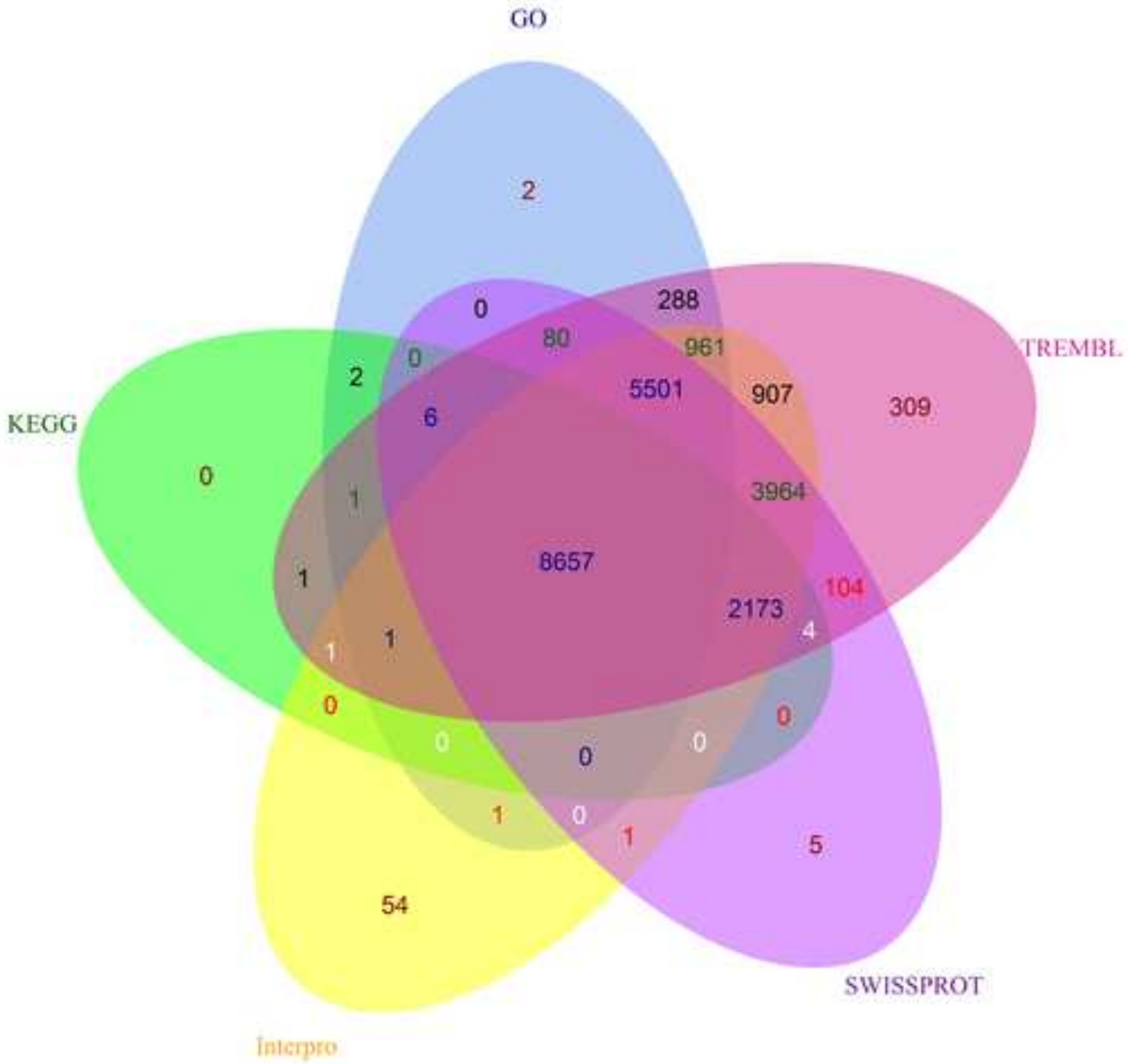

Figure 2

Click here to download Figure Figure 2_tree.tif

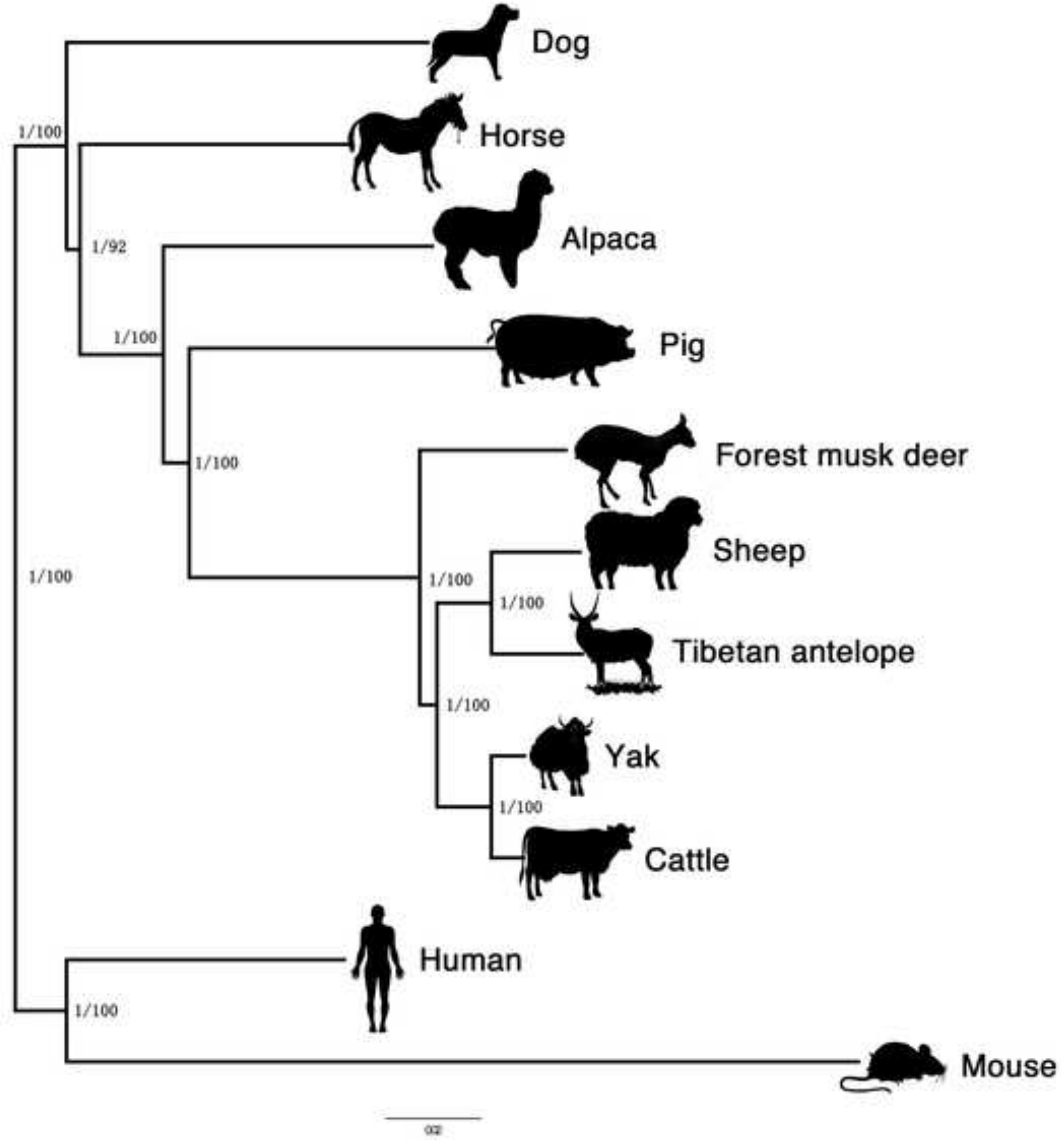

Supplementary Notes

Click here to access/download
Supplementary Material
MD_R1_suppNote.docx

Supplementary Table S1

Click here to access/download
Supplementary Material
Table S1_BUSCO.docx

Supplementary Table S2

Click here to access/download
Supplementary Material
Table S2_new.docx

Supplementary Table S3

Click here to access/download
Supplementary Material
Table S3_repeat.docx

Supplementary Table S4

Click here to access/download
Supplementary Material
Table S4_SSR.docx

Supplementary Table S5

Click here to access/download
Supplementary Material
Table S5_Orthologous.xlsx

Supplementary Figure S1

Click here to access/download
Supplementary Material
Figure S1_kmer.tif

Supplementary Figure S2

Click here to access/download

Supplementary Material

Figure S2_wego.pdf

Supplementary Figure S3

Click here to access/download
Supplementary Material
Figure S3_TE_type.tif

Supplementary Figure S4

Click here to access/download
Supplementary Material
Figure S4_OrthologousGene.tif

Cover letter
Click here to download Personal Cover GigaSci_Cover_New.docx

Dear Editor,

Please find attached our manuscript entitled “The draft genome sequence of forest musk deer (Moschus berezovskii)” that we would like considered for publication in *GigaScience*. We have carefully revised the manuscript in accordance with reviewers’ comments as detailed in the attached material. Although there were many comments requiring changes to the manuscript, we feel the comments and needed actions were straightforward and have thus been able to accommodate nearly every concern by changes to the text. We hope you will now find the manuscript acceptable for publication. We declare that the manuscript is not published, in press, or simultaneously submitted elsewhere. All authors have read and approved the manuscript, and declared that there were no competing interests.

Please address all correspondence concerning this manuscript to Dr Zhenxin Fan.

Sincerely,

Dr. Zhenxin Fan
College of Life Sciences, Sichuan University, Cheng Du, Sichuan 610065, China
E-mail: zxfan@scu.edu.cn
