# Supplementary material for: The draft genome sequence of forest musk deer (Moschus berezovskii)
Source: Gigascience. 2018 Apr 9;7(4):giy038. doi: 10.1093/gigascience/giy038 (PMC5906906; doi:10.1093/gigascience/giy038)
Supplement: GIGA-D-17-00185_Revision_2.pdf [file giy038_giga-d-17-00185_revision_2.pdf]

GigaScience

The draft genome sequence of forest musk deer (Moschus berezovskii)

--Manuscript Draft--

**Manuscript Number:**	GIGA-D-17-00185R2
**Full Title:**	The draft genome sequence of forest musk deer (Moschus berezovskii)
**Article Type:**	Data Note
**Funding Information:**	National Key Program of Research and Development, Ministry of Science and Technology (2016YFC0503200) — Dr. Bisong Yue<br>National Natural Science Foundation of China (31702032) — Dr. Wenhua Qi
**Abstract:**	Background: The forest musk deer, Moschus berezovskii, is one of seven musk deer (Moschus spp.) and is distributed in Southwest China. Akin to other musk deer, the forest musk deer has been traditionally, and is currently, hunted for its musk (i.e. global perfume industry). Considerable hunting pressure and habitat loss has caused significant population declines and therefore the Chinese government commenced captive breeding programs for musk harvesting in the 1950s. However, the prevalence of fatal diseases is considerably restricting population increases. Disease severity and extent is exacerbated by inbreeding and genetic diversity declines in captive musk deer populations. It is essential for the physical and genetic health of captive and wild forest musk deer populations to improve knowledge of its immune system and genome. We have thus sequenced the whole genome of the forest musk deer, completed the genomic assembly and annotation, and performed preliminary bioinformatic analyses.<br>Findings: A total of 407 Gb raw reads from whole-genome sequencing was generated by the Illumina HiSeq 4000 platform. The final assembly genome is around 2.72 Gb, with a contig N50 length of 22.6 kb and a scaffold N50 length 2.85 Mb. We identified 24,352 genes, and found 42.05% of the genome is composed of repetitive elements. We also detected 1,236 olfactory receptor genes. The genome-wide phylogenetic tree indicated that the forest musk deer was within the order Artiodactyla, and it appeared as the sister clade of four members of family Bovidae. In total, 576 genes were under positive selection in the forest musk deer lineage.<br>Conclusions: We provide the first genome sequence and gene annotation for the forest musk deer. The availability of these resources will be very useful for the conservation and captive breeding for this Endangered and economically important species, and for reconstructing the evolutionary history of the order Artiodactyla.
**Corresponding Author:**	Zhenxin Fan, Ph.D.<br>Sichuan University<br>Chengdu, Sichuan CHINA
**Corresponding Author Secondary Information:**	
**Corresponding Author's Institution:**	Sichuan University
**Corresponding Author's Secondary Institution:**	
**First Author:**	Zhenxin Fan, Ph.D.
**First Author Secondary Information:**	
**Order of Authors:**	Zhenxin Fan, Ph.D.<br>Bisong Yue<br>Wujiao Li<br>Chaochao Yan<br>Jing Li

Powered by Editorial Manager® and ProduXion Manager® from Aries Systems Corporation

	Yongmei Shen
	Wenhua Qi
**Order of Authors Secondary Information:**	
**Response to Reviewers:**	Reviewer #1<br><br>The paper by Fan et al. reports the genome assembly of the forest musk deer (Moschus berezovskii). The species is globally threatened and listed under CITES Appendix II, yet a relatively robust commercial farming (for musk) industry exists in China. Genomic resources will likely be informative for management, notably breeding programs and limiting disease transmission (Sun et al. 2018 Sci. Rep).<br><br>1. This is my second time reviewing the article and as noted previously, the genome assembly reflects the industry standard. The paper needs to be edited for spelling and grammar and I have listed some minor points below.<br>Response: We carefully checked the whole main text, supplementary notes and tables to improve the language.<br><br>2. Can the authors explain why they chose a male for the genome assembly? The homogametic sex is often selected for assembly in an effort to generate high enough coverage for the assembly of one of the sex chromosomes. If there is a reason, including oversight, I think this should be noted for subsequent groups interested in assembling non-model genomes.<br>Response: Only the male individuals can secrete the musk. One of the major aims for this genomic project is going to provide whole genome sequence to investigate potential pathway/regulation of musk secretion. Therefore, we chose to sequence and assemble a male individual.<br><br>3. L84-86: Awkward wording<br>Response: We have re-written the word as “In the last two centuries, hunting of all musk deer species significantly increased because of the commercially valuable of musk, which was an essential basis for perfume manufacture”.<br><br>4. L89: hyphen unnecessary<br>Response: Thanks, we deleted the hyphen.<br><br>5. L100-102: Please provide a reference supporting disease severity being exacerbated by inbreeding and lack of genetic diversity.<br>Response: We added two references.<br>1. Zhao K, Liu Y, Zhang X, et al. Detection and characterization of antibiotic-resistance genes in Arcanobacterium pyogenes strains from abscesses of forest musk deer. J Med Microbiol. 2011;60:1820-6.<br>2. Huang J, Li Y, Li P, et al. Genetic quality of the Miyaluo captive forest musk deer (Moschus berezovskii) population as assessed by microsatellite loci. Biochemical Systematics & Ecology, 2013;47(8):25-30.<br><br>6. L103: Please clarify what genetic health means<br>Response: We mean the genomic information could be useful for the genetic management and disease prevention of the captive forest musk deer. To avoid misunderstanding, we have re-written the sentence.<br><br>7. L107-108: Needs revision<br>Response: Thanks, it was a mistake, we already removed selection and gene enrichments based on editor and reviewers’ last comments. We have re-written this sentence.<br><br>8. L137: Is there a citation for the transcriptome data? Sun et al. generated transcriptomic data, and their analysis / story are relevant to this manuscript.<br>Response: The transcriptome data were used to evaluate the assembly and help the annotation. These data were uploaded to NCBI by Sichuan Agricultural University on July 2015. We did not find related publication. However, I contacted the author (submitter), and they said the paper had been published on Dec. 2017. They did not

use the SSR numbers within the paper, thus we could not find it. Now, we cited their publication (Xu et al., 2017; Line 104 of the main text). The new paper (Sun et al., 2018) was published on January 2018 by other Chinese group. Therefore, we could not use their new data. However, we cited Sun et al.'s paper at the Introduction Section.

9. L139-L141. Delete - let the reader decided, based on the statistics provided, if this is a high quality genome
Response: We deleted this sentence.

10. L178. WEGO is not defined.
Response: We added the explanation. It is Web Gene Ontology Annotation Plot.

11. L182. Avoid the use of and/or; or will suffice 99% of the time.
Response: Thanks, we only keep the "or" in the sentence.

12. L189. That is your entire list of TEs, so "such as" is not required.
Response: We replaced "such as " as "including".

13. L233. "China's ecology" should be written differently.
Response: Thanks, it was a mistake, we have re-written the words as "Chinese ecology".

14. L236: (E)ndangered - should be lower case.
Response: Thanks, we have re-written the word.

Reviewer #2

The authors addressed most of my concerns. The editors provided the link to the Gigascience repository with the data.

Remaining comments:

1. Unanswered question: the EVM usage is not specified, nor is it mentioned in the Sup Notes. As this merging step was the one that generated the final annotation, according to the source field of the gff file, it would be useful to describe it.
Response: We added the information for EVM in Supplementary Notes: "Finally, EVM was used to interpret all the above evidences, and the key parameters were as following: segmentSize = 1Mb, overlapSize = 20kb. The weight for de novo, homology and transcriptome-based gene predictions in EVM were set to 1, 5, and 10 respectively.".

2. Sequencing and filtering: was cutadapt used with the same parameters for regular PE and mate-pair libraries? I am not sure it should be. Please precise. Was NGSQCToolkit used after cutadapt? Isn't there some redundancy with its adapter trimming step?
Response: NGSQCToolkit could not remove the adapters. The sequencing company (Novogene, China) had all the libraries based on the manufacturer's protocol, thus Novogene had the adaptor information. They removed the adaptors and duplicate reads, then we ran NGSQCToolkit to further control the data quality. We added this explanation within the Supplementary Notes (section one).

3. 117: "A total of 407Gb of raw data were generated, after filtering out low quality, duplicate and adaptor polluted reads. Approximately 360Gb of high-quality reads were retained for genome assembly (Table 1)."

Powered by Editorial Manager® and ProduXion Manager® from Aries Systems Corporation

	407Gb *after* filtering? Why 360Gb then?<br>Response: Sorry, our sentences were not clean. The raw data is about 407 Gb, and the clean data is about 360 Gb. We have re-written the sentences. Now, it is: “A total of 407Gb of raw data were generated. After filtering out low quality, duplicates and adaptor polluted reads, about 360Gb of high-quality reads were retained for genome assembly”.<br><br>4. The Supplementary Notes should be improved and proofread. Examples:<br>p.1: "sequencing data quality control was guide by ", "he re", "base- calling"<br>p.2: "were mapping to musk deer genome"<br>p.3: "were then aligned" … "The script require"… "will concatenate" … "It finally produces" (check the tense)<br>Response: We carefully checked the whole supplementary notes and tables to improve the language.
**Additional Information:**	
**Question**	**Response**
Are you submitting this manuscript to a special series or article collection?	No
**Experimental design and statistics**<br><br>Full details of the experimental design and statistical methods used should be given in the Methods section, as detailed in our Minimum Standards Reporting Checklist. Information essential to interpreting the data presented should be made available in the figure legends.<br><br>Have you included all the information requested in your manuscript?	Yes
**Resources**<br><br>A description of all resources used, including antibodies, cell lines, animals and software tools, with enough information to allow them to be uniquely identified, should be included in the Methods section. Authors are strongly encouraged to cite Research Resource Identifiers (RRIDs) for antibodies, model organisms and tools, where possible.<br><br>Have you included the information requested as detailed in our Minimum Standards Reporting Checklist?	Yes
**Availability of data and materials**<br><br>All datasets and code on which the conclusions of the paper rely must be either included in your submission or deposited in publicly available repositories (where available and ethically	Yes

appropriate), referencing such data using a unique identifier in the references and in the “Availability of Data and Materials” section of your manuscript.<br><br>Have you have met the above requirement as detailed in our Minimum Standards Reporting Checklist?	

Manuscript Click here to download Manuscript MuskDeer26_3.docx

The draft genome sequence of forest musk deer (*Moschus berezovskii*)

Zhenxin Fan[1,†], Wujiao Li[1,†], Jiazheng Jin[1], Kai Cui[1], Chaochao Yan[1], Changjun Peng[1], Zuoyi Jian[1], Ping Bu[1], Megan Price[1], Xiuyue Zhang[1], Yongmei Shen[3], Jing Li[1], Wenhua Qi[2,*], Bisong Yue[1,*]

[1] Key Laboratory of Bioresources and Ecoenvironment (Ministry of Education), College of Life Sciences, Sichuan University, Chengdu 610064, People's Republic of China

[2] College of Life Science and Engineering, Chongqing Three Gorges University, Chongqing 404100, People's Republic of China

[3] Sichuan Engineering Research Center for Medicinal Animals, Xichang 615000, People's Republic of China

† Contributed equally to this work

* **Corresponding author:** Bisong Yue (bsyue@scu.edu.cn), and Wenhua Qi (wenhuaqi357@163.com)

Emails: zxfan@scu.edu.cn (Zhenxin Fan); hnnd059@gmail.com (Wujiao Li); wenhuaqi357@163.com (Wenhua Qi); jinjiazhengxiao@163.com (Jiazheng Jin); 2015222040118@stu.scu.edu.cn (Kai Cui); yccvican@gmail.com (Chaochao Yan); jj-5380682@163.com (Changjun Peng); jzuoyi@126.com (Zuoyi Jian); 757984931@qq.com (Ping Bu); meganprice@scu.edu.cn (Megan Price); zhangxy317@126.com (Xiuyue Zhang); Yongmei Shen (810316122@qq.com); Jing Li (ljtjf@126.com); bsyue@scu.edu.cn (Bisong Yue)

Abstract

Background: The forest musk deer, *Moschus berezovskii*, is one of seven musk deer (*Moschus* spp.) and is distributed in Southwest China. Akin to other musk deer, the forest musk deer has been traditionally, and is currently, hunted for its musk (i.e. global perfume industry). Considerable hunting pressure and habitat loss has caused significant population declines and therefore the Chinese government commenced captive breeding programs for musk harvesting in the 1950s. However, the prevalence of fatal diseases is considerably restricting population increases. Disease severity and extent is exacerbated by inbreeding and genetic diversity declines in captive musk deer populations. It is essential for the physical and genetic health of captive and wild forest musk deer populations to improve the knowledge of its immune system and genome. We have thus sequenced the whole genome of the forest musk deer, completed the genomic assembly and annotation, and performed preliminary bioinformatic analyses.

Findings: A total of 407 Gb raw reads from whole-genome sequencing was generated by the Illumina HiSeq 4000 platform. The final genome assembly is around 2.72 Gb, with a contig N50 length of 22.6 kb and a scaffold N50 length of 2.85 Mb. We identified 24,352 genes, and found 42.05% of the genome is composed of repetitive elements. We also detected 1,236 olfactory receptor genes. The genome-wide phylogenetic tree indicated that the forest musk deer was within the order Artiodactyla, and it appeared as the sister clade of four members of Bovidae. In total, 576 genes were under positive selection in the forest musk deer lineage.

Conclusions: We provide the first genome sequence and gene annotation for the forest musk deer. The availability of these resources will be very useful for the conservation and captive breeding for this endangered and economically important species, and for reconstructing the evolutionary history of the order Artiodactyla.

Keywords: Forest musk deer; whole genome sequencing; genome assembly; annotation; phylogeny

Data Description

1) Background

The seven musk deer species of the genus *Moschus* are endemic to Asia. They are currently listed under Appendix II in CITES and under Category I of the State Key Protected Wildlife List of China [1-3]. All musk deer species are considered as globally threatened, with six being listed as endangered and one as vulnerable by the IUCN [4]. *Moschus* is the only extant genus of Moschidae and musk deer are considered as primitive deer. The genus of musk deer is characterized by the musk secreted by the scent glands of adult males [5]. The forest musk deer (*Moschus berezovskii*) is one of the five recognized musk deer species of China and have historically been distributed in Southwest China [6,7]. The forest musk deer has been listed as globally endangered, as Critically Endangered on the 2015 China Red List, and is also on the State Key Protected Wildlife List of China [4].

Musk deer have been hunted for thousands of years, as the musk has been widely used in traditional Chinese medicines. In the last two centuries, hunting of all musk deer species significantly increased because of the commercial value of musk, which was an essential basis for perfume manufacture [5]. Since the 1950s, populations of forest musk deer have declined dramatically from poaching of deer for the musk pods (i.e. entire gland) and significant habitat destruction [3,6,8]. As a consequence, the Chinese government has encouraged musk using enterprises to participate in artificial breeding programs since the early 1950s [9]. The musk can be collected from male musk deer in these captive

populations without harvesting individuals, further enhancing the commercial and conservation value of captive populations.

The captive population of the forest musk deer is the largest among all the musk deer species [2,10]. The Miyaluo farming population in Sichuan Province (China) was one of the earliest established captive breeding populations. This population had grown rapidly to approximately 400 in 2010 [10]. However, the prevalence of fatal diseases is considerably restricting population increases [11]. Common diseases of forest musk deer in the Miyaluo population are dyspepsia, pneumonia, metritis, urinary stones and abscesses, with abscesses being one of the most prevalent causes of death [7]. Disease severity and extent is exacerbated by inbreeding and genetic diversity declines in this and other captive musk deer populations [7,10].

Although the transcriptomes of captive forest musk deer had been reported [12,13], there is no complete genome sequence, which is essential for the genetic management and disease prevention of captive and wild forest musk deer populations to improve knowledge of its immune system. We have thus sequenced the whole genome of the forest musk deer, subsequently completed the genomic assembly and annotation, and performed preliminary bioinformatic analyses, such as phylogenetic tree.

2) Sample information and sequencing

The thigh muscle sample was collected from a Miyaluo male forest musk deer that naturally died (Sichuan Province, China) in 2015. We extracted genomic DNA from the muscle sample using the Qiagen DNeasy Blood and Tissue Kit

(Qiagen, Valencia, USA) following the manufacturer's protocol. We constructed six different insert size libraries: 230bp, 500bp, 2kb, 5kb, 10kb, and 15kb. These libraries were sequenced by Illumina HiSeq 4000 platform at Novogene (Beijing, China). A total of 407Gb of raw data were generated. After filtering out low quality, duplicates and adaptors, about 360Gb of high-quality reads were retained for genome assembly (Table 1).

3) Genome assembly and evaluation

We use GCE (version 1.0) to performed k-mer (17-mer) analysis by short insert size library reads before assembly, and the forest musk deer genome size was estimated to be 2.95Gb (Figure S1). The assembly was first generated by SOAPdenovo2 (SOAPdenovo2, RRID:SCR_014986) [14] with the parameters set as "all -d 2 –M 2 –k 35". Intra-scaffold gaps were filled using Gapcloser (version 1.12) with reads from 230bp and 500bp libraries, and then SSPACE version 3.0 (SSPACE, RRID:SCR_005056) [15] was used to build super-scaffolds. After scaffolding by SSPACE, we used Gapcloser again to fill gaps. Finally we obtained the forest musk deer genome with a size of 2.72Gb (all the sequences with length shorter than 300bp were removed) with 125.7Mb gap sequences unsolved. The N50s of contigs and scaffolds of forest musk deer genome were 22.6kb and 2.85Mb, respectively (Table 2).

We used BUSCO version 3.0 (BUSCO, RRID:SCR_015008) to evaluate the genome complement. BUSCO results showed that 84.5% of the eukaryotic single-copy genes were captured (Table S1). Furthermore, we downloaded musk gland RNA-seq data (SRA accession: SRR2098995 and SRR2098996) of forest musk

deer from NCBI to evaluate the assembly [13]. We found that 99.3% of the total PE reads could be aligned (92.73% aligned concordantly) to the assembled forest musk deer genome by Bowtie2 (version 2.2.5) [16].

4) Annotation

We combined the *de novo*, homology-based and transcriptome-based prediction to identify protein-coding genes in the forest musk deer genome. The software Augustus version 3.2.1 (Augustus: Gene Prediction, RRID:SCR_008417) [17] was used for *de novo* prediction based on the parameter trained for forest musk deer. For homology prediction, protein sequences from four mammals (human, pig, sheep and cattle) were analyzed with TBLASTN (BLAST version 2.2.26) against forest musk deer genome. Potential gene regions were joined by SOLAR (version 0.9.6) [18], and the coding sequence with 500bp flanking sequence were cut down and re-aligned by GeneWise (GeneWise, RRID:SCR_015054) ,version 2.4.1 with parameters "- sum - genesf -gff" [19]. For transcriptome-based prediction, musk gland RNA-seq data were assembled by Trinity (Trinity, RRID:SCR_013048) with genome guide and *de novo* mode, respectively. The gene structures were obtained by PASA pipeline (version 2.0.2) [20]. We used EVM (version 1.1.1) to integrate the above evidence and obtained a consensus gene set [21]. Apollo (version 1.11.6) was performed to manually inspect gene structure in scaffolds of sizes above 1Mb to gain a more accurate gene structure. We consequently found a total of 24,352 genes predicted to be present in the forest musk deer genome. We also provided the length of genes in Table S2.

Functional annotation of forest musk deer genes was undertaken based on the best match derived from the alignments to proteins annotated in Swiss-Prot and TrEMBL databases [22]. Functional annotation used BlastP tools with the same E-value cut-off of 1E-5. We also annotated proteins against the NCBI non-redundant (nr) protein database. The outputs of blast searching against the NCBI nr protein database were imported into BLAST2GO (B2G4PIPE v2.5) for Gene Ontology (GO) [23] term mapping. Term mapping used annotated motifs and domains using InterProScan (InterProScan, RRID:SCR_005829), interproscan-5.18-57.0, [24] by searching against publicly available databases. To find the best match for each gene, KEGG pathway maps were used by searching KEGG databases [25] through the KEGG Automatic Annotation Server (KAAS) using the bi-directional best hit (BBH) method. In total, 23,023 out of 24,352 (94.5%) protein-coding genes were searched within the publicly available functional databases of TrEMBL, Swiss-Prot, Interpro, GO and KEGG. Of which, 22,696 (93.20% TrEMBL), 18,771 (77.08% Swiss-Prot), 22,221 (91.12% Interpro), 15,736 (64.62% GO) and 10,846 (44.54% KEGG) genes showed significant similarity matches (Figure 1; Table 3). The functional comparisons with two closely related species (cattle and sheep) for GO classification were submitted to the Web Gene Ontology Annotation Plot (WEGO) [26] (Figure S2).

5) Repetitive sequences and transposable elements

Transposable elements (TEs) and other repeats make up a substantial fraction of mammalian genomes and contribute to gene or genome evolution [27]. The TE content, type, copy number, subfamily, and divergence rate were investigated in

the forest musk deer genome based on two strategies: the library based strategy of RepeatMasker (RepeatMasker, RRID:SCR_012954) [28] and the *de novo* based strategy of RepeatScout (RepeatScout, RRID:SCR_014653) [29]. The forest musk deer genome has large numbers of TEs, comprising 42.05% of the genome (Table S3), which is similar to those of cattle (46.5%) [27] and goats (42.2%) [30]. The 23 different types of TEs have been grouped for the four different types of TEs, including DNA transposons, LTR, LINE, and SINE retrotransposons (Figure S3). The LINEs were the most common repeats in forest musk deer genome; followed by SINEs > LTR > DNA. We also analyzed the degree of divergence for each type of TE in the forest musk deer genome. We found there was a recent burst activity involving LINE transposons and a second, older burst activity of LTR and DNA transposons (Figure S3).

A total of 542,135 microsatellites (simple sequence repeats, SSRs) were identified by software MSDB [31] in the forest musk deer genome assembly (Table S4), which accounted for 0.45% of its whole genome length. Mononucleotide SSRs were the most abundant category, accounting for 41.75% of all of the SSRs; followed by followed by: di- > tri- > tetra- > penta- > hexa nucleotide SSRs (Table S4).

6) Gene families

To estimate species-specific and shared genes in the forest musk deer in comparison to ten mammal species, we used orthoMCL [32] to define the orthologous genes. We downloaded the genomes and gene annotations of the ten additional species (human, horse, dog, cattle, mouse, yak, sheep, Tibetan

antelope, alpaca, and pig) from Ensembl [33] or NCBI (Table S5). In total, we identified 18,855 homologous gene families shared by forest musk deer and the ten additional species, 221 gene families that were specific to forest musk deer, and 2,003 gene families found in the ten additional species but not in the forest musk deer (Figure S4). In addition, we found 5,372 one-to-one orthologous genes within forest musk deer and other ten species, which was used in phylogenetic analyses. In addition, we detected olfactory receptor (OR) genes in the forest musk deer genome by orfam (https://github.com/jianzuoyi/orfam) since they formed the largest gene family in mammalian genomes [34]. In total, we identified 1,236 OR genes, which included 866 intact, 266 pseduogenes, and 104 truncated genes.

7) Phylogenetic analysis

We constructed the phylogenetic trees based on Bayesian inference (BI) [35] and maximum likelihood (ML) [36,37] analyses with the discovered 5,372 one-to-one orthologous genes (Supplementary notes). All the different methods generated the same topology and obtained the well-supported phylogenetic tree (Figure 2). The forest musk deer was within the suborder Ruminantia, order Artiodactyla, and it appeared as the sister clade of four members of family Bovidae (sheep, yak, cattle, and Tibetan antelope). Since we do not have high quality genome sequences for species within family Cervidae, the relationship between Moschidae, Cervidae, and Bovidae at the genomic level is tentative and needs further investigation.

Conclusions

Here, we report the first draft genome assembly of the forest musk deer genome, a species that is of particular importance to Chinese ecology, biodiversity conservation, economy, and medicine. The availability of the genome and these results will be very useful for the conservation and captive breeding of this endangered and economically important species, and for reconstructing the evolutionary history of the order Artiodactyla.

Funding

This work was supported by National Key Program of Research and Development, Ministry of Science and Technology (2016YFC0503200), and National Natural Science Foundation of China (31702032).

Availability of supporting data

The DNA sequencing data have been deposited into the NCBI Sequence Read Archive (SRA) under the ID PRJNA317652. Other supporting data, including the assembled genome, gene annotations and BUSCO results, are available via the GigaScience repository, GigaDB [38].

Conflicts of interest

The authors declare that they have no competing interests.

Author's contributions

Z.F., X.Z., J.L., and B.Y. designed and supervised the project. Z.F., W.L., C.Y., J.J., C.P., J.Y., P.B., Y.S., and K.C. performed the bioinformatics analyses. M.P. revised the manuscript. Z.F. and B.Y. wrote the manuscript.

Figure Legend

Figure 1 Functional annotation statistics. Venn diagram illustrating distribution of high-score matches of the functional annotation in forest musk deer genome from five public databases.

Figure 2 Genome wide phylogenetic trees. We constructed the phylogenetic trees based on Bayesian inference and maximum likelihood analyses with 5,372 one-to-one orthologous genes between the forest musk deer and ten other species.

Figure S1 K-mer (k=17) distributions in forest musk deer genome.

Figure S2 GO comparative analysis and functional classification between forest musk deer, sheep and cattle.

Figure S3 Distribution of divergence of each type of TEs in forest musk deer genome. The divergence rate was calculated between the identified TE elements in the genome and the consensus sequence in the TE library used. SINEs: Short interspersed elements. LINEs: Long interspersed elements. LTR: Long terminal repeat retrotransposon.

Figure S4 Protein orthology comparison between different genomes. There were forest musk deer (*Moschus bweezovskii*), cattle (*Bos taurus*), yak (*Bos grunniens*), sheep (*Ovis aries*), Tibetan antelope (*Pantholops hodgsonii*), alpaca (*Vicugna pacos*), and pig (*Sus scrofa*), which representing Artiodactyla; human (*Homo sapiens*, Primates), horse (*Equus caballus*, Perissodactyla), and dog (*Canis lupus familiaris*, Carnivora), mouse (*Mus musculus*, Rodentia). For each animal, proteins were represented by bars and were classified based on orthoMCL analysis. Single_copy (green) included the common orthologs with the same number of copies in different species; Multi_copy (red) included the common orthologs with different copy numbers in different species; Unique (magenta) included the orthologs that were only in one species; Unclustered genes (yellow) included the genes that could not be clustered into known gene families; Other (blue) included the genes that could be clustered into known gene families, but were not belonged to Single_copy, Multi_copy or Unique.

References

1. Cobert GB, Hill JE. The mammals of the Indomalaysia region: a systematic review. London (UK): Natural History Museum Publications Oxford University Press. 1992.

2. Wu J, Wang W. The Musk Deer of China. The China Forestry Publishing House, Beijing. 2006.

3. Sheng H, Liu Z. The Musk Deer in China. The Shanghai Scientific & Technical Publishers, Shanghai. 2007.

4. IUCN. The IUCN Red List of Threatened Species. 2017 (http://www.iucnredlist.org/)

5. Green MJB. The distribution, status and conservation of the Himalayan musk deer (Moschus chrysogaster). Biol. Conserv. 1986;35, 347–75.

6. Sheng H. Genus Moschus in China. In: Wang, S. (Ed.), China Red Data Book of Endangered Animals. Science Press, Beijing. 1998.

7. Zhao K, Liu Y, Zhang X, et al. Detection and characterization of antibiotic-resistance genes in Arcanobacterium pyogenes strains from abscesses of forest musk deer. J Med Microbiol. 2011;60:1820-6.

8. Yang Q, Meng X, Xia L, Feng Z. Conservation status and causes of decline of musk deer (Moschus spp.) in China. Biol. Conserv. 2003;109, 333–342.

9. Peng H, Liu S, Zou F, Zeng B, Yue B. Genetic diversity of captive forest musk deer (Moschus berezovskii) inferred from the mitochondrial DNA control region. Anim Genet. 2009;40(1):65-72.

10. Huang J, Li Y, Li P, et al. Genetic quality of the Miyaluo captive forest musk deer (Moschus berezovskii) population as assessed by microsatellite loci. Biochemical Systematics & Ecology, 2013;47(8):25-30.

11. Lu X, Qiao J, Wu X, Su L. A review of mainly affected on musk-deer diseases: purulent, respiratory system and parasitic diseases. J Economic Anim 2009, 13, 104–107.

12. Xu Z, Jie H, Chen B, et al. Illumina-based de novo transcriptome sequencing and analysis of Chinese forest musk deer. J Genet. 2017;96(6):1033-1040.

13. Sun X, Cai R, Jin X, et al. Blood transcriptomics of captive forest musk deer (Moschus berezovskii) and possible associations with the immune response to abscesses. Sci Rep. 2018;8(1):599.

14. Luo R, Liu B, Xie Y, et al. SOAPdenovo2: an empirically improved memory-efficient short-read de novo assembler. Gigascience 2012;1(1):18.

15. Boetzer M, Henkel CV, Jansen HJ, Butler D, Pirovano W. Scaffolding pre-assembled contigs using SSPACE. Bioinformatics. 2011;27(4):578-9.

16. Langmead B, Salzberg SL. Fast gapped-read alignment with Bowtie 2. Nat Methods. 2012;9(4):357-9.

17. Stanke M, Diekhans M, Baertsch R, Haussler D. Using native and syntenically mapped cDNA alignments to improve de novo gene finding. Bioinformatics. 2008;24(5):637-44.

18. Yu XJ, Zheng HK, Wang J, Wang W, Su B. Detecting lineage-specific adaptive evolution of brain-expressed genes in human using rhesus macaque as outgroup. Genomics. 2006;88(6):745-751.

19. Birney E, Clamp M, Durbin R. GeneWise and Genomewise. Genome Res. 2004;14(5):988-95.

20. Haas BJ, Delcher AL, Mount SM, et al. Improving the Arabidopsis genome annotation using maximal transcript alignment assemblies. Nucleic Acids Res. 2003;31(19):5654-66.

21. Haas BJ, Salzberg SL, Zhu W, Pertea M, Allen JE, Orvis J, White O, Buell CR, Wortman JR. Automated eukaryotic gene structure annotation using EVidenceModeler and the Program to Assemble Spliced Alignments. Genome Biol. 2008;9(1):R7.

22. Boeckmann B, Bairoch A, Apweiler R, et al. The SWISS-PROT protein knowledgebase and its supplement TrEMBL in 2003. Nucleic Acids Res. 2003;31(1):365-70.

23. Gene Ontology Consortium. Gene Ontology annotations and resources. Nucleic Acids Res. 2013;41(Database issue):D530-5.

24. Hunter S, Apweiler R, Attwood TK, et al. InterPro: the integrative protein signature database. Nucleic Acids Res. 2009;37(Database issue):D211-5.

25. Kanehisa M, Goto S. KEGG: kyoto encyclopedia of genes and genomes. Nucleic Acids Res. 2000;28(1):27-30.

26. Ye J, Fang L, Zheng H, et al. WEGO: a web tool for plotting GO annotations. Nucleic Acids Res. 2006;34(Web Server issue):W293-7.

27. Adelson DL, Raison JM, Edgar RC. Characterization and distribution of retrotransposons and simple sequence repeats in the bovine genome. Proc Natl Acad Sci U S A. 2009;106(31):12855-60.

28. Smit AFA, Hubley R, Green P. 2016. RepeatMasker website and server[CP/OL]. (2016-9-12)[2016-10-15]. http://www.repeatmasker.org/.

29. Price AL, Jones NC, Pevzner PA. De novo identification of repeat families in large genomes. Bioinformatics. 2005;21 Suppl 1:i351-8.

30. Dong Y, Xie M, Jiang Y, et al. Sequencing and automated whole-genome optical mapping of the genome of a domestic goat (Capra hircus). Nat Biotechnol. 2013;31(2):135-41.

31. Du L, Li Y, Zhang X, Yue B. MSDB: a user-friendly program for reporting distribution and building databases of microsatellites from genome sequences. J Hered. 2013;104(1):154-7.

32. Li L, Stoeckert CJ Jr, Roos DS. OrthoMCL: identification of ortholog groups for eukaryotic genomes. Genome Res. 2003;13(9):2178-89.

33. Yates A, Akanni W, Amode MR et al. Ensembl 2016. Nucleic Acids Res. 2016;44(D1):D710–16.

34. Firestein S. How the olfactory system makes sense of scents. Nature. 2001;413(6852):211-8.

35. Ronquist F, Huelsenbeck JP. MrBayes 3: Bayesian phylogenetic inference under mixed models. Bioinformatics. 2003;19(12):1572-4.

36. Guindon S, Dufayard JF, Lefort V, et al. New algorithms and methods to estimate maximum-likelihood phylogenies: assessing the performance of PhyML 3.0. Syst Biol. 2010;59(3):307-21.

37. Stamatakis A, Hoover P, Rougemont J. A rapid bootstrap algorithm for the RAxML Web servers. Syst Biol. 2008;57(5):758-71.

38. Fan Z, Li W, Jin J, Cui K, Yan C, Peng C, et al Supporting data for "The draft genome sequence of forest musk deer (Moschus berezovskii)" *GigaScience* Database 2018. http://dx.doi.org/10.5524/100411

 Click here to download Table Table 1_sequencing.docx

Table 1 Genome sequencing information.

Insert size (bp)	Read length (bp)	Raw data		Clean data	
		Total bases (Gb)	Sequencing depth (x)	Total bases (Gb)	Sequencing depth (x)
230	125	135.76	46.02	125.96	42.70
500	125	102.51	34.75	88.52	30.01
2,000	125	59.0	20.00	50.16	17.00
5,000	125	51.57	17.48	46.39	15.73
10,000	125	28.16	9.55	24.67	8.36
15,000	125	30.34	10.28	28.14	9.54
Total		407.34	138.08	363.84	123.34

Note: Genome size is 2.95Gb.

 Click here to download Table Table 2_new.docx

Table 2 Statistics of the final assembly of forest musk deer genome.

Genome assembly	Numbers
Contig N50 (Kb)	22.6
Scaffold N50 (Mb)	2.85
Longest scaffold (Mb)	18.69
Scaffold number	79,206
GC content	40%
Total length (Gb)	2.72

Table 3 Click here to download Table Table 3_FunctionalAnnotation.docx

Table 3 Functional annotation statistics of the forest musk deer genome by various methods.

	Database	Number	Percent (%)
Total		24,352	100.00
	Swissprot	18,771	77.08
	TrEMBL	22,696	93.20
Annotated	KEGG	10,846	44.54
	Interpro	22,221	91.12
	GO (blast2go)	15,736	64.62
	GO (Interproscan)	14,815	60.84
Un-annotated		1,329	5.77

Figure 1

Click here to download Figure Figure 1_annotation.tif

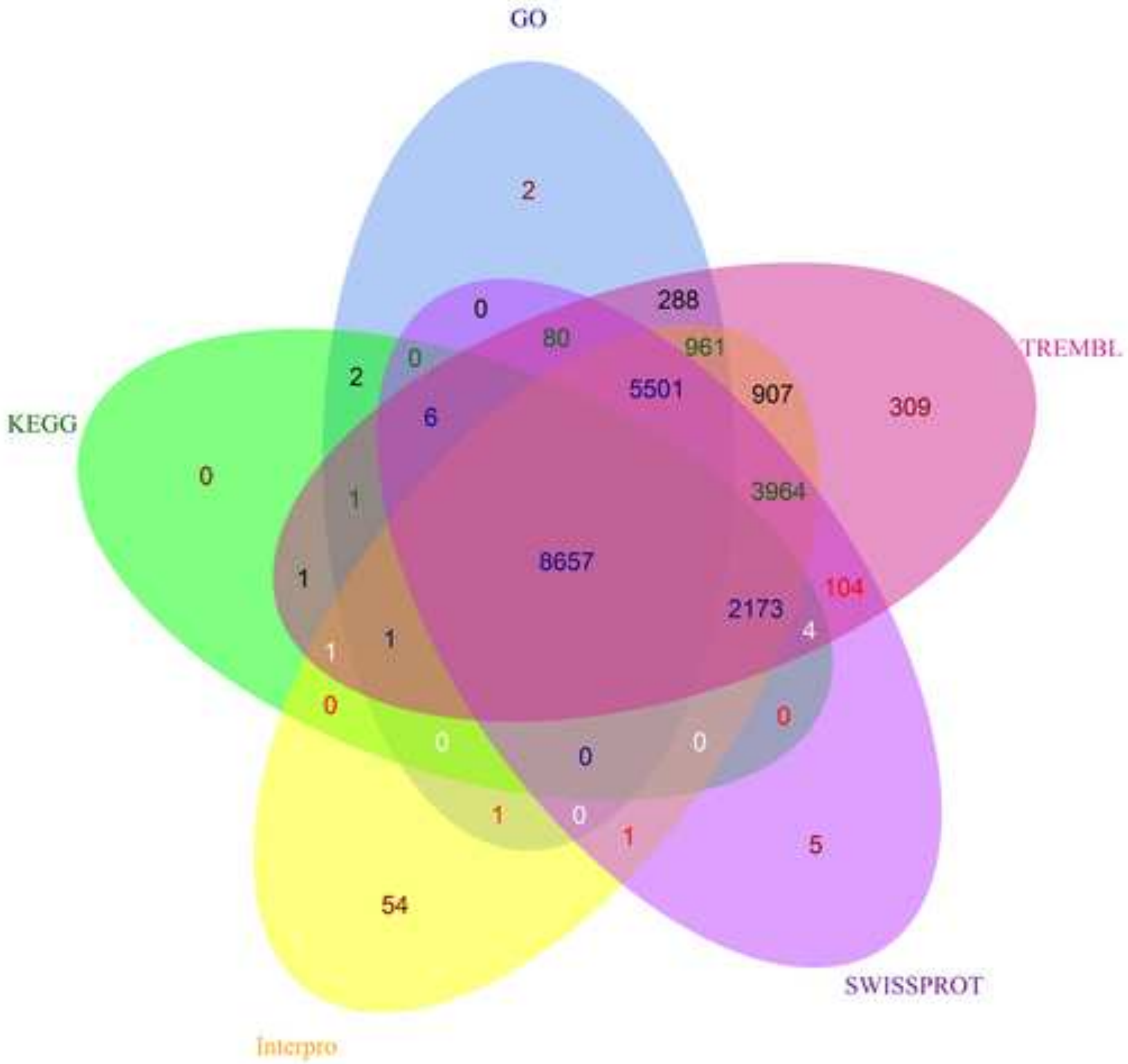

Figure 2

Click here to download Figure Figure 2_tree.tif

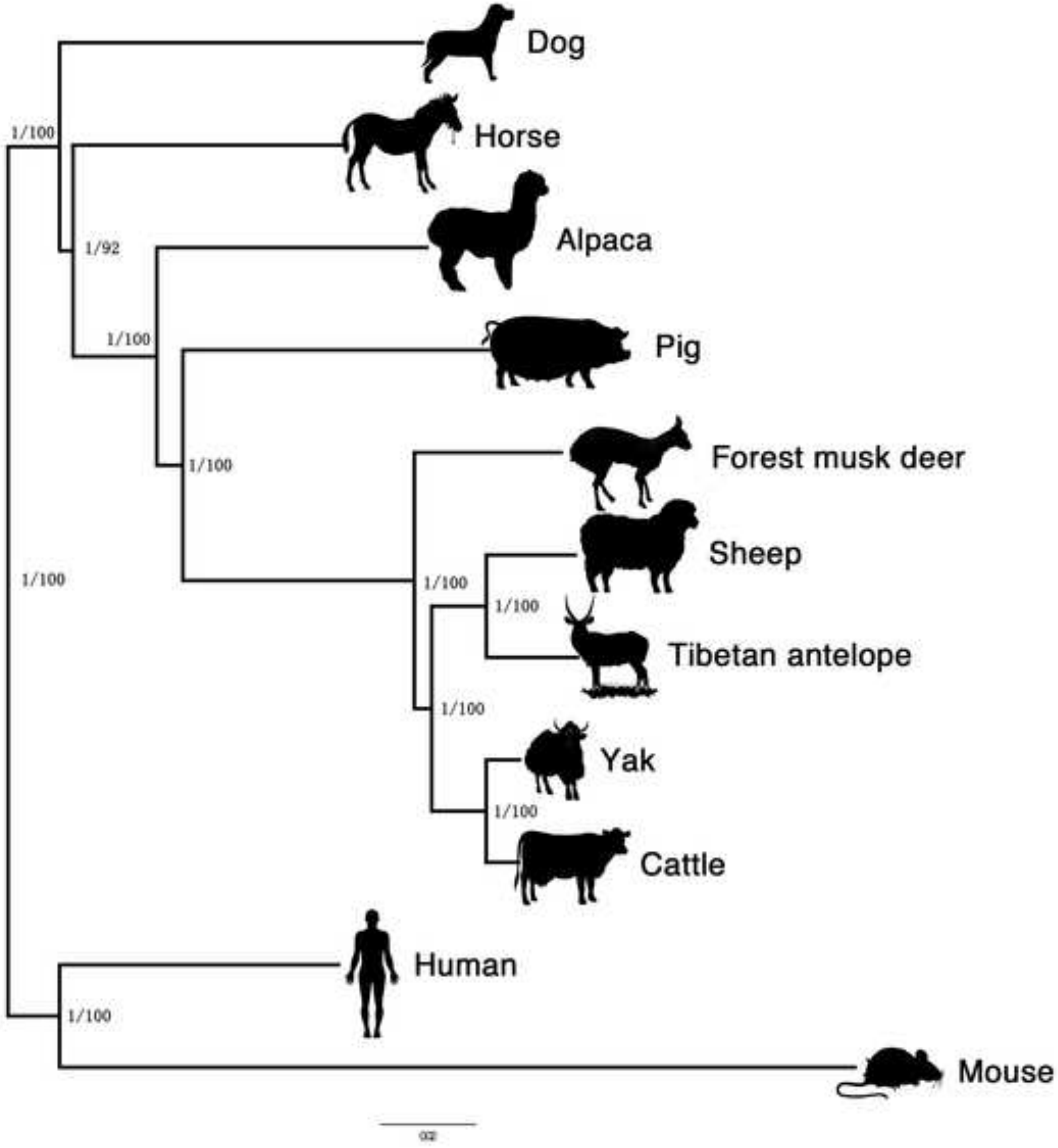

Supplementary Figure S1

Click here to access/download
Supplementary Material
Figure S1_kmer.tif

Supplementary Figure S2

Click here to access/download
Supplementary Material
Figure S2_wego.pdf

Supplementary Figure S3

Click here to access/download
Supplementary Material
Figure S3_TE_type.tif

Supplementary Figure S4

Click here to access/download
Supplementary Material
Figure S4_OrthologousGene.tif

Supplementary Table S1

Click here to access/download
Supplementary Material
Table S1_BUSCO.docx

Supplementary Table S2

Click here to access/download
Supplementary Material
Table S2_new.docx

Supplementary Table S3

Click here to access/download
Supplementary Material
Table S3_repeat.docx

Supplementary Table S4

Click here to access/download
Supplementary Material
Table S4_SSR.docx

Supplementary Table S5

Click here to access/download
Supplementary Material
Table S5_Orthologous.xlsx

Supplementary Notes

Click here to access/download
Supplementary Material
SuppNote_R2_0207.docx
