# Supplementary material for: The draft genome sequence of forest musk deer (Moschus berezovskii)
Source: Gigascience. 2018 Apr 9;7(4):giy038. doi: 10.1093/gigascience/giy038 (PMC5906906; doi:10.1093/gigascience/giy038)
Supplement: Supplemental material [file giy038_supp.zip › SuppNote_R2_0207.docx]

**The draft genome sequence of forest musk deer (*Moschus berezovskii*)**

**Supplementary Notes**

**1. Sequencing and Filtering Raw Data**

The thigh muscle sample was collected from a male forest musk deer that naturally died (Miyaluo, Sichuan Province, China) in 2015. DNA was isolated from the muscle using the Qiagen Dneasy Blood and Tissue Kit according to manufacturer’s instructions. Short insert size libraries (230bp and 500bp) and mate-pair (2kb, 5kb, 10kb, and 15kb) DNA libraries were constructed. All the libraries were sequenced by Illumina HiSeq 4000 platform at Novogene (Beijing, China).

Adaptor sequences were trimmed by cutadapt (v1.9.1) by Novogene (China), and then the low quality sequences were filtered by NGSQCToolkit (v2.3). The data quality control was applied based on the following criteria:

(1) Remove reads that contained ambiguous character N more than 10% of the total length of each read.

(2) Remove low quality reads that have more than 70% bases with quality score less than Q20 for short insert size libraries. Quality score was the error probability of base calling. For example, Q20 was defined as an error probability of 0.01.

(3) For short insert libraries, reads with adapter contamination were removed, and for mate-pair reads, adapter sequences were trimmed off.

(4) Filter PCR duplicates. If read one and read two of two paired end (PE) reads were totally identical, these reads were considered as duplicates.

**2. Kmer Analysis**

The kmer frequencies along its depth followed a Poisson distribution. We used GCE (v1.0) to performed 17-mer analysis based on the clean data of PE reads from the 230bp insert size library. The 17-mer frequency distribution was plotted in Supplementary Figure S1. The forest musk deer genome size was estimated to be 2.95Gb.

**3. Genome Assembly**

First, the forest musk deer genome was assembled with SOAPdenovo2 (parameters ”-all -d 2 –M 2 –k 35 -F). Then, we used paired-end information to fill gaps by Gapcloser (v1.12). The results showed that 66.3% gaps were filled, which was 64.8% of the total gap length.

Next, SSPACE (v3.0, parameters: ”-v 1 –S”) was performed to build super-scaffold. The scaffold numbers were decreased from 418,429 to 399,682, and the scaffold N50 increased from 1.07Mb to 2.79Mb. Subsequently, Gapcloser was used to fill gap again, and about 13% gaps were filled, which was 25% of the total gap length (168Mb).

Finally, the contig N50 and scaffold N50 of the draft genome (sequence length>= 300bp) reached 22.6kb and 2.85Mb, respectively.

**4. Protein-coding Gene Prediction**

We combined the *de novo*, homology-based and transcriptome-based prediction to identify protein-coding genes in the forest musk deer genome. We used some protein sequences of *Bos taurus* to train the model for forest musk deer by Augustus (version 3.0.3), and then the model was applied in Augustus to perform *de novo* gene prediction for forest musk deer genome. Homolog proteins of four mammals (human, pig, sheep and cattle) were mapped to forest musk deer genome by tBLASTn (version 2.2.26, with parameters:”-e 1e-5”). Potential gene regions were joined by SOLAR (v0.9.6), the coding sequence with 500bp flanking sequences were extracted. The extracted sequence as well as its query protein were filtered by identity and coverage criteria, and then were passed to GeneWise (version 2.4.1, parameters “- sum - genesf - gff”) to refine the spliced alignments. For transcriptome-based gene prediction, to obtain more gene structure information, genome guide and *de novo* modes were applied to assemble musk gland RNA-seq data in PASA pipeline (version 2.0.2). Finally, EVM was used to interpret all the above evidences, and the key parameters were as following: segmentSize = 1Mb, overlapSize = 20kb. The weight for de novo, homology and transcriptome-based gene predictions in EVM were set to 1, 5, and 10 respectively.

**5. Phylogenetic Tree**

A total of 5,372 orthologous genes that shared by the 11 studied genomes were identified by OrthoMCL [3]. These gene sequences were aligned in software PRANK [4]. Low quality alignment blocks of gene sequences were removed by home-made R script [5], which imported alignment as MultipleAlignment object and then masked three-sites blocks with gaps over 30% of all bases. Aligned DNA genes as well as their translated AA sequences were then concatenated one with another one using a home-made python script called *concatenate_mass.py.* The script concatenated all fasta files (each file had an orthologous gene from the 11 studied species) into one. Finally, it generated a large concatenated alignment of 11 sequences with 8,283,057bp in length.

Trimal v1.4.rev15 [6] was implied to export both nexus and phylip formats of the concatenated DNA and AA sequences. The best-fit models of all three-codon partition regions were GTR+I+G, which were estimated in modeltest 3.7 [7,8]. Partitioned Bayes inference was performed in MrBayes 3.2 [9]. Two separate runs were performed with four Markov chains, each run for 2, 000, 000 generations and sampled every 100 generations. The consensus tree was calculated after omitting the first 25% trees as burn-in.

Maximum likelihood (ML) was analyzed in PhyML-mpi v20141029 [10] and RAxML version 8.2.8 [11,12]. The substitution model was set as GTR, and the tree topology search was set as BEST (best of NNI and SPR search). Four relative substitution rate categories were run for 1,000 bootstrap replicates to determine the support for the best tree. We also performed RAxML method with GTR+GAMMA model and 1,000 bootstrap tests to reconstruct the ML phylogenetic tree. All the above runs yielded a consensus tree (Figure 2). The same phylogenetic protocol was applied to construct combined AA sequences to confirm the DNA sequences results. The trees based on the AA sequences had the same topology of that of DNA sequences (not shown).

**Reference:**

1. Stanke M, Diekhans M, Baertsch R, Haussler D (2008) Using native and syntenically mapped cDNA alignments to improve de novo gene finding. Bioinformatics 24(5):637-44.

2. Birney E, Clamp M, Durbin R. (2004) GeneWise and Genomewise. Genome Res. 14(5):988-95.

3. Li L, Jr SC, Roos DS (2003) OrthoMCL: identification of ortholog groups for eukaryotic genomes. Genome Research 13: 2178-2189.

4. Löytynoja A, Goldman N (2009) webPRANK: a phylogeny-aware multiple sequence aligner with interactive alignment browser. Bmc Bioinformatics 11: : 579.

5. Gentleman RC, Carey VJ, Bates DM, Bolstad B, Dettling M, et al. (2004) Bioconductor: open software development for computational biology and bioinformatics. Genome Biology 5: R80.

6. Capella-Gutiérrez S, Silla-Martínez JM, Gabaldón T (2009) trimAl: a tool for automated alignment trimming in large-scale phylogenetic analyses. Bioinformatics 25: 1972-1973.

7. Posada D, Crandall KA (1998) MODELTEST: testing the model of DNA substitution. Bioinformatics 14: 817-818.

8. Wilgenbusch JC, Swofford D (2003) Inferring Evolutionary Trees with PAUP*. Current protocols in bioinformatics/editoral board, Andreas D Baxevanis, Chapter 6: 6.4.1-6.4.28.

9. Ronquist F, Huelsenbeck JP (2003) MrBayes 3: Bayesian phylogenetic inference under mixed models. Bioinformatics 19: 1572-1574.

10. Guindon S, Dufayard JF, Lefort V, Anisimova M, Hordijk W, et al. (2010) New algorithms and methods to estimate maximum-likelihood phylogenies: assessing the performance of PhyML 3.0. Systematic Biology 59: 307-321.

11. Stamatakis A, Hoover P, Rougemont J (2008) A Rapid Bootstrap Algorithm for the RAxML Web Servers. Systematic Biology 57: 758-771(714).

12. Stamatakis A (2014) RAxML Version 8: A tool for Phylogenetic Analysis and Post-Analysis of Large Phylogenies. Bioinformatics 30: 1312-1313.
