# Supplementary material for: The draft genome sequence of forest musk deer (Moschus berezovskii)
Source: Gigascience. 2018 Apr 9;7(4):giy038. doi: 10.1093/gigascience/giy038 (PMC5906906; doi:10.1093/gigascience/giy038)
Supplement: Supplemental material [file giy038_supp.zip › Table S1_BUSCO.docx]

Table S1 Statistics of the completeness of the genome based on BUSCO benchmark

| BUSCO benchmark | Number | | Percentage |
| --- | --- | --- | --- |
| Complete Single-Copy BUSCOs | 256 | | 84.5 |
| Complete Duplicated BUSCOs | 21 | | 6.9 |
| Fragmented BUSCOs | 7 | | 2.30 |
| Missing BUSCOs | 19 | | 6.3 |
| Total BUSCO groups searched | 303 |  | |
