# Supplementary material for: The draft genome sequence of forest musk deer (Moschus berezovskii)
Source: Gigascience. 2018 Apr 9;7(4):giy038. doi: 10.1093/gigascience/giy038 (PMC5906906; doi:10.1093/gigascience/giy038)
Supplement: Supplemental material [file giy038_supp.zip › Table S2_new.docx]

|  | Gene length | CDS length | Number of exons |
| --- | --- | --- | --- |
| Minimum | 150 bp | 9 bp | 1 |
| Maximum | 1,260,628 bp | 101,907 bp | 304 |
| Mean | 39,993 bp | 1,419 bp | 8 |
| Median | 16,038 bp | 978 bp | 5 |

Table S2 Statistics of gene structure annotations.
