# Supplementary material for: The draft genome sequence of forest musk deer (Moschus berezovskii)
Source: Gigascience. 2018 Apr 9;7(4):giy038. doi: 10.1093/gigascience/giy038 (PMC5906906; doi:10.1093/gigascience/giy038)
Supplement: Supplemental material [file giy038_supp.zip › Table S3_repeat.docx]

Table S3 Statistics of repeat elements in forest musk deer genome annotated by RepeatMasker

| Classes of repeats | Subfamilies | Number of elements | Length occupied (bp) | Percentage of genome (%) |
| --- | --- | --- | --- | --- |
| SINEs |  | 2,464,267 | 315,055,649 | 11.35 |
|  | Alu/B1 | 46 | 4,898 | 0 |
|  | MIRs | 649,697 | 64,433,866 | 2.32 |
| LINEs |  | 1,684,768 | 655,461,406 | 23.62 |
|  | LINE1 | 720168 | 290,706,330 | 10.48 |
|  | LINE2 | 260,620 | 61,803,972 | 2.23 |
|  | L3/CR1 | 36,064 | 7,116,473 | 0.26 |
|  | RTE | 666,991 | 295,703,235 | 10.66 |
| LTR |  | 455,253 | 130,464,999 | 4.7 |
|  | ERVL | 84,294 | 31,025,841 | 1.12 |
|  | ERVL-MaLRs | 132,805 | 40,599,142 | 1.46 |
|  | ERV_classI | 113,918 | 44,496,456 | 1.6 |
|  | ERV_classII | 105,189 | 9,916,238 | 0.36 |
| DNA |  | 333,591 | 65,031,299 | 2.34 |
|  | hAT-Charlie | 184,355 | 32,818,790 | 1.18 |
|  | TcMar-Tigger | 57,131 | 14,350,100 | 0.52 |
| Total interspersed repeats | |  | 1,166,927,756 | 42.05 |
