# Supplementary material for: The draft genome sequence of forest musk deer (Moschus berezovskii)
Source: Gigascience. 2018 Apr 9;7(4):giy038. doi: 10.1093/gigascience/giy038 (PMC5906906; doi:10.1093/gigascience/giy038)
Supplement: Supplemental material [file giy038_supp.zip › Table S4_SSR.docx]

Table S4 Statistics of SSRs in the forest musk deer genome.

| Type | Mono- | Di- | Tri- | Tetra- | Penta- | Hexa- |
| --- | --- | --- | --- | --- | --- | --- |
| Number of SSRs | 226,318 | 148,175 | 122,105 | 39,977 | 4,962 | 598 |
| Total length of SSRs (bp) | 3,192,531 | 2,719,558 | 2,077,536 | 678,452 | 106,801 | 15,420 |
| Relative abundance (Number/Mb) | 81.56 | 53.40 | 44.00 | 14.41 | 1.82 | 0.22 |
| SSRs content (%) | 41.75 | 27.33 | 22.52 | 7.37 | 0.92 | 0.11 |
