# Supplementary figures and images for: The draft genome sequence of forest musk deer (Moschus berezovskii)
Source: Gigascience. 2018 Apr 9;7(4):giy038. doi: 10.1093/gigascience/giy038 (PMC5906906; doi:10.1093/gigascience/giy038)

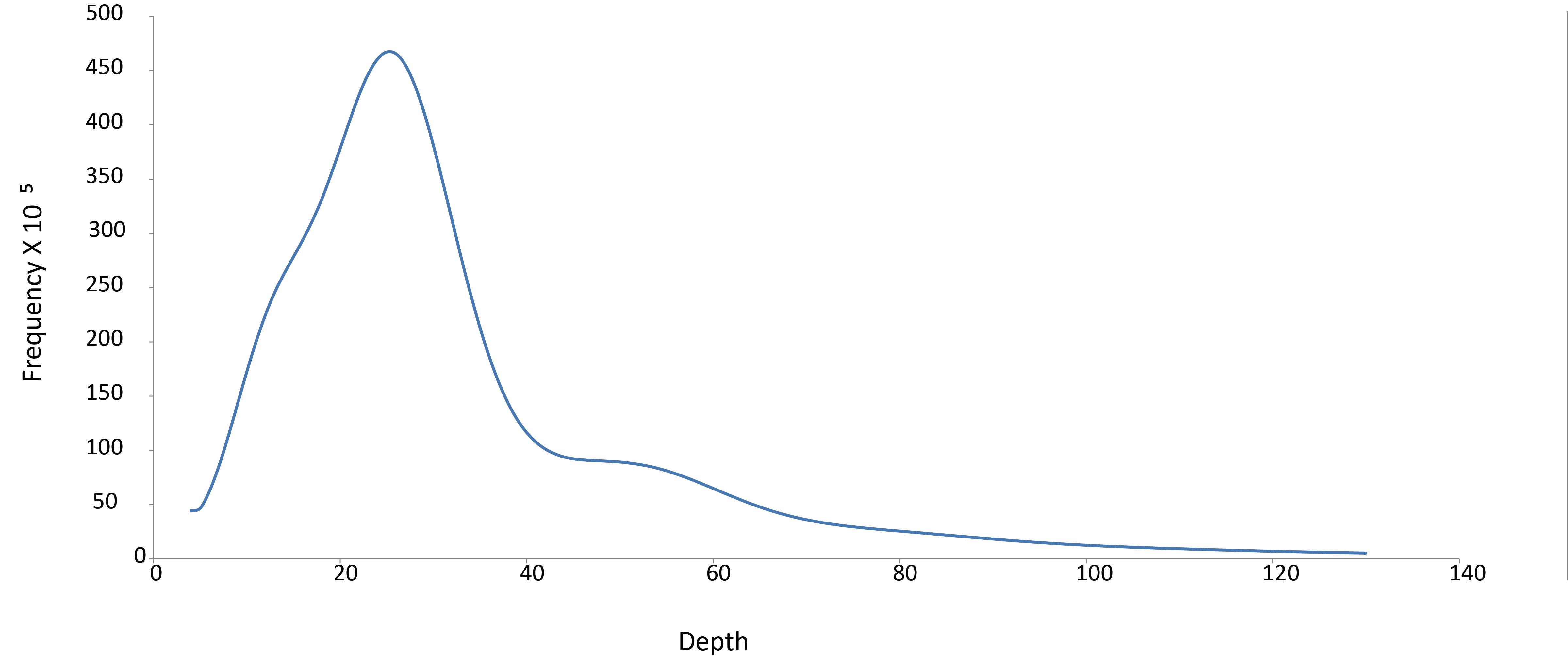

Supplement: Supplemental material [file giy038_supp.zip › Figure S1_kmer.tif]

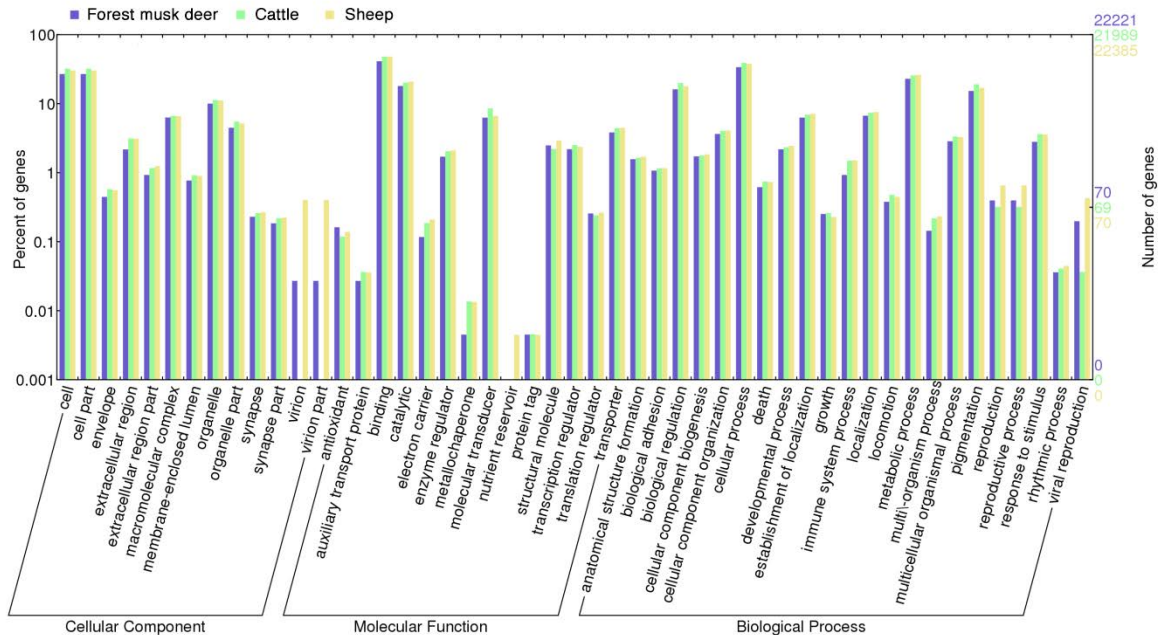

Supplement: Supplemental material [file giy038_supp.zip › Figure S2_wego.pdf]

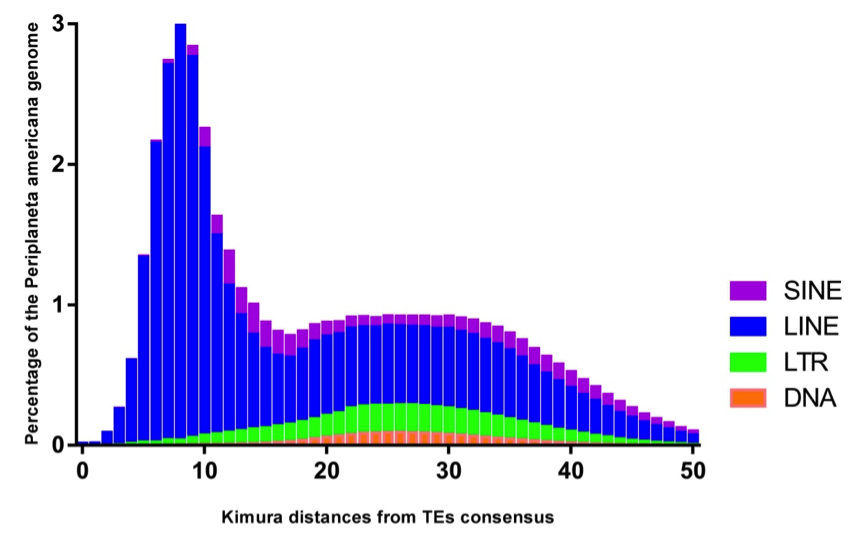

Supplement: Supplemental material [file giy038_supp.zip › Figure S3_TE_type.tif]

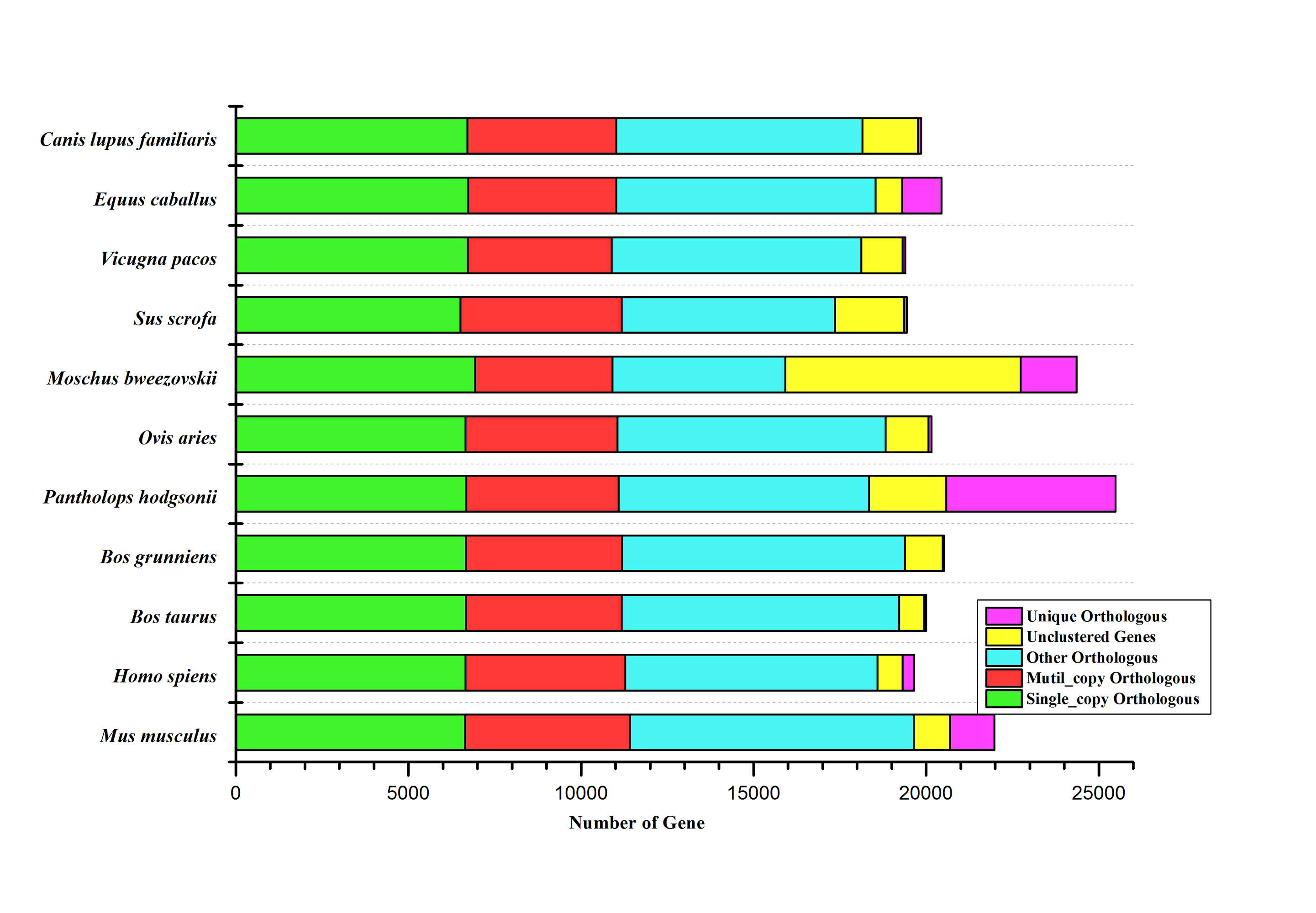

Supplement: Supplemental material [file giy038_supp.zip › Figure S4_OrthologousGene.tif]
