# Supplementary material for: The draft genome sequence of forest musk deer (Moschus berezovskii)
Source: Gigascience. 2018 Apr 9;7(4):giy038. doi: 10.1093/gigascience/giy038 (PMC5906906; doi:10.1093/gigascience/giy038)
Supplement: Response_to_Reviewer_Comments_Original_Submission.pdf [file giy038_response_to_reviewer_comments_original_submission.pdf]

Author's Response To Reviewer Comments

Close

Response to the comments

Reviewer #1
Overall the genome reflects the industry standard for sequencing and assembly. The paper reads as a series of independent analyses that have not been integrated or justified. Some add virtually nothing to the paper, while others need to be flushed out a bit further (i.e. more detail and justification). If there is a word constraint I have highlighted two sections that could be dropped thereby providing room to expand on other sections. The english is generally good but a careful proof by a native speaker would be useful.

1. Please provide the genome statistics after the short-read assembly, as this is useful information to the reader.
Response: We added the information into the manuscript (Lines 125 - 132) and Table 2.

2. Statements about length relative to other ungulates should be avoided, as this is dependent on the assembly quality and strategy. I do not find it biologically meaningful.
Response: We deleted them.

3. The TE analysis needs to be rethought; simply identifying repeats is not equivalent to identifying TEs. There is a severe lack of information provided here and it needs to be flushed out or dropped. How were the four types defined for exampled?
Response: We explained within the manuscript (Lines 218 - 232).

4. The Olfactory gene detection is not justified and it's not clear why this warrants it's own section.
Response: We combined the olfactory receptor genes section into the “Gene families” section, and simplified it (Lines 259 - 263).

5. The phylogenetic analysis does not add anything given there were no cervid genomes included; what was the point of this?
Response: Because there are no Cervidae genomes, we could not fully address the phylogenetic position of forest musk deer at the complete genome sequences level in our manuscript. However, the phylogenetic tree still gave us the general picture of the phylogenetic relationship between forest musk deer and its related species, which their genomes are available now, and the results also indicated that we should add the genome sequences from family Cervidae in the further investigation. Therefore, we still keep the phylogenetic analysis in the manuscript.

6. Again, no closely related species are included in the selection analysis. What was the point of this? Would you expect 500 genes to deviate from a closely related genome? Is this just chance?
Response: We agreed with both the editor and reviewer, the selection analysis is not very useful here and also need more work for it. Therefore, we removed the selection analysis section, and more focused on the sequencing, assembly and annotation parts.

Reviewer #2

General comments
In this article the authors present the first draft genome of the forest musk deer Moschus berezovskii. They provide a brief description of the sequencing, assembly and annotation process. This is a typical draft genome paper with little biological insight, but considering the status of the species and the little amount of available data on it, I believe that this contribution will be of great value to the community - if data and results are made available. I have two main concerns with this paper:

(1) I would need more details about the methods. It should be made possible to redo all the experiments and all the analyses that are mentioned in the manuscript. In particular, parameters and versions of each software tool have to be precised. Experimental protocols need more details too. One way to do this is to provide some more information in the main text and to complete with all the details in the Supplementary Methods. So far this document only contains the description of the phylogenetic analysis. The same should be done for the others.
Response: We added the parameters, software versions, and details of the protocols throughout the whole manuscript.

(2) The genomic sequence and its annotation should be made available. I could not find them. Sequencing reads have been deposited in the SRA but I would appreciate if the authors provide the results from the assembly (fasta format) and from the gene annotation (gtf or gff3 format for instance). This is actually the main value of the study.
Response: We have uploaded the information to GigaScience's database, and we have communicated with the editor to make sure all the data is available.

More specific comments to the authors
1. Sampling/sequencing
L112-114: please provide more details about library construction (DNA extraction, protocol, kit, etc). I expect some to be PE and others mate-pairs. Could you precise? Also, I am not sure the read length is specified.
Response: We extracted genomic DNA from tissue samples using the Qiagen DNeasy Blood and Tissue Kit (Qiagen, Valencia, USA) following the manufacturer's protocol. We added the information into the "Sample information and sequencing" section (Lines 112 - 119). We also added the read length information into the Table 1. The details were given in the new supplementary note.

L115-116: what kind of filtering/cleaning has been made and how? Please provide details about quality and adapter trimming, including the name of the software.
Response: We provided the details within the supplementary note (sequencing and filtering raw data section).

2. Assembly
L120-121: how did you estimate the genome size? Method, software, version? Why 17-mers?
Response: We used GCE (v1.0) to perform kmer analysis, and the 17-mer was suggested by the GCE results. The details could be found within the supplementary note.

L122: "the assembly was first analyzed by SOAPdenovo2" => don't you mean "generated"? The assembly needs to be produced before being analyzed.
Response: Yes, it should be "generated" and we corrected.

L125: what was the proportion of gaps before and after gapcloser?
Response: We explained it within the supplementary note.

L124: how was SSPACE used? How many scaffolds before and after? Also, please precise the version.
Response: We explained it within the supplementary note.

L129: Table 2 is way too short -only three numbers!- to give a decent description of the assembly. Please give the number of contigs, of scaffolds, the size of the longest ones, the GC%, etc…
See the same tables in similar publications from the same journal, for instance:
https://academic.oup.com/gigascience/article/4077042/The-draft-genome-sequence-of-a-desert-tree-Populus
https://academic.oup.com/gigascience/article/6/8/1/4004833/Draft-genome-of-the-Antarctic-dragonfish
https://academic.oup.com/gigascience/article/6/6/1/3748232/Draft-genome-of-the-lined-seahorse-Hippocampus
More generally, please also consider these previous publications to get an idea about the amount of details that are expected from this kind of report.
Response: We added more information within Table 2.

L132: CEGMA + BUSCO: Cegma is no longer maintained and should not be used anymore.
Response: Yes, we deleted the Cegma result and former Table S1 (the results for Cegma).

L136: please cite the study that generated the RNA-seq data you used.
Response: We added the SRA ID for the RNA-seq data (SRR2098995 and SRR2098996; Line 138) since there was no publication.

L138: the proportion of mapped reads is high and this is a good point, but it would be more informative to also show the proportion of concordant pairs, assuming that the RNA-seq data is PE. This assembly section is rather descriptive and technical but it is difficult to estimate up to which point all these steps were useful. A nice way to show the value of this work could be to compare the number of mapped reads and concordant read pairs from the RNA-seq and DNA-seq libraries (of the different sizes) before and after the gap filling and scaffolding part.
Response: In total of 92.73% PE reads were concordantly aligned to forest musk deer genome. We added the information into the manuscript (Lines 137 - 138).

3. Annotation
L147: how was Augustus trained? Was a training set of known genes provided to estimate the parameters?
Response: Yes, we used some protein sequences of Bos taurus to train the model for Augustus (version 3.0.3). We provided the information within supplementary note.

L148: "analyzed" => aligned, I guess
Response: Yes, we corrected the word.

L150-151: what is "software solar"? Please explain how GeneWise was used and provide details about potential filtering and other steps after the blast.
Response: We explained within the main text (Lines 178 – 180)

L153: Trinity has been used in both genome guided and de novo mode: why? What were the differences? Please provide the parameters. How did you merge the results?
Response: This method was followed by Trinity manual in the section of ' Build a Comprehensive Transcriptome Database Using Genome-guided and De novo RNA-Seq Assembly ', website is https://pasapipeline.github.io/.

L154: Please cite EVM properly. How did you use it? What did you choose for the confidence weights? Could you provide the input files from the distinct sources before merging?
Response: We cited the reference "Automated eukaryotic gene structure annotation using EVidenceModeler and the Program to Assemble Spliced Alignments, Genome Biology 2008". Other details could be found in the supplementary note.

L156: manual annotation can be a huge amount of work. Were there many modifications made? If so, it would be interesting and probably impressive to illustrate the contribution of this work by comparing annotation stats (see below) or other metrics before and after this polishing step, and/or to describe the most common corrections (gene splitting/merging? Splice site fixing? etc). That is only a suggestion.
Response: Yes, the manual annotation will require lots of work. Therefore, we only manually checked the scaffolds that size longer than 1Mb. In addition, we focused on gene splitting or merging.

Before the GO functional annotation, something that is missing is the description of the annotation with more statistics than just the number of genes, especially given the draft status of the genome assembly. In particular a simple table could present some stats about the gene length distribution (min, max, median, average), distribution of predicted ORFs/CDS (idem), number of exons per gene (idem: min, max, mean and median).
Response: We added a new supplementary table (current Table S2) to provide the length of the gene, CDS and exon.

Also, it would be useful to illustrate the quality of the provided annotation by comparing it with other available datasets. For instance, what is the percentage of RNA-seq mapped reads that fall within annotated exons? That are consistent with the predicted gene models? How do the annotated transcripts compare with those from the already published transcriptome study (ies)?
Response: Furthermore, we downloaded musk gland RNA-seq data (SRA accession: SRR2098995 and SRR2098996) of forest musk deer from NCBI to evaluate the assembly. We found that 99.3% of the total PE reads could be aligned (92.73% aligned concordantly) to the assembled forest musk deer genome by Bowtie2 (version 2.2.5). We added this within the main text (Lines 133 - 167).

4. Repeats
L190: "We also analyzed the degree of divergence for each type of TE" => How? Again: method, software, version, and parameters. Same for MSDB.
Response: We updated the method, software, version and parameters (Lines 219 – 239).

The number of TEs is compared across species: could the authors make sure that the same method was used to detect them in each species? Otherwise it could just be due to the method. Please keep in mind that all these annotated "genes", "TEs" etc, are only computational predictions.
Response: Yes, they were detected with different methods although the methods were very similar. We still should be careful when we compared the numbers.

5. Gene families
See general concern (1). Please also indicate the version of the ENSEMBL and NCBI annotation.
Response: Yes, we added the information into the table (current Table S5).

6. Olfactory Receptor genes
L218: typos in "pseudogenes" and “truncated".
Response: Thanks, we corrected the words.

L219: The number of OR genes is compared across species and a "degeneration of OR genes in primates" is mentioned. Couldn't the difference be due to the fact that these OR genes were not annotated the same way between species (Sup Tables)?
Response: Yes, therefore, we removed the comparison, and reviewer 1 also asked us to simplify this part (Lines 260 - 264).

Table S2: 19 / 303 = 20.51% ? ("missing busco" part)
Response: It was a mistake, we corrected the numbers and now, it is now within Table S1.

Table S5: It is ensembl, not ensemble. Please precise the version.
Response: We corrected the word, and also provided the version and other related information.

Close
