# Supplementary material for: The draft genome sequence of forest musk deer (Moschus berezovskii)
Source: Gigascience. 2018 Apr 9;7(4):giy038. doi: 10.1093/gigascience/giy038 (PMC5906906; doi:10.1093/gigascience/giy038)
Supplement: Response_to_Reviewer_Comments_Revision_1.pdf [file giy038_response_to_reviewer_comments_revision_1.pdf]

Author's Response To Reviewer Comments

Close

Reviewer #1

The paper by Fan et al. reports the genome assembly of the forest musk deer (Moschus berezovskii). The species is globally threatened and listed under CITES Appendix II, yet a relatively robust commercial farming (for musk) industry exists in China. Genomic resources will likely be informative for management, notably breeding programs and limiting disease transmission (Sun et al. 2018 Sci. Rep).

1. This is my second time reviewing the article and as noted previously, the genome assembly reflects the industry standard. The paper needs to be edited for spelling and grammar and I have listed some minor points below.
Response: We carefully checked the whole main text, supplementary notes and tables to improve the language.

2. Can the authors explain why they chose a male for the genome assembly? The homogametic sex is often selected for assembly in an effort to generate high enough coverage for the assembly of one of the sex chromosomes. If there is a reason, including oversight, I think this should be noted for subsequent groups interested in assembling non-model genomes.
Response: Only the male individuals can secrete the musk. One of the major aims for this genomic project is going to provide whole genome sequence to investigate potential pathway/regulation of musk secretion. Therefore, we chose to sequence and assemble a male individual.

3. L84-86: Awkward wording
Response: We have re-written the word as "In the last two centuries, hunting of all musk deer species significantly increased because of the commercially valuable of musk, which was an essential basis for perfume manufacture".

4. L89: hyphen unnecessary
Response: Thanks, we deleted the hyphen.

5. L100-102: Please provide a reference supporting disease severity being exacerbated by inbreeding and lack of genetic diversity.
Response: We added two references.
1. Zhao K, Liu Y, Zhang X, et al. Detection and characterization of antibiotic-resistance genes in Arcanobacterium pyogenes strains from abscesses of forest musk deer. J Med Microbiol. 2011;60:1820-6.
2. Huang J, Li Y, Li P, et al. Genetic quality of the Miyaluo captive forest musk deer (Moschus berezovskii) population as assessed by microsatellite loci. Biochemical Systematics & Ecology, 2013;47(8):25-30.

6. L103: Please clarify what genetic health means
Response: We mean the genomic information could be useful for the genetic management and disease prevention of the captive forest musk deer. To avoid misunderstanding, we have re-written the sentence.

7. L107-108: Needs revision
Response: Thanks, it was a mistake, we already removed selection and gene enrichments based on editor and reviewers' last comments. We have re-written this sentence.

8. L137: Is there a citation for the transcriptome data? Sun et al. generated transcriptomic data, and their analysis / story are relevant to this manuscript.
Response: The transcriptome data were used to evaluate the assembly and help the annotation. These data were uploaded to NCBI by Sichuan Agricultural University on July 2015. We did not find related publication. However, I contacted the author (submitter), and they said the paper had been published on Dec. 2017. They did not use the SSR numbers within the paper, thus we could not find it. Now, we cited their publication (Xu et al., 2017; Line 104 of the main text). The new paper (Sun et al., 2018) was published on January 2018 by other Chinese group. Therefore, we could not use their new data. However, we cited Sun et al.'s paper at the Introduction Section.

9. L139-L141. Delete - let the reader decided, based on the statistics provided, if this is a high quality genome
Response: We deleted this sentence.

10. L178. WEGO is not defined.
Response: We added the explanation. It is Web Gene Ontology Annotation Plot.

11. L182. Avoid the use of and/or; or will suffice 99% of the time.
Response: Thanks, we only keep the "or" in the sentence.

12. L189. That is your entire list of TEs, so "such as" is not required.
Response: We replaced "such as " as "including".

13. L233. "China's ecology" should be written differently.
Response: Thanks, it was a mistake, we have re-written the words as "Chinese ecology".

14. L236: (E)ndangered - should be lower case.
Response: Thanks, we have re-written the word.

Reviewer #2

The authors addressed most of my concerns. The editors provided the link to the Gigascience repository with the data.

Remaining comments:

1. Unanswered question: the EVM usage is not specified, nor is it mentioned in the Sup Notes. As this merging step was the one that generated the final annotation, according to the source field of the gff file, it would be useful to describe it.
Response: We added the information for EVM in Supplementary Notes: "Finally, EVM was used to

interpret all the above evidences, and the key parameters were as following: segmentSize = 1Mb, overlapSize = 20kb. The weight for de novo, homology and transcriptome-based gene predictions in EVM were set to 1, 5, and 10 respectively.”.

2. Sequencing and filtering: was cutadapt used with the same parameters for regular PE and mate-pair libraries? I am not sure it should be. Please precise. Was NGSQCToolkit used after cutadapt? Isn't there some redundancy with its adapter trimming step?
Response: NGSQCToolkit could not remove the adapters. The sequencing company (Novogene, China) had all the libraries based on the manufacturer’s protocol, thus Novogene had the adaptor information. They removed the adaptors and duplicate reads, then we ran NGSQCToolkit to further control the data quality. We added this explanation within the Supplementary Notes (section one).

3. 117: "A total of 407Gb of raw data were generated, after filtering out low quality, duplicate and adaptor polluted reads. Approximately 360Gb of high-quality reads were retained for genome assembly (Table 1)."
407Gb *after* filtering? Why 360Gb then?
Response: Sorry, our sentences were not clean. The raw data is about 407 Gb, and the clean data is about 360 Gb. We have re-written the sentences. Now, it is: “A total of 407Gb of raw data were generated. After filtering out low quality, duplicates and adaptor polluted reads, about 360Gb of high-quality reads were retained for genome assembly”.

4. The Supplementary Notes should be improved and proofread. Examples:
p.1: "sequencing data quality control was guide by ", "he re", "base- calling"
p.2: "were mapping to musk deer genome"
p.3: "were then aligned" ... "The script require"... "will concatenate" ... "It finally produces" (check the tense)
Response: We carefully checked the whole supplementary notes and tables to improve the language.

Close
