# Supplementary material for: The draft genome sequence of forest musk deer (Moschus berezovskii)
Source: Gigascience. 2018 Apr 9;7(4):giy038. doi: 10.1093/gigascience/giy038 (PMC5906906; doi:10.1093/gigascience/giy038)
Supplement: Reviewer_1_Report_(Original_Submission) -- Aaron Shafer — 9/13/2017 Reviewed [file giy038_reviewer_1_report_(original_submission).pdf]

Reviewer Report

Title: The draft genome sequence of forest musk deer (Moschus berezovskii)

Version: Original Submission **Date:** 9/13/2017

Reviewer name: Aaron Shafer

Reviewer Comments to Author:

Overall the genome reflects the industry standard for sequencing and assembly. The paper reads as a series of independent analyses that have not been integrated or justified. Some add virtually nothing to the paper, while others need to be flushed out a bit further (i.e. more detail and justification). If there is a word constraint I have highlighted two sections that could be dropped thereby providing room to expand on other sections. The english is generally good but a careful proof by a native speaker would be useful.Please provide the genome statistics after the short-read assembly as this is useful information to the readerStatements about length relative to other ungulates should be avoided as this is dependent on the assembly quality and strategy. I do not find it biologically meaningful. The TE analysis needs to be rethought; simply identifying repeats is not equivalent to identifying TEs. There is a severe lack of information provided here and it needs to be flushed out or dropped. How were the four types defined for exampled?The Olfactory gene detection is not justified and it's not clear why this warrants it's own section. The phylogenetic analysis does not add anything given there were no cervid genomes included; what was the point of this?Again, no closely related species are included in the selection analysis. What was the point of this? Would you expect 500 genes to deviate from a closely related genome? Is this just chance?

Level of Interest

Please indicate how interesting you found the manuscript: An article whose findings are important to those with closely related research interests

Quality of Written English

Please indicate the quality of language in the manuscript: Needs some language corrections before being published

Declaration of Competing Interests

Please complete a declaration of competing interests, considering the following questions:

- Have you in the past five years received reimbursements, fees, funding, or salary from an organisation that may in any way gain or lose financially from the publication of this manuscript, either now or in the future?
- Do you hold any stocks or shares in an organisation that may in any way gain or lose financially from the publication of this manuscript, either now or in the future?
- Do you hold or are you currently applying for any patents relating to the content of the manuscript?

- Have you received reimbursements, fees, funding, or salary from an organization that holds or has applied for patents relating to the content of the manuscript?
- Do you have any other financial competing interests?
- Do you have any non-financial competing interests in relation to this paper?

If you can answer no to all of the above, write 'I declare that I have no competing interests' below. If your reply is yes to any, please give details below.

No

I agree to the open peer review policy of the journal. I understand that my name will be included on my report to the authors and, if the manuscript is accepted for publication, my named report including any attachments I upload will be posted on the website along with the authors' responses. I agree for my report to be made available under an Open Access Creative Commons CC-BY license (http://creativecommons.org/licenses/by/4.0/). I understand that any comments which I do not wish to be included in my named report can be included as confidential comments to the editors, which will not be published.

I agree to the open peer review policy of the journal

To further support our reviewers, we have joined with Publons, where you can gain additional credit to further highlight your hard work (see: https://publons.com/journal/530/gigascience). On publication of this paper, your review will be automatically added to Publons, you can then choose whether or not to claim your Publons credit. I understand this statement.

Yes
