# Supplementary material for: The draft genome sequence of forest musk deer (Moschus berezovskii)
Source: Gigascience. 2018 Apr 9;7(4):giy038. doi: 10.1093/gigascience/giy038 (PMC5906906; doi:10.1093/gigascience/giy038)
Supplement: Reviewer_1_Report_(Revision_1) -- Aaron Shafer — 1/27/2018 Reviewed [file giy038_reviewer_1_report_(revision_1).pdf]

Reviewer Report

Title: The draft genome sequence of forest musk deer (Moschus berezovskii)

Version: Revision 1 Date: 1/27/2018

Reviewer name: Aaron Shafer

Reviewer Comments to Author:

The paper by Fan et al. reports the genome assembly of the forest musk deer (Moschus berezovskii). The species is globally threatened and listed under CITES Appendix II, yet a relatively robust commercial farming (for musk) industry exists in China. Genomic resources will likely be informative for management, notably breeding programs and limiting disease transmission (see Sun et al. 2018 Sci. Rep.). This is my second time reviewing the article and as noted previously, the genome assembly reflects the industry standard. The paper needs to be edited for spelling and grammar and I have listed some minor points below:Can the authors explain why they chose a male for the genome assembly? The homogametic sex is often selected for assembly in an effort to generate high enough coverage for the assembly of one of the sex chromosomes. If there is a reason, including oversight, I think this should be noted for subsequent groups interested in assembling non-model genomes. L84-86: Awkward wordingL89: hyphen unnecessaryL100-102: Please provide a reference supporting disease severity being exacerbated by inbreeding and lack of genetic diversity.L103: Please clarify what genetic health meansL107-108: Needs revisionL137: Is there a citation for the transcriptome data? Sun et al. generated transcriptomic data, and their analysis / story are relevant to this manuscript.L139-L141. Delete - let the reader decided, based on the statistics provided, if this is a high quality genome L178. WEGO is not defined. L182. Avoid the use of and/or; or will suffice 99% of the time.L189. That is your entire list of TEs, so "such as" is not required.L233. "China's ecology" should be written differently. L236: (E)ndangered - should be lower case.

Level of Interest

Please indicate how interesting you found the manuscript: An article whose findings are important to those with closely related research interests

Quality of Written English

Please indicate the quality of language in the manuscript: Needs some language corrections before being published

Declaration of Competing Interests

Please complete a declaration of competing interests, considering the following questions:

- Have you in the past five years received reimbursements, fees, funding, or salary from an organisation that may in any way gain or lose financially from the publication of this manuscript, either now or in the future?
- Do you hold any stocks or shares in an organisation that may in any way gain or lose financially from the publication of this manuscript, either now or in the future?

- Do you hold or are you currently applying for any patents relating to the content of the manuscript?
- Have you received reimbursements, fees, funding, or salary from an organization that holds or has applied for patents relating to the content of the manuscript?
- Do you have any other financial competing interests?
- Do you have any non-financial competing interests in relation to this paper?

If you can answer no to all of the above, write 'I declare that I have no competing interests' below. If your reply is yes to any, please give details below.

I declare that I have no competing interests

I agree to the open peer review policy of the journal. I understand that my name will be included on my report to the authors and, if the manuscript is accepted for publication, my named report including any attachments I upload will be posted on the website along with the authors' responses. I agree for my report to be made available under an Open Access Creative Commons CC-BY license (http://creativecommons.org/licenses/by/4.0/). I understand that any comments which I do not wish to be included in my named report can be included as confidential comments to the editors, which will not be published.

I agree to the open peer review policy of the journal

To further support our reviewers, we have joined with Publons, where you can gain additional credit to further highlight your hard work (see: https://publons.com/journal/530/gigascience). On publication of this paper, your review will be automatically added to Publons, you can then choose whether or not to claim your Publons credit. I understand this statement.

Yes
