# Supplementary material for: The draft genome sequence of forest musk deer (Moschus berezovskii)
Source: Gigascience. 2018 Apr 9;7(4):giy038. doi: 10.1093/gigascience/giy038 (PMC5906906; doi:10.1093/gigascience/giy038)
Supplement: Reviewer_2_Report_(Original_Submission) -- Sylvain Foissac — 9/19/2017 Reviewed [file giy038_reviewer_2_report_(original_submission).pdf]

Reviewer Report

Title: The draft genome sequence of forest musk deer (Moschus berezovskii)

Version: Original Submission **Date:** 9/19/2017

Reviewer name: Sylvain Foissac

Reviewer Comments to Author:

** General comments **In this article the authors present the first draft genome of the forest musk deer Moschus berezovskii. They provide a brief description of the sequencing, assembly and annotation process. This is a typical draft genome paper with little biological insight, but considering the status of the species and the little amount of available data on it, I believe that this contribution will be of great value to the community - if data and results are made available.I have two main concerns with this paper:(1) I would need more details about the methods. It should be made possible to redo all the experiments and all the analyses that are mentioned in the manuscript. In particular, parameters and versions of each software tool have to be precised. Experimental protocols need more details too. One way to do this is to provide some more information in the main text and to complete with all the details in the Supplementary Methods. So far this document only contains the description of the phylogenetic analysis. The same should be done for the others.(2) The genomic sequence and its annotation should be made available. I could not find them. Sequencing reads have been deposited in the SRA but I would appreciate if the authors provide the results from the assembly (fasta format) and from the gene annotation (gtf or gff3 format for instance). This is actually the main value of the study.As long as these two points are not addressed I cannot fully review this paper.----** More specific comments to the authors **- Sampling/sequencingL112-114: please provide more details about library construction (DNA extraction, protocol, kit, etc). I expect some to be PE and others mate-pairs. Could you precise? Also, I am not sure the read length is specified.L115-116: what kind of filtering/cleaning has been made and how? Please provide details about quality and adapter trimming, including the name of the software.- AssemblyL120-121: how did you estimate the genome size? Method, software, version? Why 17-mers?L122: "the assembly was first analyzed by SOAPdenovo2" => don't you mean "generated"? The assembly needs to be produced before being analyzed.L125: what was the proportion of gaps before and after gapcloser?L124: how was SSPACE used? How many scaffolds before and after? Also, please precise the version.L129: Table 2 is way too short -only three numbers!- to give a decent description of the assembly. Please give the number of contigs, of scaffolds, the size of the longest ones, the GC%, etc...See the same tables in similar publications from the same journal, for instance:https://academic.oup.com/gigascience/article/4077042/The-draft-genome-sequence-of-a-desert-tree-Populushttps://academic.oup.com/gigascience/article/6/8/1/4004833/Draft-genome-of-the-Antarctic-dragonfishhttps://academic.oup.com/gigascience/article/6/6/1/3748232/Draft-genome-of-the-lined-seahorse-HippocampusMore generally, please also consider these previous publications to get an idea about the amount of details that are expected from this kind of report.L132: CEGMA + BUSCO: Cegma is no longer maintained and should not be used anymore.L136: please cite the study that generated the RNA-seq data you used.L138: the proportion of mapped reads is high and this is a good point, but it would be more informative to also show the proportion of concordant pairs, assuming that the RNA-seq data is PE.This assembly section is rather descriptive and technical but it is difficult to estimate up to which point all these steps were useful. A nice way to show the value of this work could be to compare the number of mapped reads and concordant read pairs from the RNA-seq and DNA-seq libraries (of the different sizes) before and after the gap filling and scaffolding part.- AnnotationL147: how was Augustus trained? Was a training set of known genes provided to estimate the parameters?L148: "analyzed" => aligned, I guessL150-151: what is "software solar"? Please explain how GeneWise was used and provide details about potential filtering and other steps after the blast.L153: Trinity has been used in both genome_guided and de novo mode: why? What were the differences? Please provide the parameters.How did you merge the results?L154: Please cite EVM properly. How did you use it? What did you choose for the confidence weights? Could you provide the input files from the distinct sources before merging?L156: manual annotation can be a huge amount of

work. Were there many modifications made? If so, it would be interesting and probably impressive to illustrate the contribution of this work by comparing annotation stats (see below) or other metrics before and after this polishing step, and/or to describe the most common corrections (gene splitting/merging? Splice site fixing? etc). That is only a suggestion.Before the GO functional annotation, something that is missing is the description of the annotation with more statistics than just the number of genes, especially given the draft status of the genome assembly. In particular a simple table could present some stats about the gene length distribution (min, max, median, average), distribution of predicted ORFs/CDS (idem), number of exons per gene (idem: min, max, mean and median).Also, it would be useful to illustrate the quality of the provided annotation by comparing it with other available datasets. For instance, what is the percentage of RNA-seq mapped reads that fall within annotated exons? That are consistent with the predicted gene models? How do the annotated transcripts compare with those from the already published transcriptome study(ies)?- RepeatsL190: "We also analyzed the degree of divergence for each type of TE" => How? Again: method, software, version, parameters. Same for MSDB.The number of TEs is compared across species: could the authors make sure that the same method was used to detect them in each species? Otherwise it could just be due to the method. Please keep in mind that all these annotated "genes", "TEs" etc, are only computational predictions.- Gene familiesSee general concern (1). Please also indicate the version of the ENSEMBL and NCBI annotation.- Olfactory Receptor genesL218: typos in "pseudogenes" and "truncated".L219: The number of OR genes is compared across species and a "degeneration of OR genes in primates" is mentioned. Couldn't the difference be due to the fact that these OR genes were not annotated the same way between species (Sup Tables)?Table S2: 19 / 303 = 20.51% ? ("missing busco" part)Table S5: It is ensembl, not ensemble. Please precise the version.

Level of Interest

Please indicate how interesting you found the manuscript: An article whose findings are important to those with closely related research interests

Quality of Written English

Please indicate the quality of language in the manuscript: Needs some language corrections before being published

Declaration of Competing Interests

Please complete a declaration of competing interests, considering the following questions:

- Have you in the past five years received reimbursements, fees, funding, or salary from an organisation that may in any way gain or lose financially from the publication of this manuscript, either now or in the future?
- Do you hold any stocks or shares in an organisation that may in any way gain or lose financially from the publication of this manuscript, either now or in the future?
- Do you hold or are you currently applying for any patents relating to the content of the manuscript?
- Have you received reimbursements, fees, funding, or salary from an organization that holds or has applied for patents relating to the content of the manuscript?

- Do you have any other financial competing interests?
- Do you have any non-financial competing interests in relation to this paper?

If you can answer no to all of the above, write 'I declare that I have no competing interests' below. If your reply is yes to any, please give details below.

I declare that I have no competing interests.

I agree to the open peer review policy of the journal. I understand that my name will be included on my report to the authors and, if the manuscript is accepted for publication, my named report including any attachments I upload will be posted on the website along with the authors' responses. I agree for my report to be made available under an Open Access Creative Commons CC-BY license (http://creativecommons.org/licenses/by/4.0/). I understand that any comments which I do not wish to be included in my named report can be included as confidential comments to the editors, which will not be published.

I agree to the open peer review policy of the journal

To further support our reviewers, we have joined with Publons, where you can gain additional credit to further highlight your [illegible] (see: https://publons.com/journal/530/gigascience). On publication of this paper, your review will be automatically added to [illegible] can then choose whether or not to claim your Publons credit. I understand this statement.

Yes
